# Concept of Hybrid Drugs and Recent Advancements in Anticancer Hybrids

**DOI:** 10.3390/ph15091071

**Published:** 2022-08-28

**Authors:** Ankit Kumar Singh, Adarsh Kumar, Harshwardhan Singh, Pankaj Sonawane, Harshali Paliwal, Suresh Thareja, Prateek Pathak, Maria Grishina, Mariusz Jaremko, Abdul-Hamid Emwas, Jagat Pal Yadav, Amita Verma, Habibullah Khalilullah, Pradeep Kumar

**Affiliations:** 1 Department of Pharmaceutical Sciences and Natural Products, Central University of Punjab, Ghudda, Bathinda 154001, India; 2 Laboratory of Computational Modeling of Drugs, Higher Medical and Biological School, South Ural State University, 454008 Chelyabinsk, Russia; 3 Smart-Health Initiative (SHI) and Red Sea Research Center (RSRC), Division of Biological and Environmental Sciences and Engineering (BESE), King Abdullah University of Science and Technology (KAUST), Thuwal 23955, Saudi Arabia; 4 Core Laboratories, King Abdullah University of Science and Technology (KAUST), Thuwal 23955, Saudi Arabia; 5 Bioorganic and Medicinal Chemistry Research Laboratory, Department of Pharmaceutical Sciences, Sam Higginbottom University of Agriculture, Technology and Sciences, Prayagraj 211007, India; 6 Department of Pharmacology, Kamla Nehru Institute of Management and Technology, Faridipur, Sultanpur 228118, India; 7 Department of Pharmaceutical Chemistry and Pharmacognosy, Unaizah College of Pharmacy, Qassim University, Unayzah 51911, Saudi Arabia

**Keywords:** molecular hybridization, anticancer agents, cell lines, in vitro, pharmacophore

## Abstract

Cancer is a complex disease, and its treatment is a big challenge, with variable efficacy of conventional anticancer drugs. A two-drug cocktail hybrid approach is a potential strategy in recent drug discovery that involves the combination of two drug pharmacophores into a single molecule. The hybrid molecule acts through distinct modes of action on several targets at a given time with more efficacy and less susceptibility to resistance. Thus, there is a huge scope for using hybrid compounds to tackle the present difficulties in cancer medicine. Recent work has applied this technique to uncover some interesting molecules with substantial anticancer properties. In this study, we report data on numerous promising hybrid anti-proliferative/anti-tumor agents developed over the previous 10 years (2011–2021). It includes quinazoline, indole, carbazole, pyrimidine, quinoline, quinone, imidazole, selenium, platinum, hydroxamic acid, ferrocene, curcumin, triazole, benzimidazole, isatin, pyrrolo benzodiazepine (PBD), chalcone, coumarin, nitrogen mustard, pyrazole, and pyridine-based anticancer hybrids produced via molecular hybridization techniques. Overall, this review offers a clear indication of the potential benefits of merging pharmacophoric subunits from multiple different known chemical prototypes to produce more potent and precise hybrid compounds. This provides valuable knowledge for researchers working on complex diseases such as cancer.

## 1. Introduction

Cancer is a complex group of multiple diseases characterized by inappropriately controlled cell proliferation and replication eventually resulting in disruption of normal physiology, metabolism, or structure. Benign tumors are self-limited and do not invade or metastasize, but in complex stages groups of cells display uncontrolled growth, invasion and metastasis [1]. In the metastasis stage, cancer cells migrate from one organ (the original tumor site) to another organ of the body through the circulatory and lymphatic systems [2]. Cancer occurs by a series of successive deleterious mutations that change cell functions. These mutations often cause aberrant proliferation [3].

Cancer is a major global health care problem that continues to remain a leading cause of morbidity and mortality, with >277 different cancer types. According to estimates from the World Health Organization (WHO) in 2019, in 112 of 183 countries, cancer is the first or second major cause of death before the age of 70 and ranks third or fourth in a further 23 countries. In 2020, for all cancers, 19,292,789 new cases were estimated globally, with a total of 9,958,133 cancer deaths [4]. According to global demographic trends, 420 million new cancer cases are expected annually by 2025 [5]. 

Over the past 20 years, cancer treatments have improved significantly, and more potent medications have better safety profiles and more precise molecular targeting. Drug resistance is a major challenge with cancer treatment. During clinical usage, almost all targeted anticancer medications encounter resistance. Numerous processes have been related to drug resistance, including genetic and/or epigenetic mutation, amplification, cancer stem cells (CSCs), efflux transporters, apoptotic dysregulation, and autophagy, among others [6,7,8,9].

Cancer chemotherapy with single-agent or single mono functional ‘targeted’ drugs has limited rates of success due to resistance and lack of selectivity. To tackle this limitation, combination therapy (multi-component drugs to treat cancer), was developed [10]. Three alternative combination strategies are used: **(a)** two or more medicines that operate on distinct sites simultaneously or concurrently; **(b)** multi-targeting or promiscuous drugs treatment; and **(c)** hybridization drugs [11].

Tumor heterogeneity, drug-drug interactions, unpredictable pharmacokinetic (PK) safety profiles, and poor patient compliance presents a problem for cancer treatment despite the use of drug-combination medicines. Hence, improving drug selectivity while eliminating drug resistance has become crucial for the successful treatment of cancer patients [10].

There are unique challenges to cancer care in the developing as well as developed countries. These include issues with healthcare financing, patient awareness, and treatment delivery [6,7].

To improve the efficiency of using a two-drug cocktail, one approach involves so-called hybrid drugs [12]. The hybridization of biologically active molecules is a new concept and a powerful tool in drug design and development, used to target a variety of diseases [13]. It is a strategy of rational design of such ligands or prototypes based on the recognition of pharmacophoric sub-units that maintain pre-selected characteristics of the original templates [14].

Hybrid drugs are also termed “single molecule multiple targets” or “multiple ligands”. Hybrid molecules imply that one molecule shows structural features of two “parent” molecules. Two parent biologically active molecules (pharmacophores) that independently act at two distinct pharmacological targets. Molecular hybrids designed in a manner to the maintain their activities [15] by merging or blending of two or more bioactive compounds or their pharmacophoric subunits into in a new molecular structure with a dual mode of action. The presence of two or more pharmacophores in a single unit leads to a pharmacological potency greater than the sum of each individual moiety’s potencies [16,17].

Additionally, it is important to note that hybrid drugs usually have high molecular mass and lipophilicity; they violate Lipinski’s and Veber’s rules [17]. However, hybrid anticancer drugs have remarkable advantages over conventional anticancer drugs because they are designed to act on a different bio target or interact with numerous targets simultaneously, reducing the likelihood of drug-drug interactions, with reduced side effects and reduced propensity to elicit resistance relative to the parent drugs. These novel hybrid molecules have improved affinity, enhanced efficacy and improved safety [14,18].

Nowadays, hybrid drugs have drawn interest in the purposeful and logical design of ligands functioning selectively on multiple targets, and this has been reflected by an increase in the number of relevant publications in the field. In this review, we have compiled recent findings from 2011 to 2021 on novel hybrid compounds for different drug classes that exhibit promising anticancer activities. This analysis highlights in vitro anticancer activity of synthesized anticancer hybrids on different cell lines. 

## 2. The Concept of Hybrid Drugs in Anticancer Agent Development

Hybrid drugs are categorized based on the manner in which they are connected to each other. The concept of design and preparation of hybrid molecules is achieved using two strategies, which are described as follows: 

***1. Combining drug pharmacophoric moieties with*: (a)** two pharmacophoric groups directly linked; **(b)** two pharmacophoric groups linked by a spacer. 

These approaches are achieved by: **(i)** the merging of two pharmacophoric groups from two different drugs acting through the same mechanism of action; **(ii)** the merging of pharmacophoric groups from two drugs acting through different mechanisms of action.

**Figure 1:**
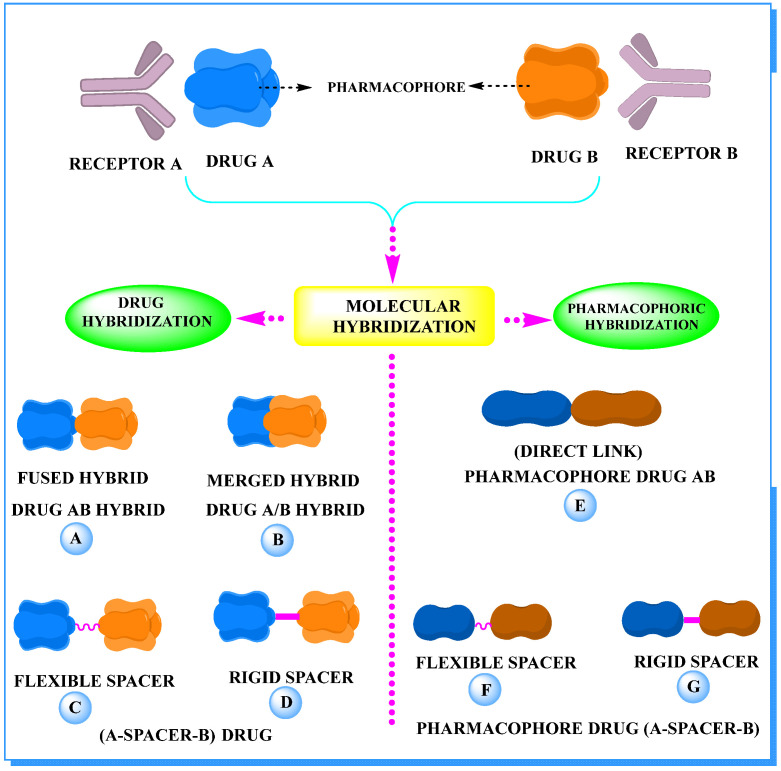
Different methods of molecular hybridization. (**A**) Drug A and B are directly linked to each other; (**B**) brug A and B are merged with each other; (**C**) drug A and B are connected by a flexible spacer; (**D**) drug A and B are connected through a rigid spacer; (**E**) two pharmacophoric moieties are directly connected to each other; (**F**) two pharmacophoric moieties are connected by a flexible spacer; (**G**) two pharmacophoric moieties are connected by a rigid spacer.

This is the integration of multiple pharmacophores in a single molecule. In this scenario, the starting point is to choose two established pharmacophores with high selectivity for their respective targets and a proper linker is selected to connect these two pharmacophores. This strategy is used to design new anticancer hybrids and is based on the ability of a combination of pharmacophoric moieties on a new molecular structure to retain their affinity and activity for the biological targets (Figure 1).

***2. Combining two or more entire drugs:*** the second approach combines two or more entire medications that can be connected either directly or indirectly using a spacer or linker. The following categories apply:

**(a)** Directly linked hybrid drugs: each molecule is connected via a functional group. Notable examples are mostly enzymatically hydrolysable esters, and carbamateoramide.

**(b)** Merged or overlapped hybrid drugs: these types of hybrid agents are obtained by overlapping structural motifs or pharmacophores of two drugs. These hybrid agents differ significantly in their structures compared to the drugs from which they were designed. The hybrid agents may retain the functional properties of either or both of the overlapping drugs.

**(c)** Spacer linked hybrid drugs: the main purpose of using a linker or spacer is to provide a bridge to connect two drugs and modulate the release of individual drugs in vivo. These molecules can be classified as cleavable and non-cleavable.

**(1)** Non-cleavable hybrid drugs: non-cleavable linkers are connected with non-hydrolysable chemical bonds to create chemically as well as enzymatically stable linkers. This strategy is also based on the ability of the different molecules to retain their biological activity, specificity and respective affinity for their biological targets.

**(2)** Cleavable hybrid drugs: a cleavable linker is based on the release of two parental molecular structures under physiological or enzymatic conditions that prevail at the site of activity. The majority of cleavable conjugates have an ester linkage that plasma esterases can cleave to release two separate medicines with independent actions (Figure 1).

The purpose of cleavable hybrid drugs is to either improve poor pharmacokinetic properties and slowly deliver the two therapeutic entities in the body (e.g., ester, amide or carbamate), or to improve the selectivity and the antineoplastic activity of the drugs and release the two drugs directly in the targeted tissues (e.g., phosphorylated DES (Diethylstilbestrol) prodrugs for prostate cancer).

Hybrids drugs have been formed by connecting two drugs with the same mechanism of action or by connecting two drugs with different mechanisms of action. The aim of connecting two drugs is to target specific biological tissues [12,19,20,21,22].

## 3. Recent Advances in Anticancer Hybrids

### 3.1. Quinazoline Based Hybrids

Quinazoline is a heterocyclic compound and potent bioactive scaffold that has been associated with anticonvulsant, anticancer, analgesic, sedative, anti-hypertensive, anti-inflammatory, anti-histaminic, antimicrobial, anti-viral and anti-tubercular properties. Quinazoline containing compounds were investigated for their inhibition of kinases, which are major explored targets of cancer medicine development [23,24].

**Figure 2:**
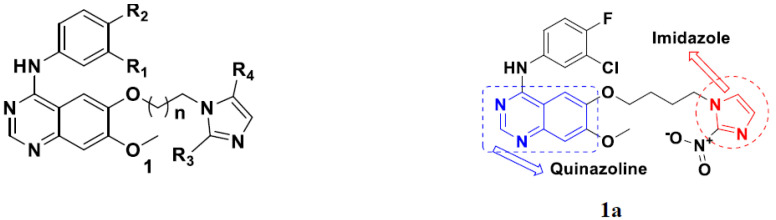
Structure of quinazoline-based imidazole hybrids and the most promising compound **1a**.

Cheng et al. (2015) synthesized and explored quinazoline based imidazole hybrids and evaluated their anticancer activity against Epidermal Growth Factor Receptor (EGFR) and HT-29 cells (in normoxic and hypoxic conditions). Most of the synthesized compounds displayed potent anticancer activity. Among them, compound **1**(**a**) showed excellent activity with an IC_50_ of 0.47 nM, 2.21 µM and 1.61 µM, respectively compared to the gefitinib control with an IC_50_ of 0.45 nM, 3.63 and 5.21 µM, respectively. The structure of quinazoline based imidazole hybrids is given in Figure 2. Table 1 shows in vitro antiproliferative activity against the human cancer cell line HT-29 and EGFR inhibitory activity (nM) of compounds **1**(**b**–**e**) [25].

**Figure 3:**
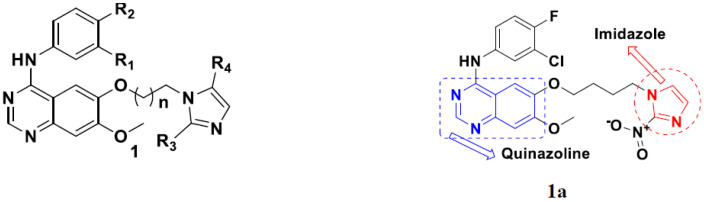
Structure of quinazoline-based deoxynojirimycin hybrids and the most promising compound **2a**.

Zhang Y et al. (2017) synthesized and evaluated anticancer activity of quinazolinebased deoxynojirimycin hybrids against Epidermal Growth Factor Receptor (EGFR) and α-glucosidase. Most of the synthesized compounds displayed potent anticancer activity. Among them, compound **2**(**a**) showed excellent activity with an IC_50_ 1.79 nM and 0.39 µM, respectively (the control gefitinib had an (−IC_50_ of = 3.32 nM and >100 µM, respectively). The structure of a quinazoline-based deoxynojirimycin hybrid is given in Figure 3 and in vitro EGFR and α-glucosidase inhibitory activity of compounds **2**(**b**–**e**) against human cancer cell lines is shown in Table 2 [26].

**Figure 4:**
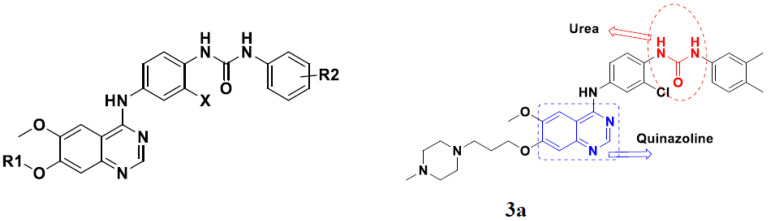
Structure of quinazoline-based urea hybrids and the most promising compound **3a**.

Quinazoline based urea hybrids were synthesized by Zhang et al. (2016) and evaluated for their ability to inhibit EGFR and Vascular Endothelial Growth Factor Receptor-2 (VEGFR-2). Most of the synthesized compounds displayed potent anticancer activity. Among them, compound **3**(**a**) showed excellent activity with an IC_50_ of 1.0 nM and 79 nM, respectively, when the control vandetanib had an IC_50_ of 11 nM and >15 nM, respectively. The structure of a quinazolinebased urea hybrid is given in Figure 4 and in vitro EGFR and VEGFR-2 inhibitory activity of compounds **3**(**b**–**e**) against human cancer cell lines is shown in Table 3 [27].

**Figure 5:**
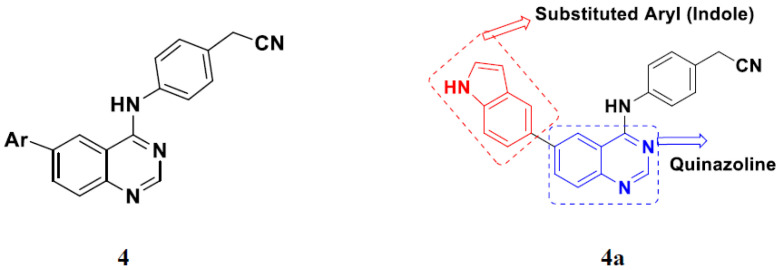
Structure of quinazoline-based aryl hybrids and the most promising compound **4a**.

Yadav et al. (2016) synthesized substituted quinazoline based aryl hybrids and evaluated their anticancer activity. Most of the synthesized compounds showed excellent activity against different isoforms of PI3K, but compound **4**(**a**) showed 3.7 times more potent activity against Phosphoinositide 3-Kinase(PI3K) α (IC_50_ = 0.201 μM) than γ isoform (IC_50_ = 0.75 μM), and was a selective inhibitor of MCF-7 cells (human breast adenocarcinoma) with GI50 7 μM. Compound **4**(**a**) also did not show any cytotoxicity to normal human cells. It inhibited 37% and 62% triglyceride (TGI) at 25 mg/kg dose in Ehrlich solid tumor and Ehrlich ascites carcinoma tumor models, respectively, with a control of 5-flurouracil at 22 and 20 mg/kg dose with TGI inhibition 50 and 96%, respectively. The structure of quinazoline-based aryl hybrids is given in Figure 5 and the in vitro cytotoxicity of compounds **4**(**b**–**e**) against human cancer cell lines is shown in Table 4 [28].

**Figure 6:**
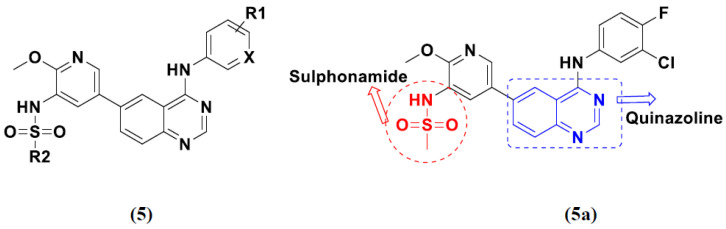
Structure of aminoquinazoline-sulphonamide hybrids and the most promising compound **5a**.

Ding et al. (2018) synthesized and reported aminoquinazoline-sulphonamide based hybrids and evaluated their anticancer activity. Most of these compounds showed dual inhibitory activity against EGFR and PI3K α. Compound **5**(**a**) showed excellent activity with an IC_50_ of 2.4 and 317 nM, compared to controls gefitinib (IC_50_ 2.4 nM) and dactolisib (IC_50_ 16.4 nM). Compound **5**(**a**) exhibited potent activity against a panel of cell lines including adenocarcinomic human alveolar basal epithelial cells (A549 IC_50_ = 8.23 µM), epithelial cells (BT549, IC_50_ = 1.02 µM), human colon cancer cells (HCT-116, IC_50_ = 5.60 µM), breast cancer cells (MCF-7, IC_50_ = 5.59 µM), human hepatic adenocarcinoma cells (SK-HEP-1, IC_50_ = 6.10 µM), and gastric carcinoma cells (SNU638, IC_50_ = 4.10 µM). For comparison, the controls gefitinib and dactolisib had IC_50_ doses of 8.27, 6.56, 5.98, 26.7, 10.1, and 7.56 µM or 0.62, 0.74, 0.84, 1.33, 1.82,and 1.24 µM in the same cell lines), respectively. The structure of aminoquinazoline-sulphonamide based hybrids is given in Figure 6 and in vitro cytotoxicity (IC_50_, μM) of compounds **5**(**b**–**e**) against human cancer cell lines is shown in Table 5 [29].

**Figure 7:**
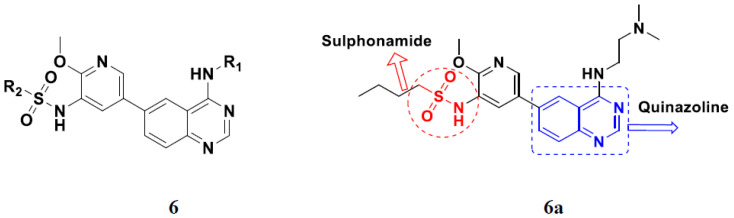
Structure of quinazoline-amino sulphonamide based hybrids and the most promising compound **6a**.

Continuing the work of Ding et al. (2018), Fan et al. synthesized amino quinazoline based hybrids and evaluated their anticancer activity. Most of these compounds showed inhibitory activity against PI3K. Compound **6**(**a**) showed excellent activity towards PI3Kα with an IC_50_ 13.6 nM in comparison to other isoforms of PI3K including PI3Kβ, PI3Kγ and PI3Kδ, which had IC_50_ of396.2, 117.5, and 101.8 nM. Compound **6**(**a**) exibited potent activity against a panel of cell lines including HCT-116, SK-HEP1, MDA-MB-231 (epithelial, human breast cancer cells), SNU638, A549, and MCF-7 with IC_50_ values 0.16, 0.28, 0.28, 0.48, 1.32, and 3.24 µM. Control BEZ235 had IC_50_ values of 0.84, 1.82, 0.18, 1.24, 0.62, and 1.33 µM. The structure of quinazoline-amino sulphonamide based hybrid is given in Figure 7 and in vitro cytotoxicity (IC_50_, μM) of compounds **6**(**b**–**e**) against human cancer cell lines is shown in Table 6 [30].

**Figure 8:**
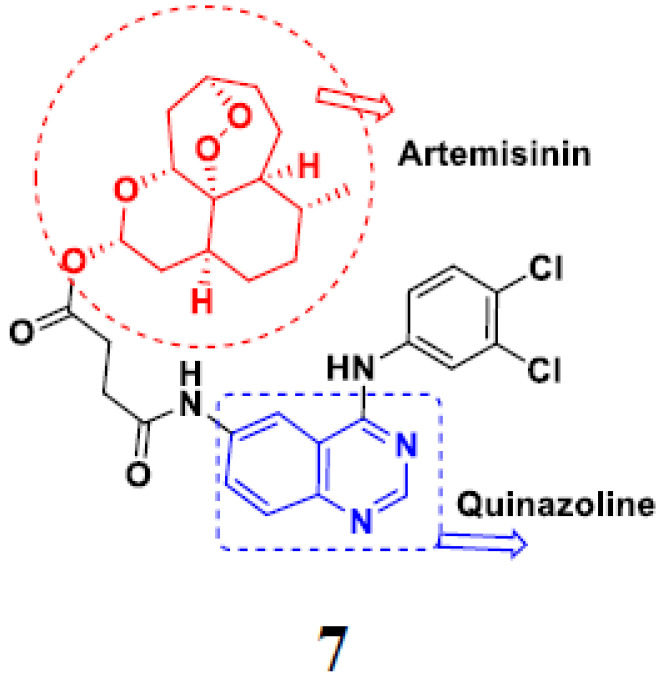
Structure of quinazoline-artemisinin based hybrid **7**.

Frohlich et al. (2017) synthesized hybrids of quinazoline with artemisinin and evaluated their in vitro anticancer activity on CCRF-CEM (lymphoblastoid cell) and CEM/ADR5000 (leukemia cells). Most of the synthesized compounds showed potent anticancer activity; out of them, compound **7** (Figure 8) showed excellent activity with an EC_50_ of 2.8 and 0.6 µM. Control doxorubicin had an EC_50_ of 0.009 and 23.27 µM, respectively [31].

**Figure 9:**
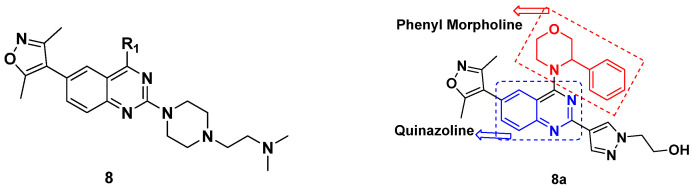
Structure of quinazoline phenyl morpholine based hybrids and the most promising compound **8a**.

Yang et al. (2018) synthesized quinazoline based hybrids and evaluated them for their Bromodomain-containing protein 4 (BRD4) inhibitory activity. SAR studies revealed that the phynylmorpholine along with the pyrazole skeleton showed potent activity against BRD4. Compound **8**(**a**) showed potent activity against human AML cells (MV4-11) with an IC_50_ value of 1.10 µM (K_d_ 66 nM) when control BET760 had an IC_50_ 0.80 µM (K_d_ 37 nM). The structure of quinazoline-phenyl morpholine based hybrids is given in Figure 9 and in vitro cytotoxicity of compounds **8**(**b**–**e**) against human cancer cell lines is shown in Table 7 [32].

#### Quinazoline-Based Hybrids That Are FDA Approved or under Clinical Trial

**Figure 10:**
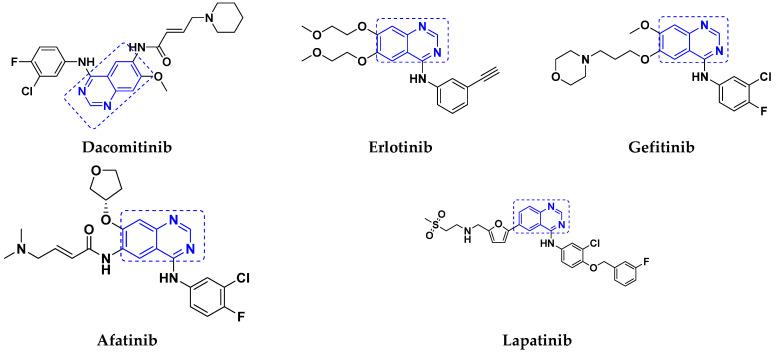
FDA approved/clinical trial drugs with quinazoline hybrids.

The hybrids of quinazoline have been evaluated in various clinical trials in recent years, with several of them showing promising results. In addition, the Food and Drug Administration (FDA) approved certain quinazoline-based enzyme inhibitors for the management of various malignancies. Dacomitinib, erlotinib, gefitinib, afatinib, and lapatinib are the quinazoline based hybrid drugs approved by the FDA for management of different types of cancers (Figure 10). Additionally, in Table 8, we have summarized quinazoline-based hybrid molecules under clinical trials for the treatment of different types of cancer.

### 3.2. Indole-Based Hybrids

Indole is a remarkable and adaptable heterocycle that has been used to create important biological scaffolds in pharmaceutical research. Indole/indole derivatives have many reported therapeutic properties, such as anti-virals, anti-convulsants, antibacterials, anti-microbials, anticancer agents, anti-malarials, anti-inflammatories, anti-oxidants, and anti-diabetics. Different natural and synthesized indole motifs have demonstrated considerable anticancer potential. Such substances have been found to act on a variety of protein targets, including sirtuins, PIM (proviral integration site for Moloney murine leukemia virus) kinases, HDACs (histone deacetylases) and DNA topoisomerase [41,42].

**Figure 11:**
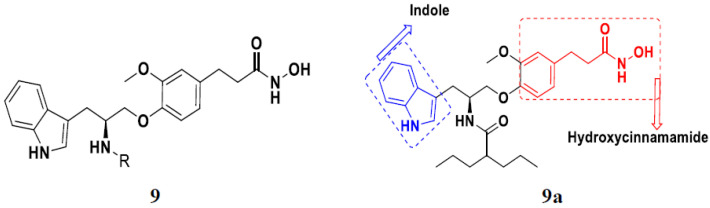
Structure of indole with hydroxycinnamamide hybrid and the most promising compound **9a**.

Zhang et al. (2013) synthesized hybrids of indole with hydroxycinnamamide, and evaluated their anticancer activity against different cell lines. Most of the synthesized compounds showed potent anticancer activity. Among them, compound **9**(**a**) exhibited excellent activity against various cell lines including human myeloid leukemia (U937), prostate adenocarcinoma (PC-3), A549, ovarian carcinoma (ES-2), MDA-MB-231, and HCT116 at IC_50_ 1.8, 3.7, 4.4, 5.4, 3.1, and 5.5 µM, respectively with control suberoylanilide hydroxamic acid (SAHA) had IC_50_ values of 2.3, 9.9, 3.8, 12.7, 5.6, and 6.0 µM, respectively. Furthermore, compound **9**(**a**) showed excellent HDAC (histone deacetylase) inhibitory activity against different isoforms of HDAC including HDAC1, HDAC2, HDAC3, and HDAC6 with IC_50_ values of 0.39, 1.42, 0.28, and 0.94 µM compared to control SAHA at IC_50_ values of 0.076, 0.256, 0.028, and 0.118 µM, respectively. The structure of indole with hydroxycinnamamide hybrids is shown in Figure 11 and in vitro cytotoxicity of compounds **9**(**b**–**e**) against human cancer cell lines is shown in Table 9 [43]. 

**Figure 12:**
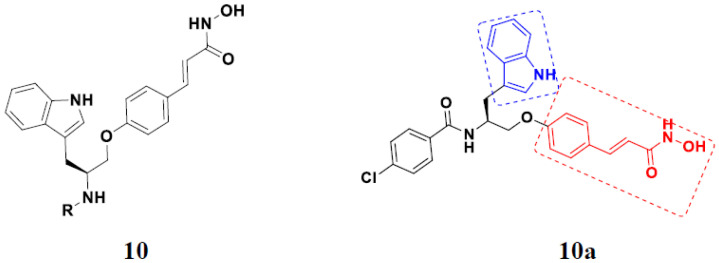
Structure of indole with hydroxycinnamamide hybrids and the most promising compound **10a**.

Zhang et al. (2013) synthesized and explored hybrids of indole with hydroxycinnamamide, evaluating their anticancer activity against different cell lines. Most of the synthesized compounds showed potent anticancer activity. Among them, compound **10**(**a**) exhibited excellent activity against various cell lines including U937, K562 (myelogenous leukemia cells), HEL (human erythroleukemia cells), KG1(myeloid leukemia cells), HL60 (promyelocytic leukemia cells), MDA-MB-231, PC-3, MCF-7, HCT116, and A549 with IC_50_ values 0.16, 0.51, 0.19, 0.22, 1.69, 0.22, 0.46, 2.68, 0.52, and 2.74 µM, respectively. Furthermore, compound **10a** showed excellent HDAC selectivity against different isoforms of HDAC including HDAC1, HDAC2, HDAC3, and HDAC6 with IC_50_ values 11.8, 498.1, 3.9, and 308.2 nM with control SAHA had IC_50_ values of 34.6, 184.7, 90.1, and 63.0 nM, respectively. The T structure of indole and hydroxycinnamamide hybrids is shown in Figure 12 and the in vitro cytotoxicity of compounds **10**(**b**–**e**) against human cancer cell lines is shown in Table 10 [44]. 

**Figure 13:**
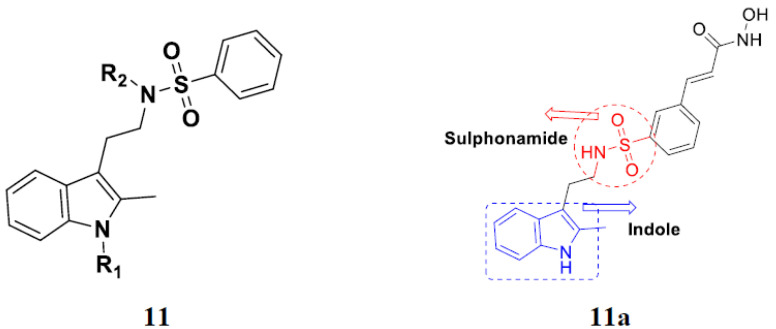
Structure of indole with sulphonamide hybrids and the most promising compound **11a**.

Mehndiratta et al. (2014) synthesized hybrids of indole with sulphonamides, and evaluated their anticancer and anti-inflammatory activity. Most of the synthesized compounds showed potent anticancer activity. Among them, compound **11**(**a**) showed excellent activity against HeLa nuclear HDAC enzyme with an IC_50_ of 7.9 nM. The structure of indole and sulphonamide hybrids is shown in Figure 13 and the in vitro cytotoxicity of compounds **11**(**b**–**e**) against human cancer cell lines is shown in Table 11 [45]. 

**Figure 14:**
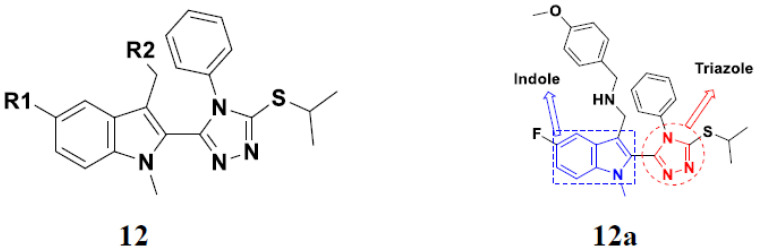
Structure of indole-triazole based hybrids and the most promising compound **12a**.

Panathur et al. (2013) synthesized indole based hybrids, and evaluated them for anticancer activity against three cancer cell lines including K562, MDA-MB 231 and LNCaP (androgen-sensitive human prostate adenocarcinoma cells). Most of the synthesized compounds showed potent anticancer activity; among them, 9 compounds showed excellent activity against MDA-MB 231, with three compounds inhibiting growth of LNCaP cell up to 50% at 10 µM. Furthermore, the lead molecule **12**(**a**) showed SIRT1 (Sirtuin 1) inhibitory activity up to 70% with control finasteride (growth inhibition 56%) at 40 µM. The structure of indole-triazole based hybrids is given in Figure 14 and the in vitro cytotoxicity of compounds **12**(**b**–**e**) against human cancer cell lines is shown in Table 12 [46].

**Figure 15:**
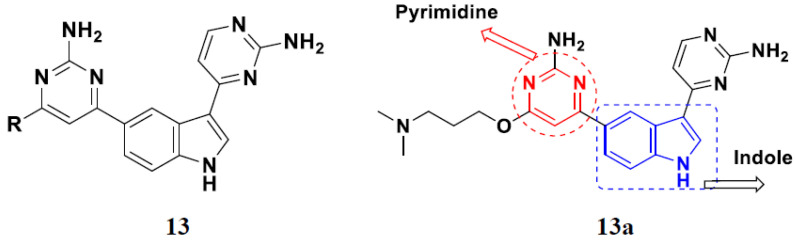
Structure of indole-pyrimidine based hybrids and the most promising compound **13a**.

Lee et al. (2014) synthesized indole based hybrids and evaluated their proto-oncogene serine/threonine-protein kinase (PIM) kinase selectivity against different forms of PIM kinase including PIM1, PIM2, and PIM3. Compound **13**(**a**) showed excellent selectivity towards PIM1 with IC_50_ values of 0.058, 0.52, and 0.16 µM. Furthermore, the lead molecule 13(**a**) was tested against different cell lines, including MV-4-11 (acute myeloid leukemia cell), Jurkat (T lymphocyte cells), and K562, and was found to be very selective towards MV-4-11 in a cell viability assay. The lead molecule also showed binding interaction with Lys67 and Glu89 in the active site of PIM1 (ATP-binding). The structure of an indole-pyrimidine based hybrid is shown in Figure 15, and the in vitro cytotoxicity of compounds **13**(**b**–**e**) against human cancer cell lines is shown in Table 13 [47].

**Figure 16:**
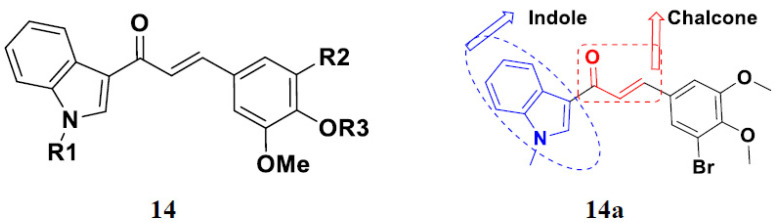
Structure of indole-chalcone based hybrids and the most promising compound **14a**.

Mirzaei et al. (2017) synthesized and explored indole-chalcone based hybrids and evaluated their anticancer activity against various cell lines including A549, MCF7, SKOV3 (human ovarian cancer cell), and NIH3T3 (embryonic fibroblast cells), finding IC_50_ values of 4.3, 100, 20.2, and 154.6 µg/mL with control etoposide IC_50_ of 7.8, 9.9, 8.5, and 118.0 µg/mL, respectively. Furthermore, the lead molecule 14(**a**) was subjected to a tubulin polymerization inhibitory assay, with results revealing that the lead molecule showed excellent inhibitory activity, having an IC_50_ of 17.8 µM with control colchicine, having an IC_50_ of 2.3 µM. The structure of indole-chalcone based hybrids is shown in Figure 16 and the in vitro cytotoxicity of compounds **14**(**b**–**e**) against human cancer cell lines is shown in Table 14 [48]. 

**Figure 17:**
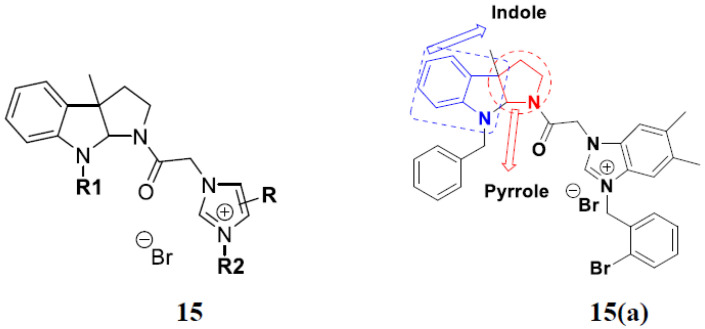
Structure of indole-pyrole based hybrids and the most promising compound **15a**.

Zhou et al. (2016) synthesized and explored hybrids of indole with pyrrole and evaluated their anticancer activity against various cell lines including HL-60, SMMC-7721 (hepatocarcinoma cells), A-549, MCF-7, and SW480 (colon carcinoma cell). Most of the synthesized compounds showed potent anticancer activity. Among them, compound **15**(**a**) showed excellent activity having IC_50_ values of 1.27, 1.72, 2.68, 1.78, and 1.44 µM with control cisplatin (DDP) having IC_50_ values of 1.16, 8.08, 7.10, 10.45, and 8.88 µM, respectively. The structure of indole-pyrole based hybrids is shown in Figure 17 and the in vitro cytotoxicity of compounds **15**(**b**–**e**) against human cancer cell lines is shown in Table 15 [49]. 

**Figure 18:**
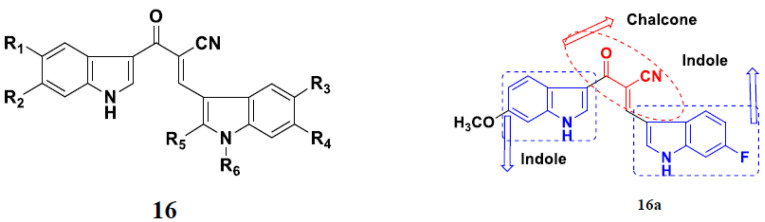
Structure of indole-chalcone based hybrids and the most promising compound **16a**.

Kumar et al. (2014) synthesized indole based chalcone hybrids and evaluated their antiproliferative activity against A549, PC3 and PaCa2 (pancreatic cancer) cell lines. Among the synthesized derivatives, compound 16(**a**) showed potent activity having IC_50_ values of 2.4 and 0.8, 36.0 and 22.5, and >50 µM, respectively (with control mitomycin C having an (IC_50_ = of 0.45 µM against A549 at 24h). The structure of indole-chalcone based hybrids is shown in Figure 18 and the in vitro cytotoxicity of compounds **16**(**b**–**e**) against human cancer cell lines is shown in Table 16 [50]. 

**Figure 19:**
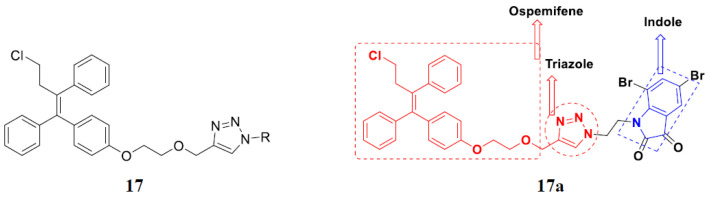
Structure of indole-ospemifene-triazole based hybrids and the most promising compound **17a**.

Kumar et al. (2018) synthesized and explored indole-ospemifene-triazole based hybrids and evaluated their anticancer activity against MCF-7 and MDA-MB-231. Most of the synthesized compounds showed good anticancer activity, compound **17**(**a**) exhibited excellent activity at IC_50_ 1.56 and 48.46 µM controls included ospemifene (IC_50_ 55 and 50 µM), tamoxifen (IC_50_ 3.5 and >100 µM), and plumbagin (IC_50_ 75 and 4.4 µM). The structure of the indole-ospemifene-triazole based hybrid is shown in Figure 19 and the in vitro cytotoxicity of compounds **17**(**b**–**e**) against human cancer cell lines is shown in Table 17 [51]. 

**Figure 20:**
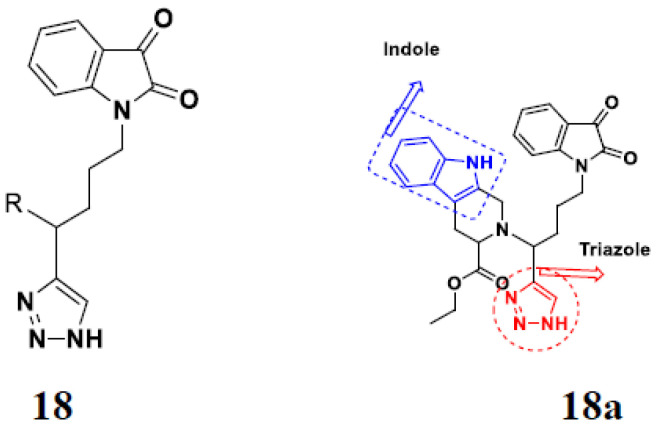
Structure of indole-ospemifene-triazole based hybrids and the most promising compound **18a**.

Kumar et al. (2018), and Sharma et al. (2019) synthesized indole-isatin based triazole hybrids and evaluated their anticancer activity. Among the synthesized compounds, compound 18(**a**) showed excellent activity against MCF-7 and MDA-MB-231 cell lines withIC_50_ values of 37.42 and >100 µM Control included plumbagin (IC_50_ 3.5 4.4 µM), peganumine A (IC_50_ 38.5 µM and not observed) and tamoxifen (IC_50_ 50 and 75 µM), respectively. Furthermore, the biological activity was validated by docking studies. The structure of the indole-isatin-triazole based hybrid is shown in Figure 20, and the in vitro cytotoxicity (µM) of compounds **18**(**b**–**c**) against human cancer cell lines is shown in Table 18 [52].

### 3.3. Indole-Based Hybrids That Are FDA Approved or under Clinical Trial

**Figure 21:**
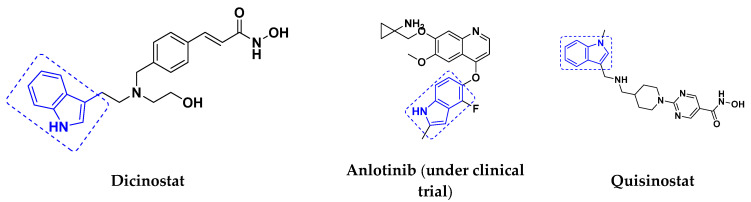

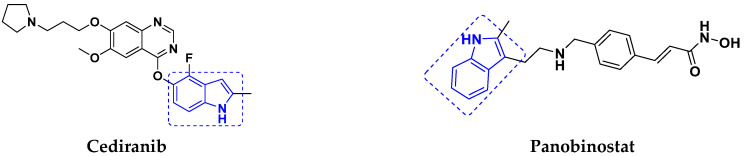
FDA approved/clinical trial drugs with indole hybrids.

Dacinostat (LAQ824), an indole-based hybrid molecule, is approved for the management of breast and prostate cancer, whereas panobinostat (LBH-589) is a marketed medicine for numerous malignancies. Quisinostat (JNJ-26481585), a synthetic indole-hydroxamic acid molecule (hybrid) with putative antitumor action is an orally available 2nd generation molecule to inhibit HDAC. Cediranib is a tyrosine kinase inhibitor which affects the function and development of endothelial cells in human kidney tumors. Anlotinib (AL3818) is a potent kinase inhibitor with potential antitumor as well as antiproliferative efficacy currently in clinical trials. Figure 21, shows chemical structures of indole-based FDA approved/clinical trial drugs and Table 19 gives descriptions of them.

### 3.4. Carbazole Based Hybrids

Carbazole is a key scaffold found in a wide range of physiologically potent chemicals, including natural and synthetic analogues. Anticancer, antifungal, antibacterial, anti-HIV, anti-inflammatory, anti-protozoan, anti-psychotic, antidiabetic and anticonvulsant properties are found in molecules with r chemically modified carbazole moiety. The first carbazole compounds, celiptium and ellipticine, targeted topoisomerase II and cytochrome P450, respectively, and are used to combat cancer (metastatic breast cancer). An investigation of the carbazole nucleus for the production of novel structural scaffolds has led to the development of a number of available anti-tumor hybrids [58,59].

**Figure 22:**
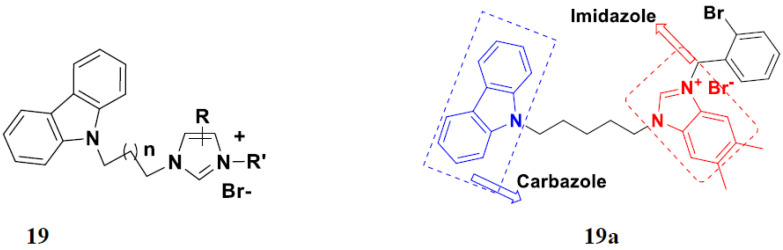
Structure of carbazole-imidazole based hybrids and the most promising compound **19a**.

Liu et al. (2015) synthesized and evaluated the anticancer activity of carbazole derivatives against various cell lines including HL-60, SMMC-7721, A549, MCF-7 and SW480. Among the synthesized carbazole derivatives, compound 19(**a**) showed potent activity with IC_50_ values of 0.51, 2.38, 3.12, 1.40, and 2.48 µM, where control cisplatin (DDP) hadIC_50_ values of 1.32, 6.24, 11.83, 15.17, and 12.95 µM, respectively. Compound **19**(**a**) showed cell cycle arrest in SMMC-7721 cells. The structure of carbazole-imidazole based hybrids is shown in Figure 22 and the in vitro cytotoxicity of compounds **20**(**b**–**e**) against human cancer cell lines is shown in Table 20 [60]. 

**Figure 23:**
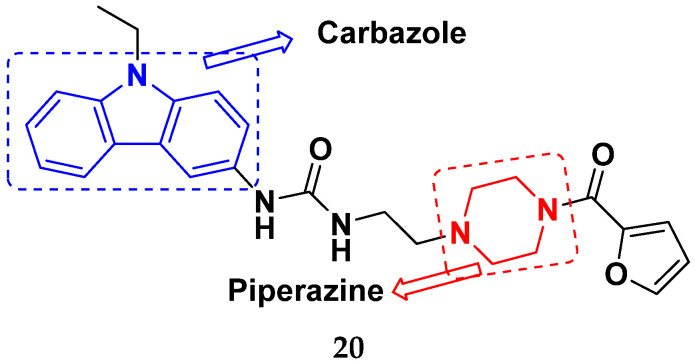
A carbazole-piperazine hybrid (**20**).

Mongre et al. (2019) synthesized a potent novel hybrid (**20**) of carbazole and piperazine and evaluated its anticancer activity against various cell lines including A549, NCI-H1299 (non-small cell lung carcinoma cells), HT-29, MCF-7, Hela (cervical carcinoma), and U2OS (osteosarcoma cells). The IC_50_ values in these cell lined were 1.779, 2.270, 2.20, 2.637, 3.072 and 2.739 µM, respectively. The molecule also showed potent cell cycle arresting activity at concentrations of 0.5, 1.0 and 2.0 µM by affecting G2/M cell cycle transition. Hybrid (**20**) also inhibited tumor progression in a xenograft model (BALB/c-nu nude mouse) at a dose of 3 mg/kg body weight without any toxicity. A carbzole-piperazine hybrid is depicted in Figure 23 [61]. 

#### Carbazole-Based Hybrids That Are FDA Approved or under Clinical Trial

Some well-known carbazole-based hybrids with potential anticancer effects have been published [59,62,63,64]. Some carbazole-based hybrids are FDA approved medications or in clinical studies for cancer treatment. Alectinib was licensed by the US Food and Drug Administration (FDA) and the European Medicines Agency (EMA) in 2015 for the management of anaplastic lymphoma kinase-positive progressive non-small cell lung cancer (NSCLC). Midostaurin is a carbazole hybrid that was approved by the FDA and the European Medicines Agency (EMA) in 2017. It is used to treat recently diagnosed advanced systemic mastocytosis and acute myeloid leukemia [65,66].

**Figure 24:**
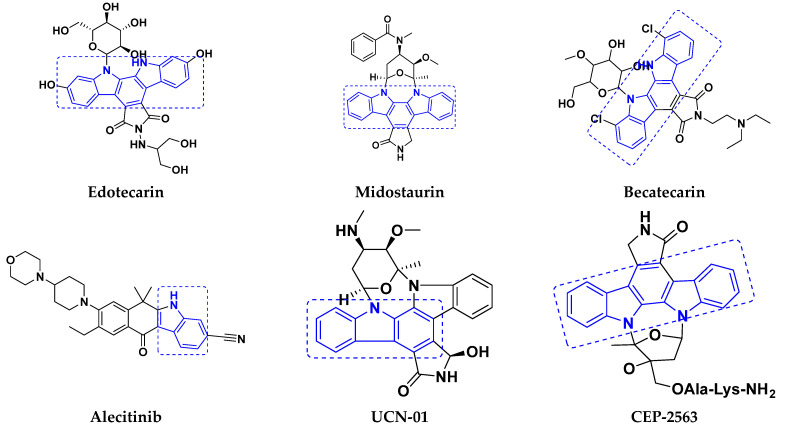
Carbazole hybrids that are FDA approved/or under clinical trials.

Currently, four hybrids of carbzole are being tested in clinical studies including becatecarin and edotecarin, which have been proven to intercalate DNA and maintain the DNA-topo I complex, and are currently in Phase II and Phase III clinical studies, respectively. CEP-2563 is a strong inhibitor of platelet derived growth factor (PDGF) and tyrosine kinase that is now in a Phase I clinical development for medullary thyroid cancer. UCN-01 is now being tested in a Phase II clinical study for breast cancer, lymphoma, and pancreatic by acting upon protein kinases. The structure of FDA approved/clinical trials drugs with carbazole hybrids is given in Figure 24 and their current status is shown in Table 21.

### 3.5. Pyrimidine-Based Hybrids

The basic structure of RNA, DNA, and nucleic acids is the heterocyclic pyrimidine ring. Pyrimidine and its conjugated counterparts possess antiviral, antibacterial, anticancer, analgesic, anti-inflammatory, antimalarial and antioxidant activities. Anticancer action is the most often described therapeutic property of pyrimidine because it interacts with a variety of targets, as well as receptors to cause cell death. Multiple studies on the anticancer efficacy of pyrimidine compounds, as well as their therapeutic use, have supported their position as a prospective drug development nucleus [70,71].

**Figure 25:**
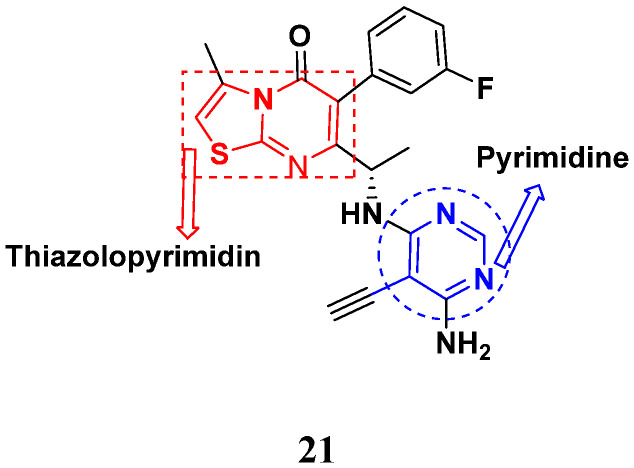
A pyridine hybrid (**21**).

Combs et al. (2015) synthesized and patented aminopyrimidine hybrids and evaluated their P13K enzyme as well as cell proliferation inhibitory potential in 96-well plates using scintillation counting. Compound **21** (Figure 25) showed potent activity with IC_50_ < 20 nM [72]. 

**Figure 26:**
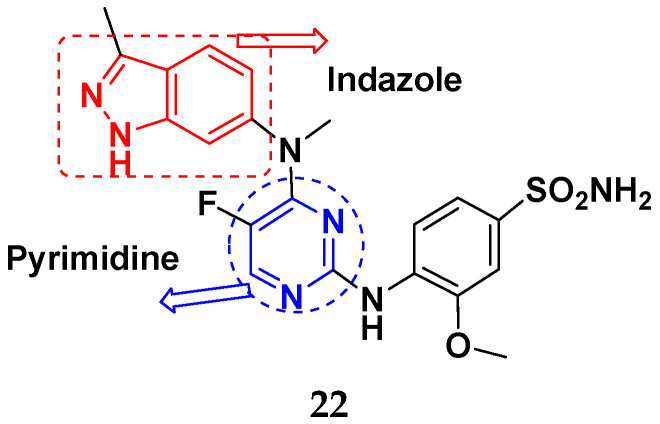
A pyrimidine-indazole based hybrid **22**.

Boloor et al. (2012) synthesized and patented pyrimidine-indazole based hybrids as VEGFR2 inhibitors. Compound **22** showed potent activity with an IC_50_ < 50 μM. Furthermore, the lead compound **22** (Figure 26) was evaluated in an endothilian cell proliferation assay where it displayed activity withIC_50s_ of 1-200 nM [73].

**Figure 27:**
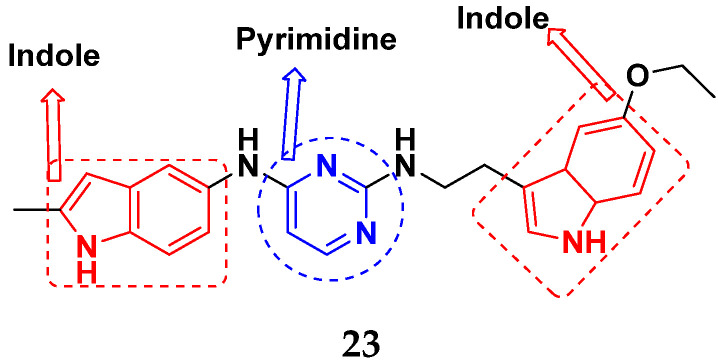
A pyrimidine-di-indazole based hybrid (**23**).

Hogberg et al. (2012) developed and patented indole based pyrimidine hybrids and evaluated their anticancer activity as tubulin inhibitors. Among the synthesized compounds, compound **23** (Figure 27) showed potent activity on CCRFCEM (T lymphoblastoid cells) with an EC_50_ value of 0.015 µM [74]. 

**Figure 28:**
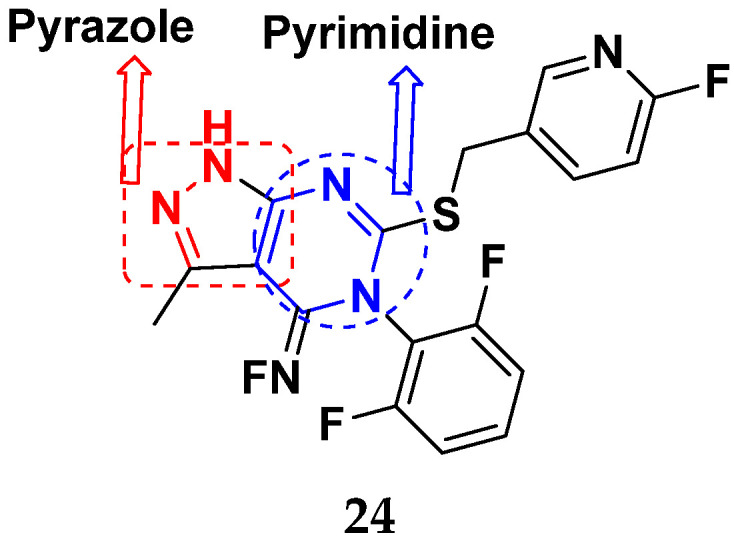
A pyrimidine hybrid (**24**).

Mao et al. (2012) synthesized and patented pyrimidine-pyrazole based hybrid molecules and evaluated their anticancer activity against H1993 cells. The synthesized compounds did not show desirable activity on representative cell line. Furthermore, potent compound **24** (Figure 28) was subjected to enzymatic assay on cMet protein and displayed excellent activity, having an IC_50_ in the nM range [75]. 

**Figure 29:**
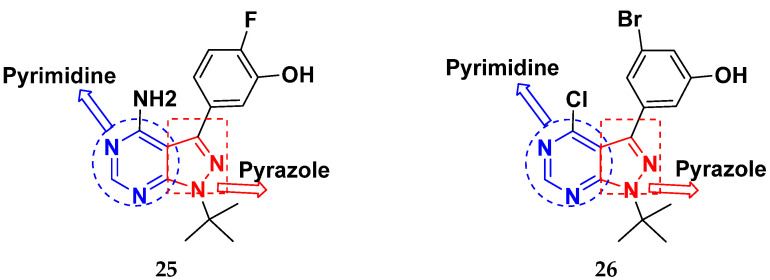
Pyrimidine-pyrazole based hybrid (**25** and **26**).

Tanaka et al. (2012) synthesized and patented pyrimidine-pyrazole based hybrid molecules and evaluated their anticancer activity as Fyn inhibitors. Among the synthesized compounds, compound **25** (Figure 29) showed excellent activity with an IC_50_ of 3 nM. Furthermore, **26** (Figure 29) showed excellent activity on carbonyl reductase 1 with an IC_50_ of 28 nM [76].

**Figure 30:**
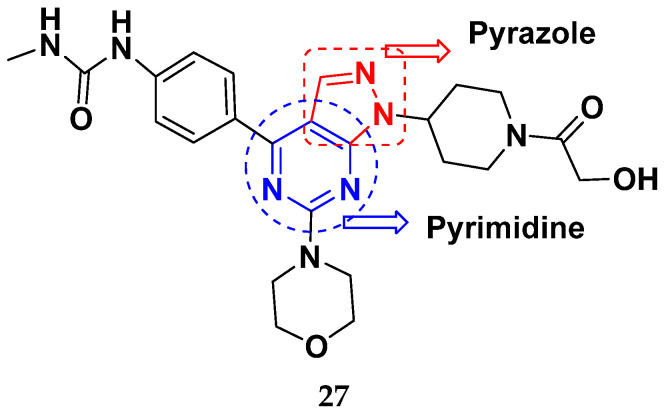
A Pyrimidine-pyrazole based hybrid (**27**).

Liang et al. (2013) synthesized and patented pyrimidine-pyrazole based hybrid molecules, and evaluated their anticancer activity as mammalian target of rapamycin mTOR inhibitors. Compound **27** (Figure 30) showed potent activity on A549 and U-87MG (glioma cells) with an IC_50_ < 1 µM [77]. 

**Figure 31:**
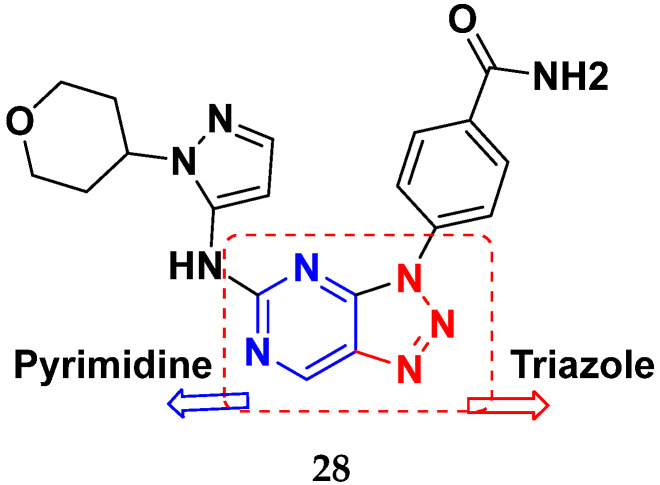
A pyrimidine-triazole based hybrid (**28**).

Dorsch et al. (2013) synthesized and patented pyrimidine-triazole based hybrids and evaluated their anticancer activity as general control non-derepressible 2 (GCN2) inhibitors of U20S human cells. Among the synthesized hybrids, the compound **28** (Figure 31) showed excellent activity with IC_50_ ≤ 0.3µM [78].

**Figure 32:**
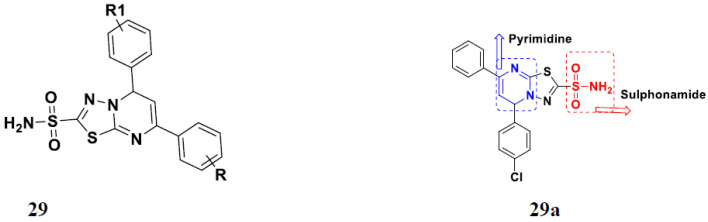
Structure of sulfonamide-thiazole fused pyrimidine hybrids and the most promising compound **29a**.

El-Sayed et al. (2011) synthesized sulfonamide-thiazole fused pyrimidine derivatives, and evaluated their anticancer activity in amethyl green/DNA displacement assay. Among the synthesized compounds, compound **29a** showed potent activity with an IC_50_ of 40 µg/mL and high DNA binding affinity. Furthermore, in vivo studies showed that the compound **29a** increased the % lifespan of mice by 42.86% over standard fluorouracil. The structure of sulfonamide-thiazole fused pyrimidine derivatives is shown in Figure 32 and the in vitro cytotoxicity of compounds **29**(**b**–**e**) against human cancer cell lines is shown in Table 22 [79].

**Figure 33:**
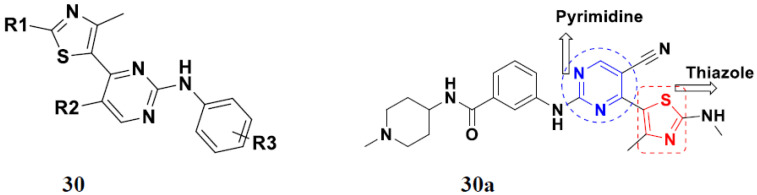
Structure of tri-substituted pyrimidine hybrids and the most promising compound **30a**.

Shao et al. (2013) synthesized tri-substituted pyrimidine based hybrid derivatives, and evaluated their anticancer activity as cyclin-dependent kinase (CDK) inhibitors. Most of the synthesized compounds showed potent anticancer activity. Among them, compound **30a** showed potent kinase inhibition against CDK9T1, CDK1B, CDK2A, CDK7H withK_i_ of 14, 262, 316, and 163 nM, and cell toxicity activity against HCT-116, and MCF-7 cell lines with IC_50_ value of 0.79, and 0.64 µM, respectively. Furthermore, compound **30a** showed potent activity of Mal-1 and caspase-3 inhibition by down regulation of apoptotic protein. The structure of substituted pyrimidine derivatives is shown in Figure 33 and the in vitro cytotoxicity of compounds **30**(**b**–**e**) against human cancer cell lines is shown in Table 23 [80].

**Figure 34:**
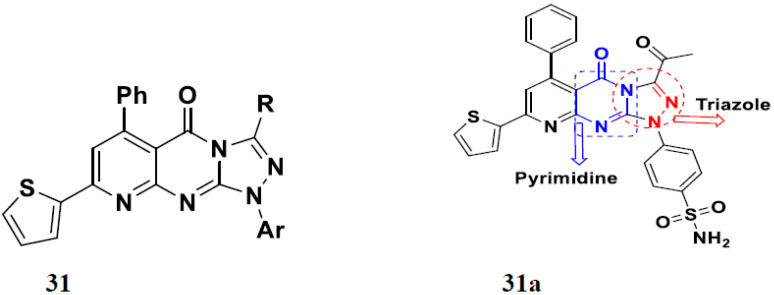
Structure of a pyrimidine-triazole based hybrids and the most promising compound **31a**.

Fares et al. (2014) synthesized pyrimidine-based triazole hybrid derivatives and evaluated their anticancer activity. Most of the synthesized compounds showed potent anticancer activity. Among them, compound **31**(**a**) showed excellent activity against various cell lines including MCF-7, human hepatoma carcinoma cells (HEPG2), A-549, and (PC-3) with IC_50_ of 37.96, 56.65, 0.41, and 0.36 µM. Furthermore, compound **31a** was screened in a CDK4 and CDK6 inhibition assay and compound showed 0 and 2% inhibition at 1 µM, 5 and 1% inhibition at 10 µM and 21 and 17% inhibition at 100 µM with control staurosporine having 93 and 90% inhibition at 1 µM. The structure of pyrimidine-triazole based hybrid derivatives is shown in Figure 34 and the in vitro cytotoxicity (µM) of compounds **31**(**b**–**e**) against human cancer cell lines is shown in Table 24 [81].

**Figure 35:**
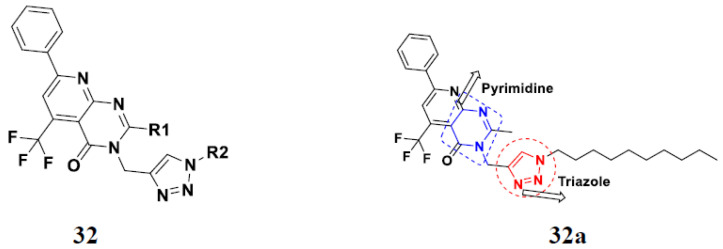
Structure of pyrimidine-triazole based hybrids and the most promising compound **32a**.

Kurumurthy et al. (2019) synthesized pyrimidine based triazole hybrids and evaluated their anticancer activity. Most of the synthesized compounds showed potent anticancer activity. Among them, compound **32**(**a**) showed excellent activity against various cell lines including U_937_, THP-1 and Colo205 cells having IC_50_ values of 6.20, 11.27, and 15.01µg/mL with control etoposide having IC_50_ values of 17.94, 2.16, 7.24, and 1.26 µg/mL, respectively. The structure of pyrimidine-triazole based hybrid derivatives is shown in Figure 35 and the in vitro cytotoxicity of compounds **32**(**b**–**e**) against human cancer cell lines is shown in Table 25 [82]. 

**Figure 36:**
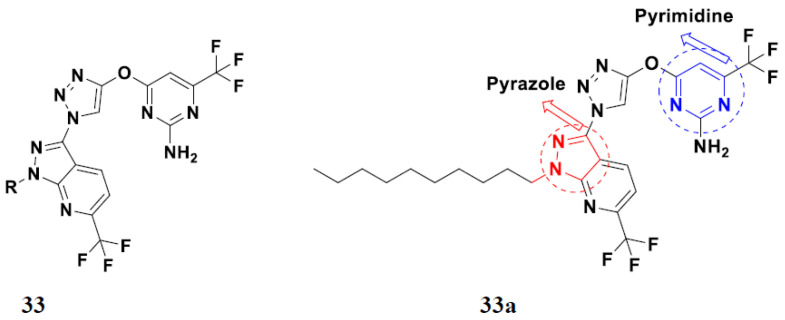
Structure of pyrimidine-pyrazole based hybrids and the most promising compound **33a**.

Nagendra et al. (2014) synthesized pyrimidine based pyrazole hybrids and evaluated their anticancer activity. Most of the synthesized compounds showed potent anticancer activity. Among them, compound **33**(**a**) showed excellent activity against various cell lines including A549, MCF7, DU145 (prostate cancer cell) and HeLa with IC_50_ values of 4.2, 37.2, 5.8, and 34.3 µM and control 5-FU having IC_50_ values of 1.3, 1.4, 1.5, and 1.3 µM, respectively. The structure of pyrimidine-pyrazole based hybrid derivatives is shown in Figure 36 and the in vitro cytotoxicity (µM) of compounds **33**(**b**–**e**) against human cancer cell lines is shown in Table 26 [83]. 

**Figure 37:**
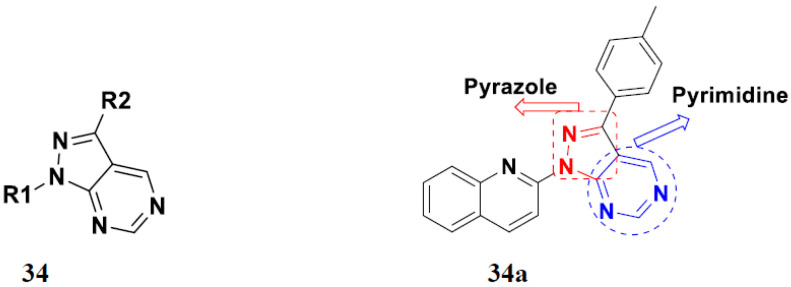
Structure of pyrimidine-pyrazole based hybrids and the most promising compound **34a**.

Huang et al. (2012) synthesized pyrimidine based pyrazole hybrids and evaluated their anticancer activity. Most of the synthesized compounds showed potent anticancer activity. Among them, compound **34**(**a**) showed excellent activity against various cell lines including NCI-H226 (human lung squamous carcinoma cells), NPC-TW01 (nasopharyngeal carcinoma), and Jurkat having GI_50_ 29, 30, and 54 µM, respectively. The structure of pyrimidine-pyrazole based hybrid derivatives is shown in Figure 37, and the in vitro cytotoxicity of compounds **34**(**b**–**e**) against human cancer cell lines is shown in Table 27 [84]. 

#### FDA Approved Pyrimidine Based Hybrids

**Figure 38:**
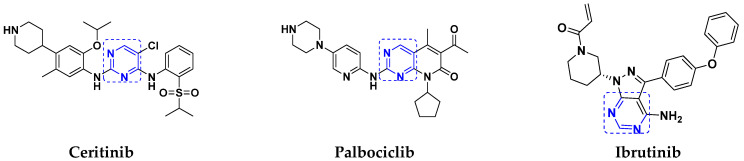
Pyrimidine based FDA approved drugs.

Ceritinib, palbociclib, and ibrutinib are three hybrids which contain pyrimidine scaffold that have received US-FDA approval for their antitumor effects against non-small cell lung cancer, advanced breast cancer and leukemia, respectively. Pyrimidine-based FDA approved drugs are depicted in Figure 38 and their details are shown in Table 28.

### 3.6. Quinoline-Based Hybrids

Quinoline has been identified as a major scaffold with huge therapeutic potential, including antibacterial, anti-viral, anti-helmintic, anti-malarial and anti-prozoal activities. Due to its derivatives having demonstrated outstanding effects against cancerous cells via various mechanisms, the quinoline nucleus has played a crucial role in the research and development of chemotherapeutic agents. Camptothecin is a natural anticancer agent with the capacity to obstruct DNA topoisomerase [89,90,91].

**Figure 39:**
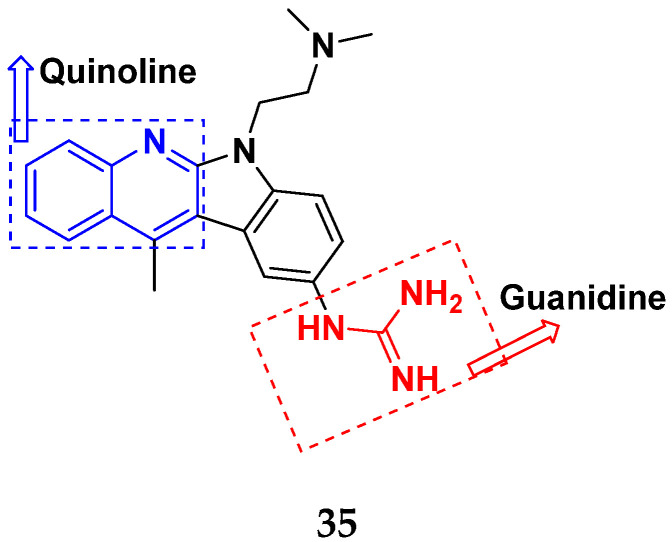
Structure of quinoline-guanidine based hybrid **35**.

Sidoryk et al. (2015), synthesized quinoline based guanidine hybrids and evaluated their anticancer activity. Most of the synthesized compounds showed potent anticancer activity. Among them, compound **35** showed excellent activity against BALB/3T3, A549, MCF-7, LoVo, and KB cell lines (Table 29). Furthermore, compound **35** (Figure 39) showed excellent activity in a DNA displacement assay and potent activity in G2/M phase of the cell cycle [92].

**Figure 40:**
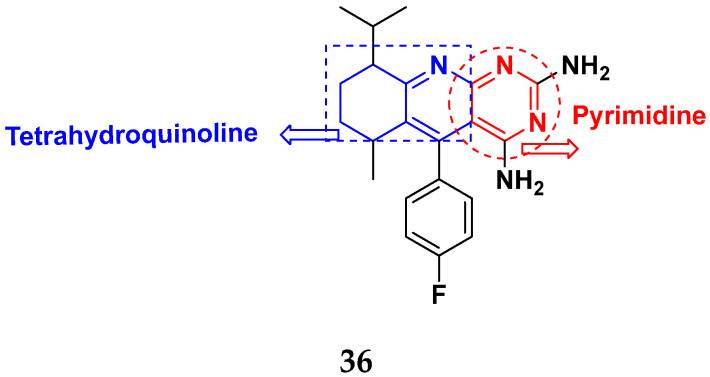
Structure of tetrahydro-pyrimido-quinoline based hybrid **36**.

Gedawy et al. (2015) synthesized tetrahydro-pyrimido-quinoline based hybrids and evaluated their anticancer activity. Most of the synthesized compounds showed potent anticancer activity. Among them, compound **36** (Figure 40) showed excellent activity against HCT-116, and MCF-7 (Table 30) [93].

**Figure 41:**
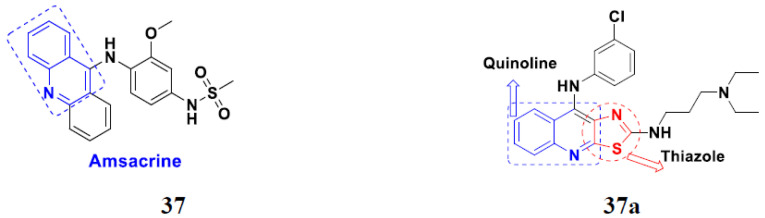
Structure of quinoline hybrid amsacrine **37** and the most promising compound **37a**.

Sanchez et al. (2011) synthesized quinoline-based thiazole hybrids based on the structure of m-Amsacrine (**37**, Figure 41), and evaluated their anticancer activity against cancerous (K-562) and non-cancerous (PMBCs) cells. Compound **37a** showed excellent activity with IC_50_ values of 28.7 and 7.82 µM, respectively, compared to a control of Paclitaxel (IC_50_ 0.25 µM). Mechanistically, compound **36**(**a**) induced appototic cell death via caspase activation with an IC_50_ of 7.8 µM [94]. 

**Figure 42:**
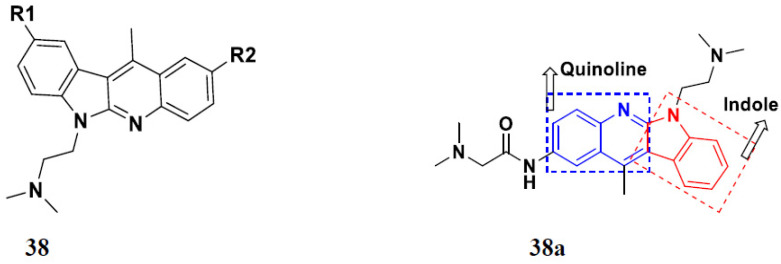
Structure of quinoline-indole based hybrids and the most promising compound **38a**.

Luniewski et al. (2012) synthesized quinoline based indole hybrids and evaluated their anticancer activity. Most of the synthesized compounds showed potent anticancer activity. Among them, compound **38**(**a**) showed excellent activity against KB, A-549, MCF-7, Hs294T, and BALB/3T3 cell lines having IC_50_ values of 0.15, 0.24, 0.38, 0.62, and 0.31 µM with control 5,11-dimethyl-5H-indolo [2,3-b]quinoline (DIMIQ) at IC_50_ values 1.14, 2.19, 1.50, 9.70, and 5.70 µM, respectively. Furthermore, compound **38a** showed excellent topo II inhibitory activity at concentration of 0.05 µM with control DIMIQ (Conc. 0.5 µM), m-AMSA (Conc. 0.05 µM), and daunorubicind (Conc. 0.5 µM) and cell cycle inhibitory activity at a concentration of 0.10 µM with control DIMIQ (Conc. 1.02 µM). Additionally, it showed positive inhibitory results in the G2M phase of the cell cycle. The structure of quinoline-indole based hybrid derivatives is given in Figure 42 and the in vitro cytotoxicity of compounds **38**(**b**–**e**) against human cancer cell lines is shown in Table 31 [95].

**Figure 43:**
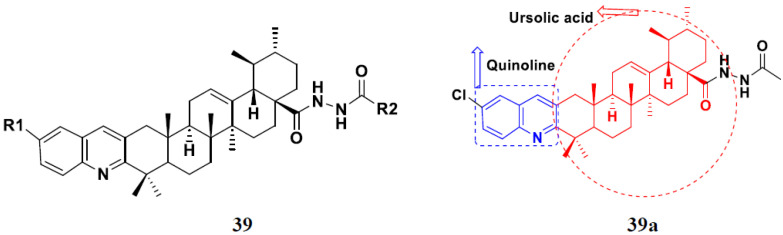
Structure of quinoline based ursolic acid hybrids and the most promising compound **39a**.

Jin et al. (2019) synthesized quinoline-based ursolic acid hybrids and evaluated their anticancer activity. Most of the synthesized compounds showed potent anticancer activity. Among them, compound **39**(**a**) showed excellent activity against MDA-MB-231, HeLa, SMMC-7721, and QSG-7701 (hepatocyte *cell* line) at IC_50_ 0.12, 0.08, 0.34, and 10.76 µM with control Etoposide with IC_50_ values of 5.26, 2.98, 3.48, and 28.75 µM, respectively. Furthermore, compound **39**(**a**) showed excellent activity apoptosis-inducing activity in on HeLa cell lines (for 48 h). The structure of quinolinebased ursolic acid hybrids derivatives is shown in Figure 43, and the in vitro cytotoxicity (µM) of compounds **39**(**b**–**e**) against human cancer cell lines is shown in Table 32 [96].

**Figure 44:**
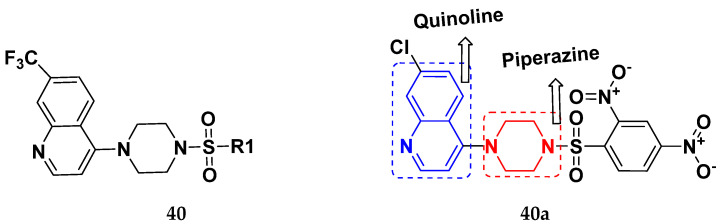
Structure of quinolone-based piperazine hybrids and the most promising compound **40a**.

Solomon et al. (2019) synthesized quinoline based sulphonamide/piperazine hybrids and evaluated their anticancer activity. Most of the synthesized compounds showed potent anticancer activity. Among them, compound **40**(**a**) showed excellent activity against MB231, MB468, MCF7, 184B5 (normal mammary tissue), and MCF10A cell lines, having GI_50_ values of 3.4, 0.7, 2.3, 9.0, and 12.3 µM. Control chloroquine, had GI_50_ values of 22.5, 28.6, 38.4, 76.1, and 81.26 µM and Cisplatin having GI_50_ value 23.7, 31.0, 25.8, 25.5, and 51.51µM, respectively. Furthermore, compound **40**(**a**) showed cell cycle interruption at the meta phase, and also increased the liposamal volume in cancerous cells which led to cell death. The structure of quinoline based piperazine hybrids is given in Figure 44 and the in vitro cytotoxicity (µM) of compounds **40**(**b**–**e**) against human cancer cell lines is shown in Table 33 [97].

**Figure 45:**
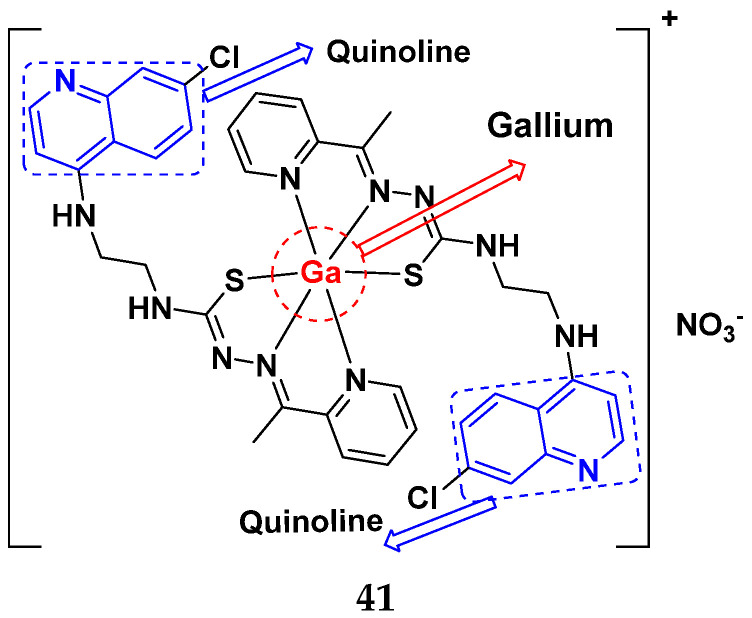
Quinoline-based gallium(III) hybrid (**41**).

Kumar et al. (2014) synthesized quinoline-based gallium(III) hybrids and evaluated their anticancer activity. Synthesized hybrid **41** showed potent activity against HCT-116, Caco-2 (human colon cancer cell), HT-29, and CCD-18C (colonic fibroblasts), having IC_50_ values of 14.26, 19.56, 19.66, and 28.28 µM, respectively. Control etoposide had comparative values of 38.10, 32.90, 35.10, and 58.90 µM, respectively. Furthermore, hybrid **41** was evaluated for anti-malarial activity, showing more potent activity than lumefantrine on the 3D7 strain of Plasmodium falciparum. A quinoline-based gallium(III) hybrid is depicted in Figure 45 [98].

#### Quinoline-Based FDA Approved Drugs

**Figure 46:**
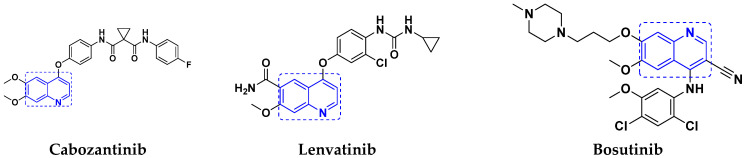
FDA approved drugs with quinoline hybrids.

Quinoline containing drugs that have received FDA approval include lenvatinib, cabozantinib, and bosutinib which are protein kinase inhibitors and are used to cure medullary thyroid cancer and chronic myelogenous leukemia accordingly. FDA approved drugs with quinoline hybrids are depicted in Figure 46 and their details are shown in Table 34.

### 3.7. Quinone Hybrids

Quinones are found in all living beings; particularly animals, plant and microbes. Through serving as crucial links in the cell nucleus respiratory cycle, they play a vital role in the power generation of such species. Numerous distinct medicinal uses of quinones have been noted, such as antiviral, antithrombotic, antiplatelet, antibacterial, antifungal, anti-inflammatory, and antiallergic properties [101,102].

**Figure 47:**
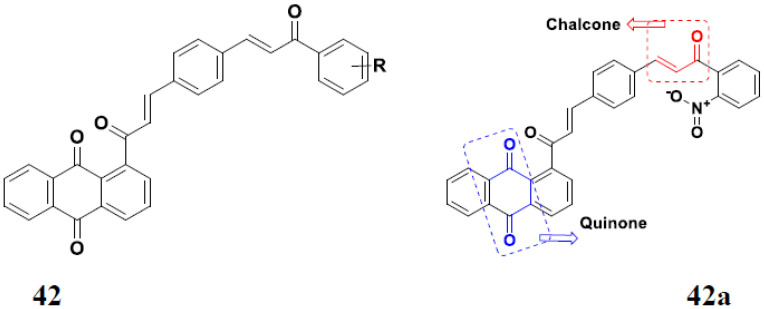
Structure of quinone-based chalcone hybrids and the most promising compound **42a**.

Markovic et al. (2015) synthesized quinone based chalcone hybrids and evaluated their anticancer activity. Most of the synthesized compounds showed potent anticancer activity. Among them, compound **42**(**a**) showed excellent activity against HeLa, LS174 (colorectal cancer cells), A549, and MRC-5 (multipotent stem cells) with IC_50_ values of 2.73, 6.44, 28.84, and 48.76, respectively. Furthermore, compound **42**(**a**) showed caspase based apoptosis in G2/M and S phase of cell division. The structure of quinone-based chalcone hybrids derivatives is given in Figure 47 and the in vitro cytotoxicity of compounds **42**(**b**–**e**) against human cancer cell lines is shown in Table 35 [103].

**Figure 48:**
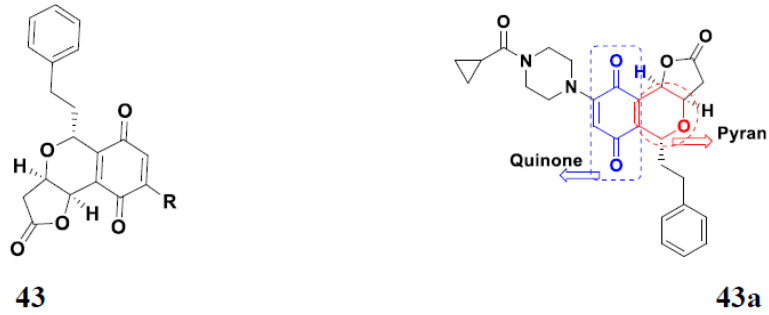
Structure of quinone based pyran hybrids and the most promising compound **43a**.

Jiang et al. (2015) synthesized quinone based pyran hybrids and evaluated their anticancer activity. Most of the synthesized compounds showed potent anticancer activity. Among them, compound **43**(**a**) showed excellent activity against KB (throat cancer cells), KB/VCR (colon cancer cells), A549, and HL60, with IC_50_ values 4.05, 1.28, 0.62, and 1.73 µg/mL. A control vincristine had IC_50_ values of 0.46, 0.26, and 12.09 µg/mL (for KB, KB/VCR, A549), respectively, and adriamycin had an IC_50_ value 0.02 µg/mL (HL60). The structure of quinone-based pyran hybrid derivatives is given in Figure 48 and the in vitro cytotoxicity (µg/mL) of compounds **43**(**b**–**e**) against human cancer cell lines is shown in Table 36 [104].

#### Quinone Containing FDA Approved Drugs

**Figure 49:**
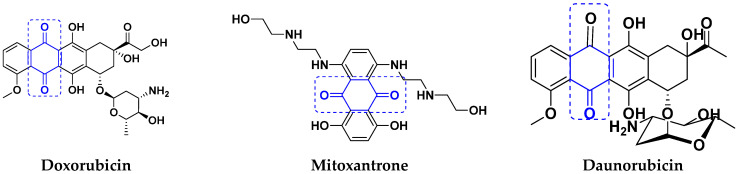
FDA approved drugs with Quinone hybrids.

Doxorubicin, daunorubicin, and mitoxantrone drugs are used to treat several types of cancer. These drugs are shown in Figure 49 [101].

### 3.8. Imidazole Based Hybrids

The imidazole derivatives have recently gained a lot of interest and are already present in many existing treatments. Imidazole was first synthesized in 1858 using glyoxal and formaldehyde by Heinrich Debus [105]. Various medicinal properties of imidazole based hybrid molecules have been reported; especially as antitumor [106], anti-diabetic, anti-HIV, anti-protozoal, anti-mycobacterial, anti-inflammatory, analgesic, and anti-protozoal agents [107,108].

**Figure 50:**
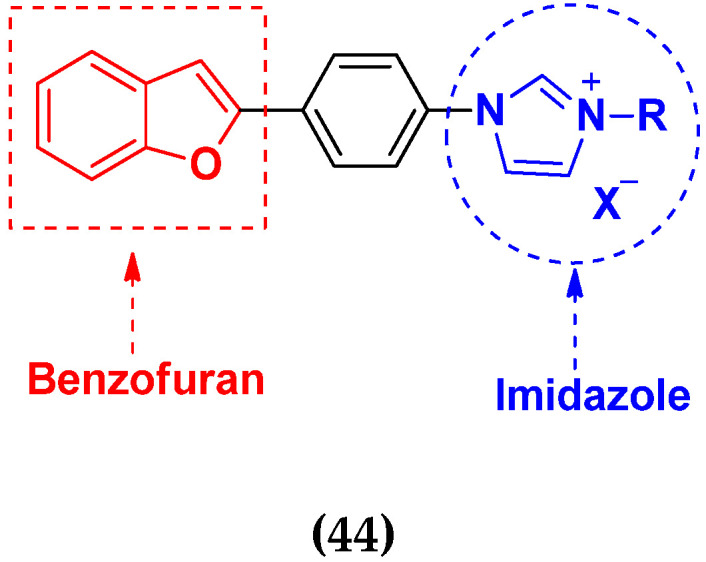
Structure of imidazole-based benzofuran hybrid derivatives (**44**).

Xiao-Dong Yang et al. (2012) synthesized different derivatives of novel hybrid compounds between 2-phenylbenzofuran and imidazole. Myeloid liver carcinoma (SMMC-7721), colon carcinoma (SW480), breast carcinoma (MCF-7), lung carcinoma (A549) and leukemia (HL-60) cell lines were used for in vitro study of the cytotoxic effects of synthesized hybrids with cisplatin (DPP) as the standard drug. Five derivatives showed more potent cytotoxic activity than standard DPP. The structure of imidazole based benzofuran hybrid derivatives are given in Figure 50 and the in vitro cytotoxic activities of hybrid compounds **44**(**a**–**e**) (IC_50_, µM) are listed in Table 37 [109].

**Figure 51:**
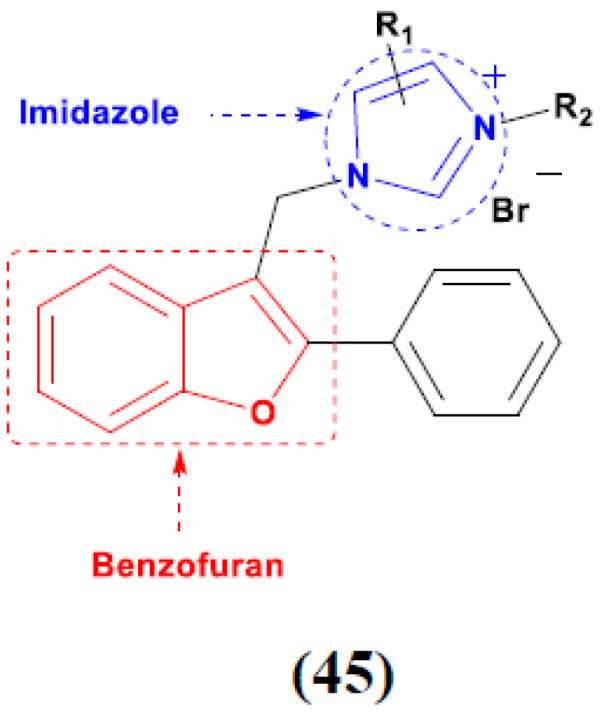
Structure of imidazole based benzofuran hybrid derivatives (**45**).

Wen Chen et al. (2013) synthesized numerous derivatives of novel hybrid **2**-phenyl-3-alkylbenzofuran and imidazole compounds. Myeloid liver carcinoma (SMMC-7721), colon carcinoma (SW480), breast carcinoma (MCF-7), lung carcinoma (A549) and leukemia (HL-60) cell lines were used for in vitro cytotoxic testing of synthesized hybrids, with DPP as the standard drug. They found that five derivatives showed more potent cytotoxic activity than standard DPP. The structure of imidazole based benzofuran hybrid derivatives is given in Figure 51 and the in vitro cytotoxic activities of hybrid compounds **45**(**a**–**e**) (IC_50_, µM) are listed in Table 38 [110].

**Figure 52:**
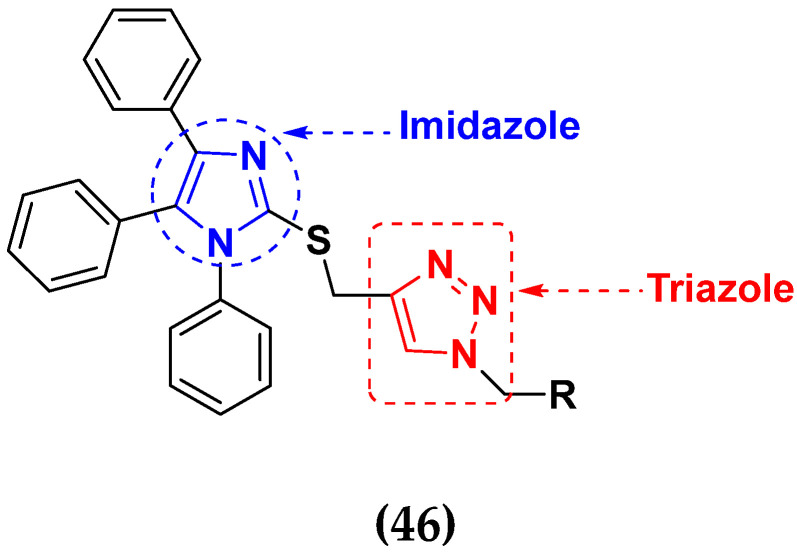
Structure of imidazole based triazole hybrid derivatives (**46**).

Al-blewi et al. (2013) synthesized numerous derivatives of novel imidazole-1,2,3-triazole hybrids. The synthesized compounds were screened for their anticancer activity against three different types of cancer, namely human colon carcinoma (Caco2 and HCT116), human cervical carcinoma (HeLa) and human breast adenocarcinoma (MCF-7) cancer cells-, using doxorubicin as a standard drug. They found that these derivatives showed potent more cytotoxic activity than standard doxorubicin. The structure of imidazole based triazole hybrid derivatives is given in Figure 52 and in vitro cytotoxic activities of hybrid compounds **46**(**a**–**e**) are listed in Table 39 [111].

**Figure 53:**
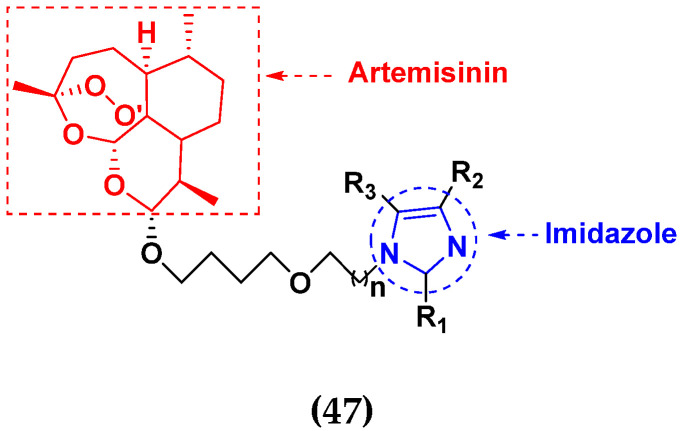
Structure of imidazole-based artemisinin hybrid derivatives (**47**).

Yanping Hu et al. (2019) designed and synthesized a series of artemisinin-imidazole hybrids derivatives with multidrug resistance (MDR) reversal activity. All hybrids were screened in vitro for anticancer activities against four human cancer cell lines, human breast cancer (MCF-7), human non-small-cell lung (A549), (HEPG-2), breast cancer (MDA-MB-231) and normal human hepatic cells (L02). Adriamycin was used as reference drug. They found that most of the synthesized compounds showed higher anticancer activities than artemisinin. The structure of imidazole based artemisinin hybrid derivatives is given in Figure 53 and the in vitro cytotoxic activities of hybrid compounds **47**(**a**–**e**) are listed in Table 40 [112].

**Figure 54:**
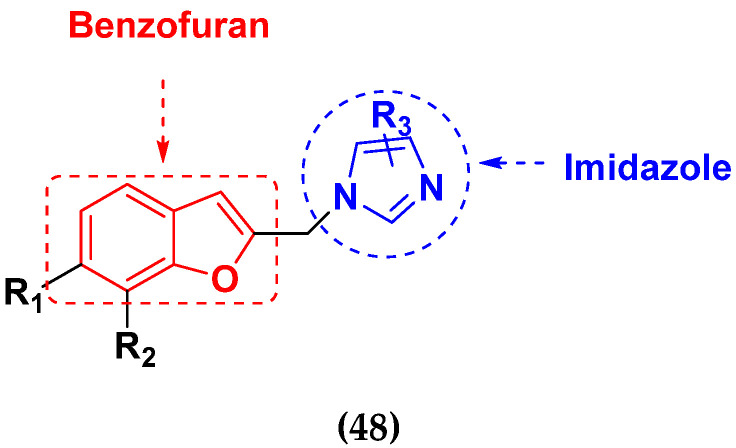
Structure of imidazole based benzofuran hybrid derivative **48**.

Wen-Jian Song et al. (2012) designed and synthesized a series of novel hybrid compounds of 2-substituted benzofuran and imidazole. In vitro anticancer activity of synthesized hybrids against a panel of human tumor cell lines i.e., ovarian carcinoma cell line (Skov-3), leukemia (HL-60) and breast carcinoma (MCF-7) was evaluated. They found that the hybrid compounds were more selective than standard DPP. The structure of imidazole based benzofuran hybrid derivatives is given in Figure 54 and the in vitro cytotoxic activities of hybrid compounds **48**(**a**–**e**) are listed in Table 41 [113].

#### Imidazole Based Hybrids That Are FDA Approved or under clinical Trial

**Figure 55:**
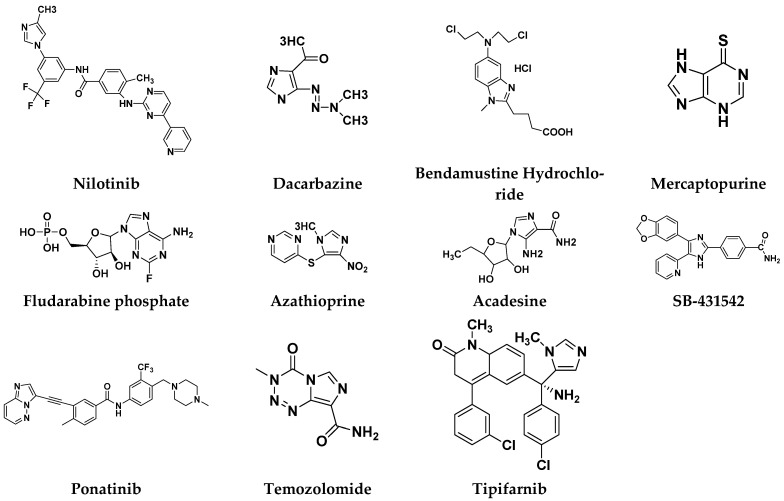
Imidazole based anticancer drugs that are FDA approved or in clinical trials.

Different imidazole-based anticancer hybrid drugs that are FDA approved/or under clinical trials are shown in (Figure 55), and their chemical structures brand/company name and targets are in Table 42.

### 3.9. Selenium-Based Hybrids

Selenium (Se) is a unique trace element. Se directly or indirectly exerts antioxidant functions in the human body. However, during recent years, researchers reported that Se containing compounds exhibit superior anticancer effects, with high efficacy and selectivity [117]. It has been reported that a variety of organic selenium compounds, including selenoesters, selenocyanates, methylseleninic acid, isoselenocyanates, diselenides, and endocyclic selenium, have anticancer properties [118].

Organic Se compounds have lower systemic effects, fewer side effects, and strong anti-tumor action. They also have a greater ability to inhibit metastasis. Several novel organic Se compounds have been synthesized in order to further improve the selectivity, specificity and efficacy and to lower the toxicity [119].

**Figure 56:**
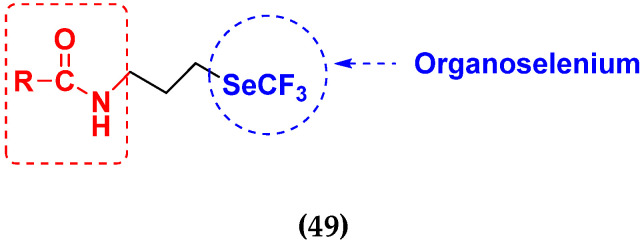
Organoselenium hybrid derivatives (**49**).

Xianran He et al. (2020) synthesized organoselenium (SeCF_3_) derivatives as potential anticancer agents. The anticancer activity of the synthesized compounds was assessed using human cancer cell lines, human colon adenocarcinoma cells (SW480), human cervical cancer cells (HeLa), human lung carcinoma cells (A549), human breast adenocarcinoma cells (MCF-7). The in vitro biological evaluation was conducted at 24, 48 and 72 h intervals, and it was found that the organoselenium hybrid compounds were more selective than standard Fluorouracil (5FU). The structure of organoselenium hybrid derivatives is given in Figure 56 and the in vitro cytotoxic activities of hybrid compounds **49**(**a**–**c**) are listed in Table 43 [118].

**Figure 57:**
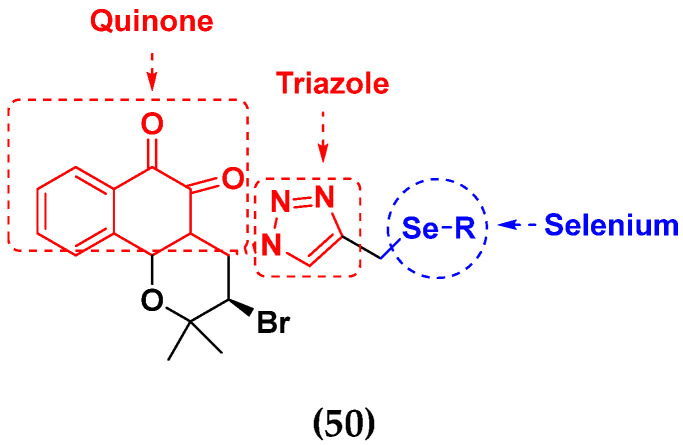
Structure of selenium-based quinone triazole hybrid derivatives (**50**).

Guilherme A. Jardim et al. (2017) synthesized selenium-containing quinone-based 1,2,3-triazoles with potential antitumor activity via rhodium-catalyzed C-H bond activation and click reactions. All compounds were evaluated against five types of cancer cell lines: human promyelocytic leukemia cells (HL-60), human colon carcinoma cells (HCT-116), human glioblastoma cells (SF295), human lung cells (NCIH-460) and human prostate cancer cells (PC3), using paclitaxel as a positive control. L929 cells were also used to test the cytotoxic potential of the naphthoquinoidal derivatives in non-tumor cells. Overall, these compounds represented promising new lead derivatives with potential antitumor activity. The structure of selenium based quinone triazole hybrids is given in Figure 57 and in vitro cytotoxic activities of hybrid compounds **50**(**a**–**e**) are listed in Table 44 [120].

**Figure 58:**
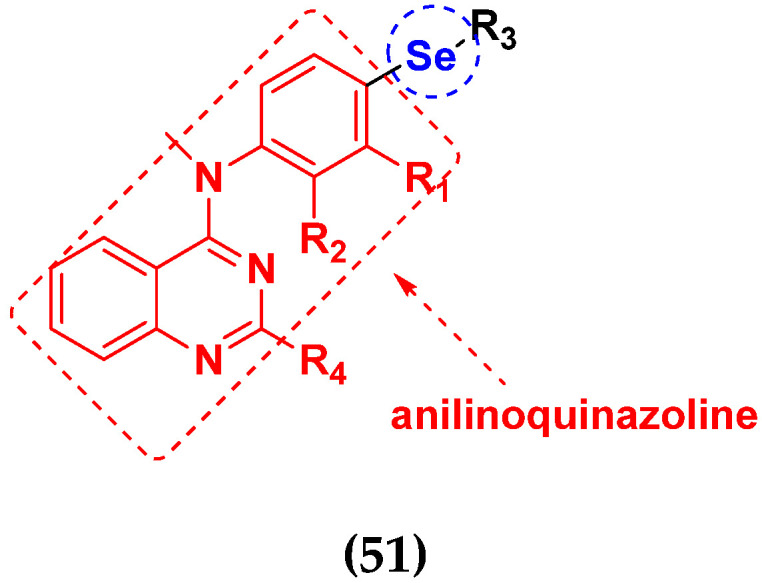
Structure of selenium based anilino quinazoline hybrid derivatives (**51**).

An B et al. (2018) synthesized selenium-containing 4-anilinoquinazoline hybrids and evaluated them as tubulin polymerization inhibitors. All the synthesized compounds were screened against a panel of six human tumor cell lines, human colon cancer cells (RKO), HEPG2, breast adenocarcinoma (MCF-7), human epithelial cervical cancer cells (HeLa), human colon cancer cells (HCT116), and human gastric cancer cells (MGC803). An antiproliferative activity assay showed that most of compounds inhibited human cancer cells at low nano-molar concentrations. These compounds disturbed microtubule dynamics, lowered mitochondrial membrane potential, and stopped Hela cells in the G2/M phase, ultimately leading to apoptosis. The structure of selenium based anilino quinazoline hybrids is given in Figure 58 and the in vitro cytotoxic activities of hybrid compounds **51**(**a**–**e**) are listed in Table 45 [121].

**Figure 59:**
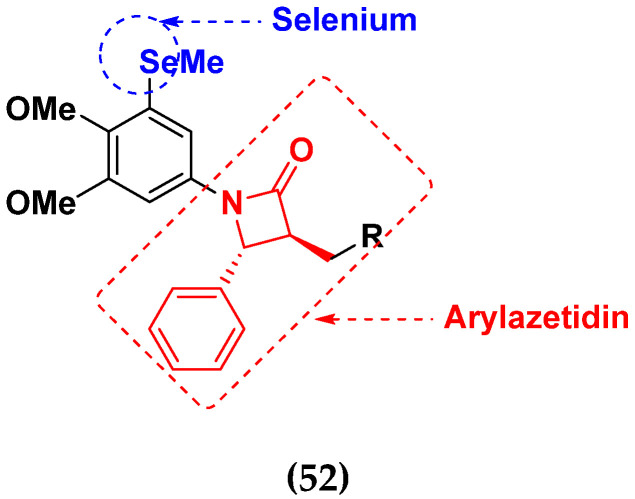
Structure of selenium based anilino quinazoline hybrid derivatives (**52**).

Hairong Tang et al. (2021) synthesized novel selenium-containing chiral 1,4-diarylazetidin-2-ones and biologically evaluated for antitumor activities. All the newly synthesized selenium-containing compounds were screened for their antiproliferative activity against four human cancer cell lines, human epithelial cervical cancer cells (HeLa), human hepatoma cells (HUH-7), ovarian carcinoma cells (Skov-3) and human ovarian cancer cells (A2780), using paclitaxel as the positive control. The structure of Selenium based arylazetidin hybrids is shown in Figure 59 and the in vitro cytotoxic activities of hybrid compounds **51**(**a**–**d**) are listed in Table 46 [122].

**Figure 60:**
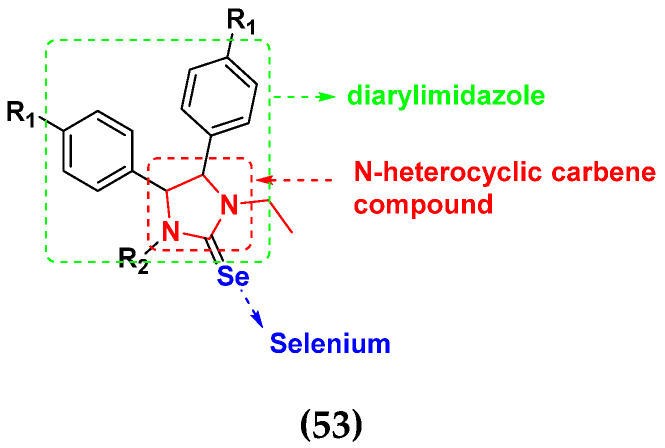
Structure of selenium-based diaryl imidazole hybrid derivatives (**53**).

Sheng Huang et al. (2021) designed and synthesized fourteen novel selenium N-heterocyclic carbene (Se-NHC) compounds derived from 4,5-diarylimidazole and evaluated their antiproliferative activity towards ovarian cancer cells (A2780) and normal ovarian epithelial cells (IOSE80). Most of them were more effective towards ovarian cancer cells (A2780) than HepG2 hepatocellular carcinoma (HCC) cells. In addition, compound **53** displayed more than two-fold higher cytotoxicity to A2780 cells than to normal ovarian epithelial cells (IOSE80). Further research demonstrated that these inhibitors generated reactive oxygen species (ROS), harmed mitochondrial membrane potential (MMP), stopped cells from entering G0/G1 phase, and ultimately promoted apoptosis in the A2780 cells. The structure of selenium-based diarylimidazole hybrids is shown in Figure 60 and the in vitro cytotoxic activities of hybrid compounds **53**(**a**–**e**) are listed in Table 47 [123].

#### Selenium-Based Hybrids That Are FDA Approved or under Clinical Trials

**Figure 61:**
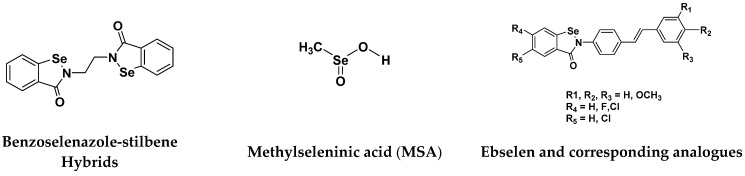
Selenium based potent anticancer compounds.

Different selenium-based potent anticancer drugs their chemical structure (Figure 61), mode of action and doses are shown in Table 48.

### 3.10. Platinum-Based Hybrids

Platinum (Pt) medicines are still among the most often used anticancer treatments after more than 40 years of use. It is not unexpected that new research into changes in DNA repair pathways provides a reasonable explanation for Pt medicines’ efficacy since they primarily target DNA [124]. The first platinum drug, cisplatin, was discovered by Barnett Rosenberg in 1960 [125] and received FDA approval in 1978 for the treatment of advanced ovarian, bladder and testicular cancer [126]. Oxaliplatin and carboplatin are also platinum containing clinical drugs. Despite the widespread use of platinum medicines in cancer treatment regimens, there are several associated drawbacks. It is associated with severe side effects such as nephrotoxicity, neurotoxicity, and ototoxicity [127]. Additionally, using platinum medications has a number of adverse effects that range in intensity from mild to toxic (at high doses). In an attempt to circumvent these problems, a large number of platinum complexes have been prepared and tested for anticancer activity [128].

**Figure 62:**
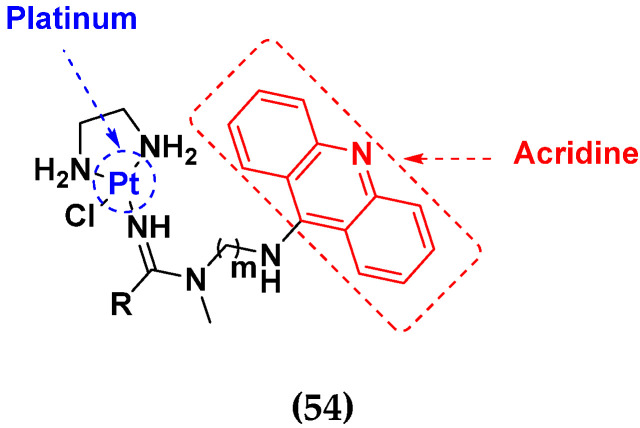
Structure of Platinum−acridine hybrid derivatives (**54**).

A. Graham et al. (2012) synthesized platinum−acridine hybrid anticancer agents. In vitro cytotoxicity activity was evaluated on different cell lines, ovarian cancer (OVCAR-3), breast cancer (MCF-7, MDA-MB231), pancreatic (PANC1) and non-small cell lung cancer cells (NCI-H460) using cisplatin as the positive control. The structure of Platinum−acridine hybrids is given in Figure 62 and in vitro cytotoxic activities of hybrid compounds **54**(**a**–**e**) are listed in Table 49 [129].

**Figure 63:**
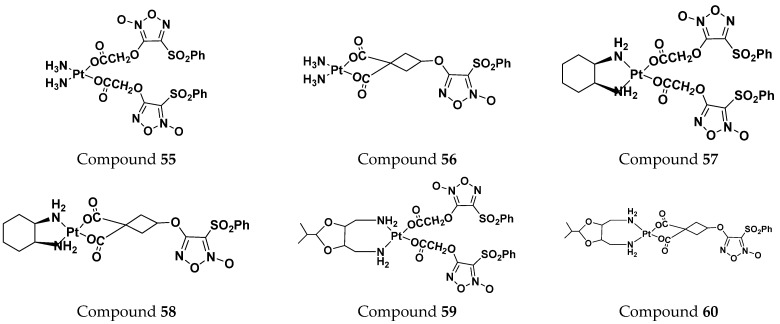
Platinum hybrid derivatives (**55**–**60**).

Jian Zhao et al. (2012) designed and synthesized six novel platinum (II) complexes 1−6 bearing different furoxan moieties as nitric oxide (NO) donors. The furoxan groups were introduced to the platinum complexes to release NO, which may have synergisticaction with the platinum-based moieties on the tumor cells. It was found that all compounds exhibited higher cytotoxicity against human cancer cell lines HCT-116 and SGC-7901 compared to standard carboplatin, and oxaliplatin. The structure of platinum hybrids is shown in Figure 63 and the in vitro cytotoxic activities of hybrid compounds (**55**–**60**) are listed in Table 50 [130].

**Figure 64:**
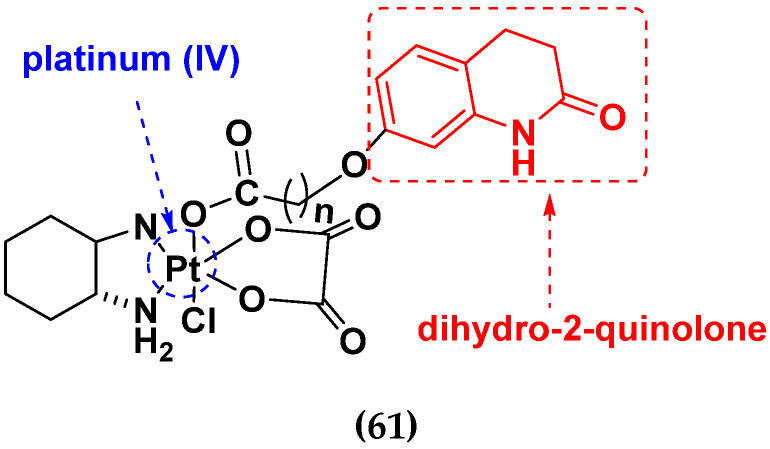
Structure of Platinum (iv) dihydro-2-quinolone hybrid derivatives (**61**).

Liu Z et al. (2021) prepared dihydro-2-quinolone (DHQLO) platinum (IV) compounds. Cytotoxic profiles of DHQLO platinum (IV) complexes were tested against five carcinoma cell lines including, ovarian cancer (SKOV-3), human cervical cancer cell (HeLa), human lung cancer (A549), murine colon cancer (CT-26), a cisplatin resistant cell line (A549R), and one normal human embryonic kidney cell line (293T). The antitumor activities of DHQLO platinum (IV) compounds were tested using the MTT assay with cisplatin and oxaliplatin as reference drugs. Cells were treated with drugs at different concentrations for 48 h, and the results were given as IC_50_ values. The structure of Platinum (iv) dihydro-2-quinolone hybrids is given in Figure 64 and the in vitro cytotoxic activities of hybrid compounds **61**(**a**–**e**) are listed in Table 51 [131].

**Figure 65:**
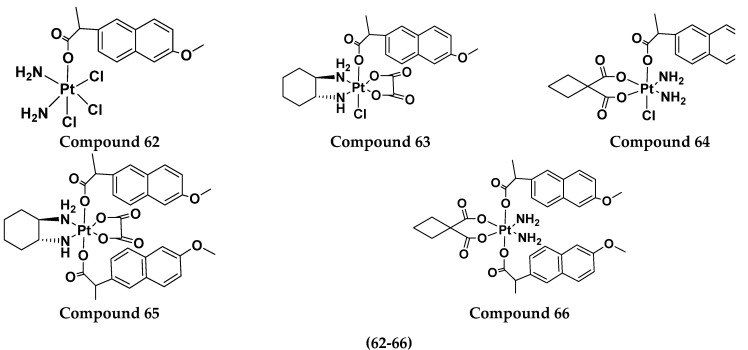
Structure of naproxen platinum (IV) hybrid derivatives.

Yan Chen et al. (2020) synthesized naproxen platinum(IV) hybrids (**62**−**66**, Figure 65) that inhibited, matrix metalloproteinases and caused DNA damage. The antitumor activities of naproxen platinum(IV) compounds (**62**−**66**) were tested against four tumor cell lines including human lung cancer (A549), human ovarian cancer (SKOV-3), murine colon cancer (CT-26) and a cisplatin resistant cell line (A549R).A human normal liver cell LO-2 was also evaluated. The results are given in Table 52. It was observed that compounds **62**–**66** displayed moderate to effective antitumor activities against the tested tumor cell lines [132].

**Figure 66:**
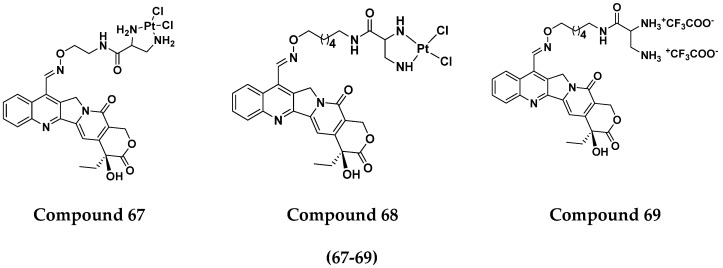
Structure of Camptothecin-linked platinum hybrid derivatives (**67**–**69**).

Raffaella Cincinelli et al. (2013) designed and synthesized camptothecin-linked platinum anticancer agents. Biological activity was tested on different cell lines, including non-small cell lung cancer (H460), osteosarcoma (U2OS), cell carcinoma cells (A431), and ovarian carcinoma (IGROV-1, A2780). These compounds showed growth inhibitory activity against a panel of human tumor cell lines, including sublines resistant to topotecan and platinum compounds. A general reduced potency with respect to TPT was observed in ovarian carcinoma IGROV-1 and A2780 cell lines. The structure of camptothecin-linked platinum anticancer hybrids is given in Figure 66 and the in vitro cytotoxic activities of hybrid compounds (**67**–**69**) are listed in Table 53 [133].

#### Platinum Based Drugs That Are FDA Approved or under Clinical Trial

Different platinum-based potent anticancer drugs their chemical structure, specific cancer types and current status are shown in Table 54.

### 3.11. Hydroxamic Acid Hybrids

**Figure 67:**
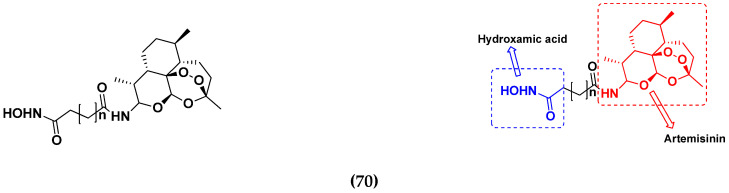
Structure of hydroxamic acid with artemisinin hybrid derivatives (**70**).

Xing Yan et al. (2016) synthesized hybrids of hydroxamic acid with artemisinin and evaluated their anticancer activity against different cell lines. Most of the synthesized compounds showed potent anticancer activity. Among them, compound **70**(**a**) exhibited excellent activity against various cell lines including HepG2, MCF-7 and HL-60 (human leukemia cell) with IC_50_ values of 2.50, 2.62, and 1.28 µM, respectively, and control suberoylanilide hydroxamic acid (SAHA) IC_50_ values of 0.31, 1.90, and 0.18 µM, respectively. Furthermore, they synthesized 14 compounds. Amongst them, only two compounds **70**(**a**), and **70**(**b**), showed excellent HDAC (histone deacetylases) selectivity with IC_50_ values of 29.31 and 22.7 µM, respectively. The structure of a hydroxamic acid artemisinin hybrid is shown in Figure 67 and the in vitro cytotoxic activities of hybrid compounds **70**(**a**–**c**) are listed in Table 55 [136].

**Figure 68:**
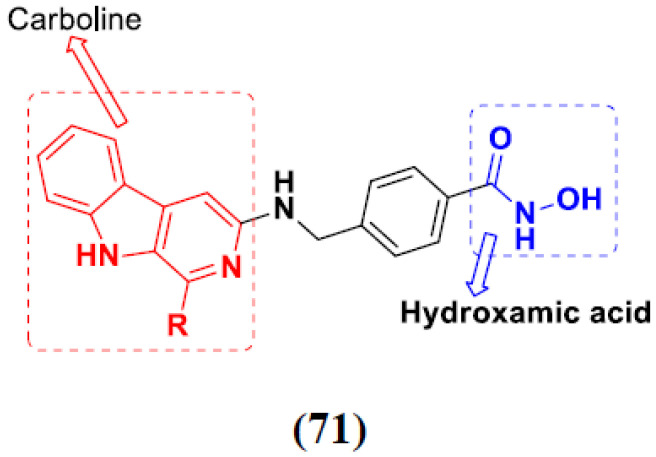
Structure of hydroxamate-β-carboline based hybrid derivatives (**71**).

Yong Ling et al. (2018) synthesized hydroxamate-β-carboline based novel hybrids and evaluated their antiproliferative activity against different cell lines. Most of the synthesized compounds displayed potent anticancer activity. Among them, compound **71**(**a**) exhibited potent activity against panel of cell lines including HCT116 (human colon cancer cell), SUMM-7721(human hepatocellular carcinoma cells), HepG2, MCF-7and Huh-7(human hepatocellular carcinoma cells) with IC_50_ values of 0.82, 1.06, 0.65, 2.25 and 1.52 µM, respectively, and control SAHA IC_50_ values of 5.53, 5.61, 6.27, 4.48 and 4.95 µM, respectively. The structure of novel hydroxamate-β-carboline based derivatives is given in Figure 68 and the in vitro cytotoxic activities of hybrid compounds **71**(**a**–**d**) are listed in Table 56 [137].

**Figure 69:**
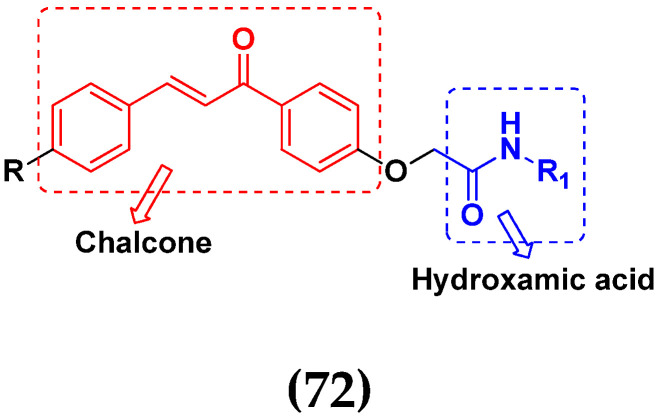
Structure of hydroxamic acid based chalcone derivatives (**72**).

M.F.A. Mohamed et al. (2017) synthesized hybrids of hydroxamic acid and chalcone derivatives and evaluate their antiproliferative activity against different cell lines. Most of the synthesized compounds displayed potent anticancer activity. Among them, **72**(**a**) exhibited potent activity against cancer cell lines including HEPG2, MCF-7 and HcF-116 (human colon cancer cell), having IC_50_ values of 0.62, 2.05 and 2.92 µM, respectively and control, SAHA having IC_50_ values of 3.33, 2.18 and 1.23, respectively. The structure of hydroxamic acid based chalcone derivatives is given in Figure 69 and the in vitro cytotoxic activities of hybrid compounds **72**(**a**–**d**) are listed in Table 57 [138].

**Figure 70:**
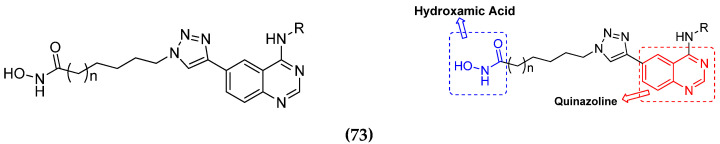
Structure of hydroxamic acid based 4-aminoquinazolin derivatives (**73**).

Chao ding et al. (2017) synthesized and investigated novel 6-(1,2,3-triazol-4-yl) 4-aminoquinazolin derivatives possessing a hydroxamic acid moiety for antiproliferative activity against two cell lines. Most of the synthesized compounds displayed potent anticancer activity. Among them, **73**(**a**) exhibited potent activity against cell lines including A549 and BT-474 (human breast cancer cells) with IC_50_ values of 0.51 and 3.63 µM, respectively. Controls were lapatinib (IC_50_: 1.740.28 and 0.100.02) and SAHA (IC_50_: 2.57 and 2.67). The structure of hydroxamic acid based 4-aminoquinazolin derivatives is given in Figure 70 and the in vitro cytotoxic activities of hybrid compounds **73**(**a**–**d**) (IC_50_, µM) are listed in Table 58 [139].

**Figure 71:**
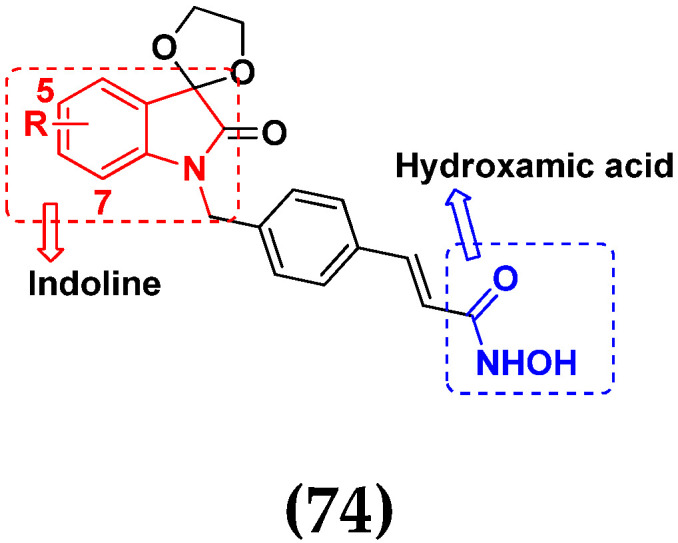
Structure of hydroxamic acid based indoline derivatives (**74**).

D.T.M. Dung et al. (2017) synthesized hybrids of indoline-based N-hydroxy propenamides and evaluated their antiproliferative activity against different cell lines. Most of the synthesized compounds displayed potent anticancer activity. Among them, compound **74**(**a**) exhibited potent actively against three different cell lines including SW620 (colon cancer), Aspe-1(prostate cancer) and PC-3 at with IC_50_ values 3.05, 6.83 and 7.30 (µM), respectively with control SAHA having IC_50_ values of 1.44, 7.04, 5.30 (µM), respectively. The structure of hydroxamic acid based indoline derivatives is given in Figure 71 and the in vitro cytotoxic activities of hybrid compounds **74**(**a**–**d**) are listed in Table 59 [140].

#### Hydroxamic Acid Based Hybrids That Are FDA Approved or under Clinical Trial

Hydroxamic acid based hybrids that are FDA approved or under clinical trial, and, their chemical structure and current status are listed in Table 60.

### 3.12. Ferrocene Hybrids

**Figure 72:**
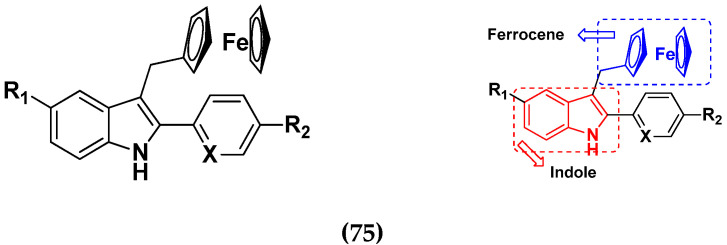
Structure of ferrocene-indole derivatives (**75**).

Quirante et al. (2011) synthesized ferrocene-indole based hybrids (Figure 72) and evaluated their cytotoxic activity against A549 cells using 5-fluorouracil (5-FU) as a positive control. They synthesized 14 molecules, and 12 showed cytotoxic activity at IC_50_ values below 100 µM, where 5-FU had an IC_50_ value of <5 µM. Among these molecules, ferrocene-indole hybrid **75**(**a**) showed the strongest cytotoxic activity with an IC_50_ value of 5 µM. Cytotoxic activities of hybrid compounds **75**(**a**–**d**) are listed in Table 61 [145].

**Figure 73:**
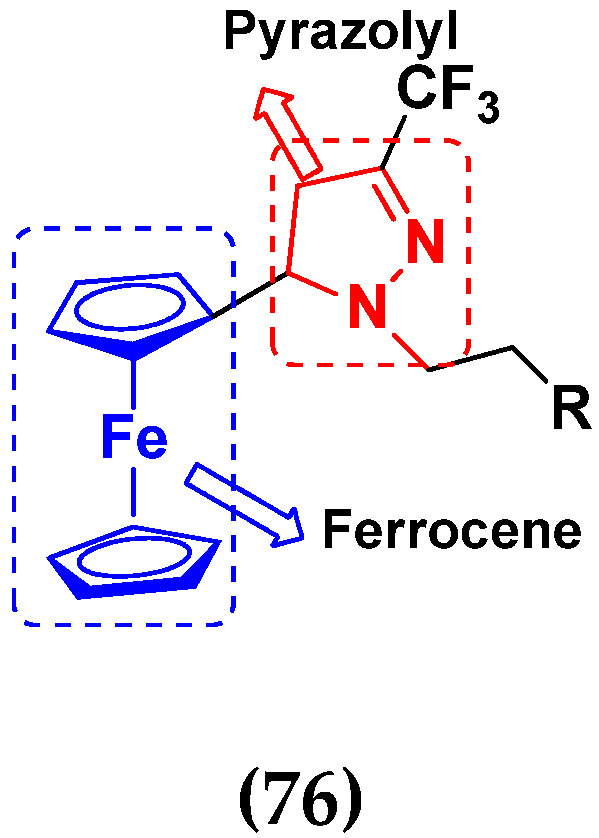
Structure of ferrocene containing a pyrazolyl derivative (**76**).

Xian-Feng Huang et al. (2014) synthesized novel hybrids of ferrocene containing pyrazolyl moieties and evaluated their anti-proliferative activity against different cell lines. Most of the synthesized compounds displayed potent anticancer activity. Among them, compound **76**(**c**) exhibited potent activity against three different cell lines; A549 (human lung cancer cell), Hep G2 (human liver cancer cell) and MDA-MB-45 (human breast cancer cell) with IC_50_ values of 4.44, 20.82, and 4.89 (µM), respectively. Control 5-Fluro Uracil had IC_50_ values of 16.2, 17.6, 2.80 and cisplatin 0.87, 0.74 and 1.14, respectively. The structure of ferrocene derivatives containing pyrazolyl moiety is given in Figure 73 and in vitro cytotoxic activities of hybrid compounds **76**(**a**–**d**) are listed in Table 62 [146].

**Figure 74:**
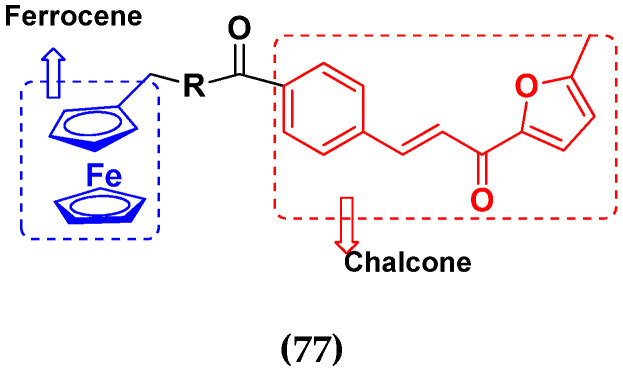
Structure of ferrocenyl-chalcone amide derivative (**77**).

Frans J. Smit et al. (2016) synthesized hybrids of ferrocenyl-chalcone amide, and evaluate their antitumor activity against different cell lines. Most of the synthesized compounds displayed potent anticancer activity. Among them, compound **77**(**a**) exhibited potent activity against three different cell lines, including Tk-10 (renal) UACC-62 (melanoma), and, MCF-7 at IC_50_ 2.4, 3.0 and 2.5 μM, respectively. Control parthenolide (PTD)-had IC_50_ values of 6.4, 15.0 and 5.8, respectively. The structure of ferrocenyl-chalcone amide derivatives are shown in Figure 74 and the in vitro cytotoxic activities of hybrid compounds **77**(**a**–**d**) are listed in Table 63 [147].

**Figure 75:**
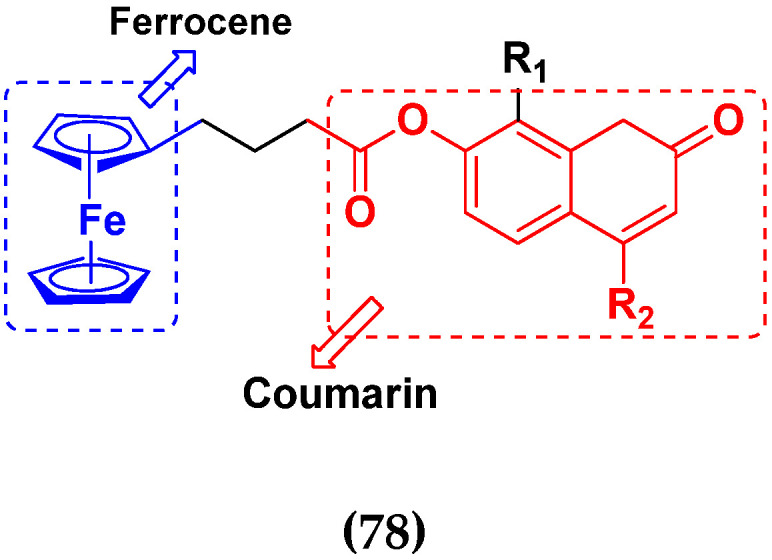
Structure of ferrocene-coumarin moiety derivative (**78**).

J.N. Wei et al. (2019) synthesized novel hybrids of ferrocene-coumarin moiety and evaluate their antiproliferative activity against six different human cancer cell lines. Most of the synthesized compounds displayed potent anticancer activity. Among them, compound **78**(**a**) exhibited potent activity against six different cell lines: BIU-87 (Human Bladder Cancer cell), SGC-790 (human cancer gastric cells), EC9706 and ECa1090 (human esophageal cancer cells), MCF-7 (human breast adenocarcinoma cells) and Jurkat (human leukemia cell) with IC_50_ values 1.09, 10.61, 25.89, 36.38, 12.10 and 53.01 (µM), respectively. Control adriamycin had IC_50_ values of 6.09, 5.44, 8.56, 6.52, 7.95, 4.50 µM, respectively. The structure of ferrocene-coumarin derivatives is given in Figure 75 and the in vitro cytotoxic activities of hybrid compounds **78**(**a**–**d**) are listed in Table 64 [148].

**Figure 76:**
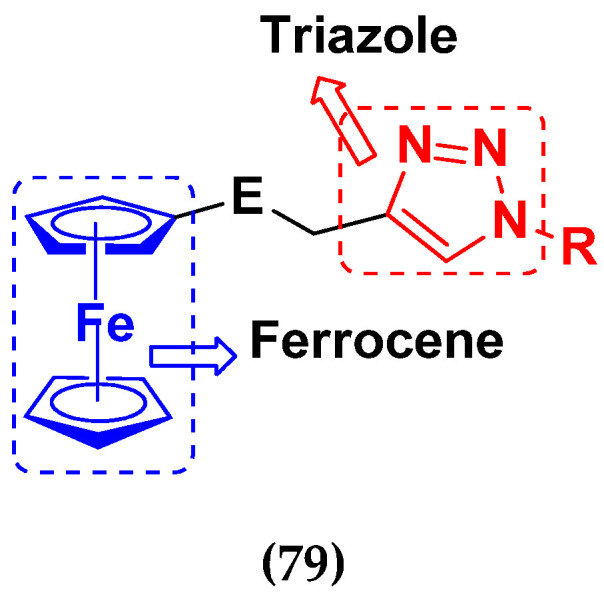
Structure of ferrocene-chalcogeno (sugar) triazole conjugate derivatives (**79**).

S. Panaka et al. (2016) synthesized hybrids of ferrocenyl chalcogeno (sugar) triazole conjugates and evaluated their antitumor activity against different cell lines. Most of the synthesized compounds displayed potent anticancer activity. Among them, compound **79**(**a**) exhibited potent activity against five different cell lines including A549 (human lung cancer cell), MDA-MB-231 (human breast cancer cell), MCF-7 (human breast adenocarcinoma cell), HeLa (immortal human cell) and HEK-293T (normal non-tumorigenic human embryonic kidney) at IC_50_ values 2.9, 3.35, 5.58 and 11.6 (μM), respectively. A control, doxorubicin, had IC_50_ values of 0.36, 0.47, 0.98 and 0.89, respectively. The structure of ferrocenyl-chalcogeno (sugar) triazole conjugates is given in Figure 76 and the in vitro cytotoxic activities of hybrid compounds **79**(**a**–**d**) (IC_50_, µM) are listed in Table 65 [149].

### 3.13. Curcumin-Based Hybrids

**Figure 77:**
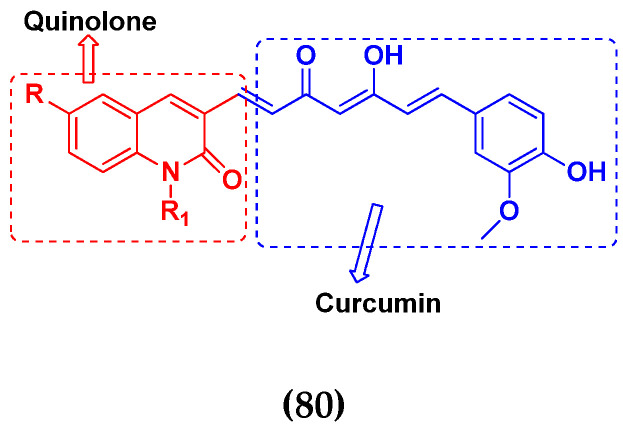
Structure of curcumin-quinolone derivatives (**80**).

Saiharish Raghavan et al. (2015) synthesized hybrids of curcumin and quinolone and evaluated their anticancer activity against different cell lines. Most of the synthesized compounds displayed potent anticancer activity. Among them, compound **80**(**a**) exhibited potent activity against four different cell lines including A549 (human lung cancer cell), MCF-7 (human breast adenocarcinoma cell), SKOV3 (ovarian cancer cell line) and H460 (human lung cancer cell) at IC_50_ values 23.9, 36.2,12.8, 21.75, respectively. The structure of curcumin-quinolone derivatives is given in Figure 77 and in vitro cytotoxic activities of hybrid compounds **80**(**a**–**d**) are listed in Table 66 [150].

**Figure 78:**
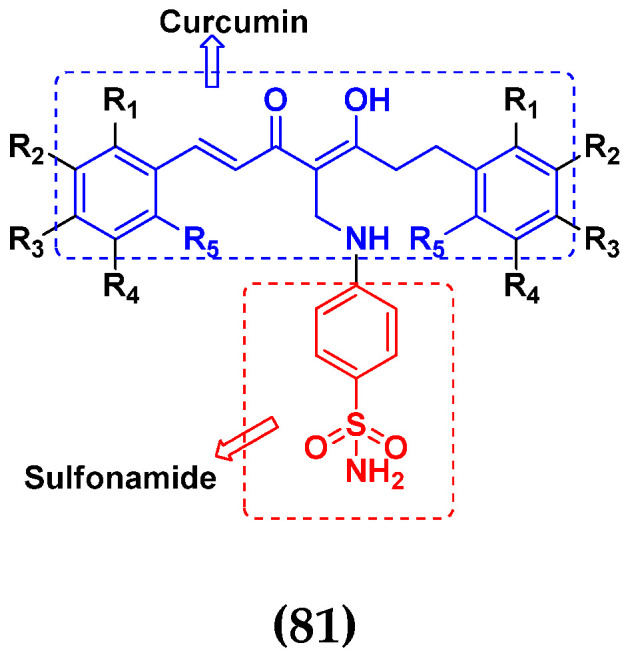
Structure of curcumin-sulfonamide derivatives (**81**).

G. Banuppriya et al. (2018) synthesized hybrids of curcumin and sulfonamides and evaluated their anticancer activity against two cell lines. Most of the synthesized compounds displayed potent anticancer activity. Among them, compound **81**(**a**) exhibited potent activity against two different cell lines including A549 (human lung cancer cell) and AGS (human gastric adenocarcinoma cell) at IC_50_ values 1.29 and 10.16 (µM), respectively with control Curcumin at IC_50_ values 25.33 and 20.76, respectively. The structure of curcumin-sulfonamide derivatives is given in Figure 78 and in vitro cytotoxic activities of hybrid compounds **81**(**a**–**d**) are listed in Table 67 [151].

**Figure 79:**
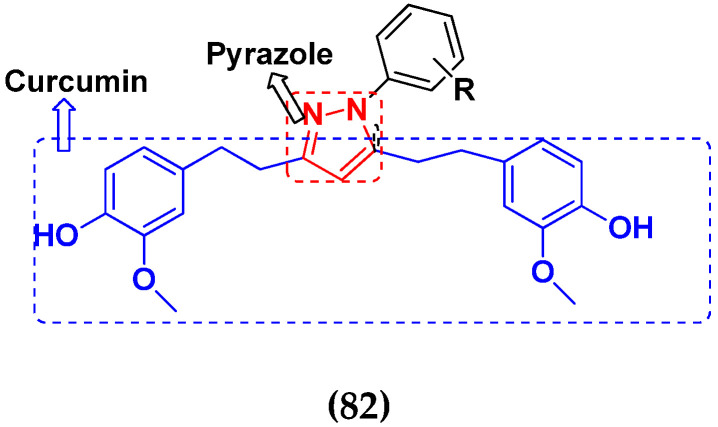
Structure of curcumin-pyrazole derivative (**82**).

H.R. Puneeth et al. (2016) synthesized hybrids of curcumin-pyrazole and evaluated their anticancer activity against different cell lines. Most of the synthesized compounds displayed potent anticancer activity. Among them, compound **82**(**a**) exhibited potent activity against four different cell lines including HeLa (human cervical cell), MCF-7 (human breast adenocarcinoma cell), K562 (human immortalized myelogenous leukemia cell) and HEK293T (normal non-tumorigenic human embryonic kidney) at IC_50_ values 45.54, 34.99, 25.55 and >1000 (µM), respectively with control Paclitaxel at IC_50_ values 11.61, 9.12, 8.43 and 1.43 (µM), respectively. The structure of curcumin-pyrazole derivatives is given in Figure 79 and in vitro cytotoxic activities of hybrid compounds **82**(**a**–**c**) (IC_50_, µM) are listed in Table 68 [152].

**Figure 80:**
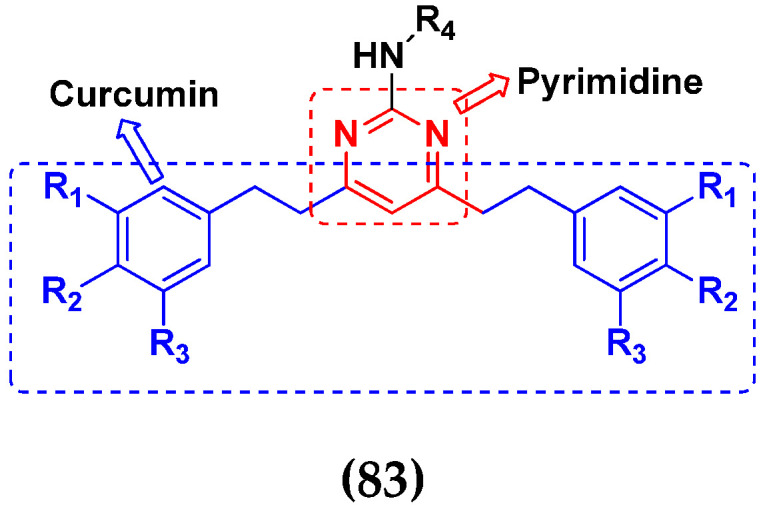
Structure of curcumin-pyrimidine derivatives (**83**).

Peiju Qui et al. (2013) synthesized hybrids of curcumin, pyrimidine and urea and evaluated their anticancer activity against different cell lines. Most of the synthesized compounds displayed potent anticancer activity. Among them, compound **83**(**a**) exhibited potent activity against two different cell lines including HT29 (human colon adenocarcinoma cell) and HCT116 (human colon cancer cell) at IC_50_ values 7.10.4 and 6.21.2 (µM), respectively with control 5-fluorouracil at IC_50_ 11.61, 9.12, 8.43 and 1.43 (µM), respectively. The structure of curcumin pyrimidine derivatives is given in Figure 80 and in vitro cytotoxic activities of hybrid compounds **83**(**a**–**c**) (IC_50_, µM) are listed in Table 69 [153].

**Figure 81:**
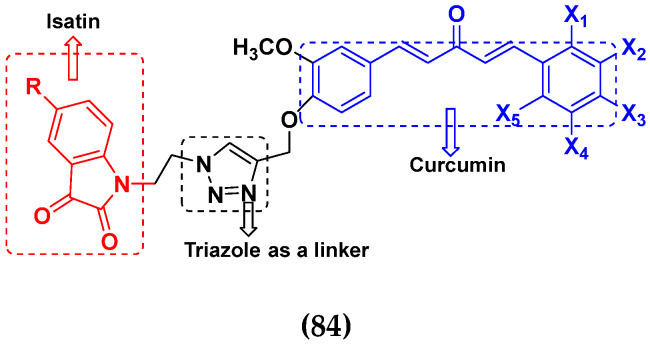
Structure of curcumin-isatin derivatives (**84**).

Sahil Sharma et al. (2015) synthesized hybrids of curcumin and isatin and evaluated their anticancer activity against different cell lines. Most of the synthesized compounds displayed potent anticancer activity. Among them, compound **84**(**a**) exhibited potent activity against four different cell lines including THP-1 (Leukemia), COLO-205 (Colon), HCT-(116) and PC-3 (Prostate) at IC_50_ values of 2.87, 4.15, 1.12 and 5.67 (µM), respectively. The structure of curcumin-isatin derivatives is given in Figure 81 and in vitro cytotoxic activities of hybrid compounds **84**(**a**–**c**) are listed in Table 70 [154].

### 3.14. Triazole-Based Hybrids

**Figure 82:**
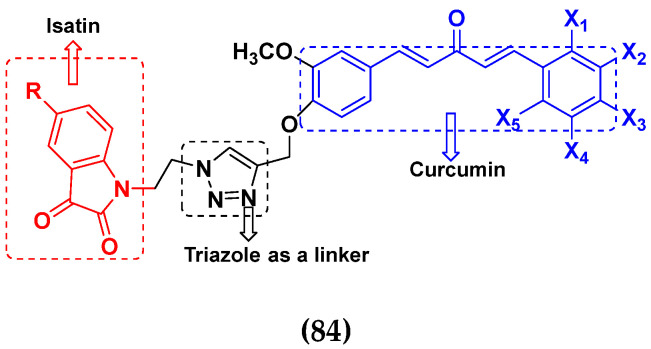
Structure of triazole-pyrimidine derivatives (**85**).

Li Ying et al. (2015) synthesized hybrids of triazole, pyrimidine and urea and evaluated their anticancer activity against different cell lines. Most of the synthesized compounds displayed potent anticancer activity. Among them, compound **85**(**a**) exhibited potent activity against four different cell lines including EC-109 (squamous cell carcinoma cells), MCF-7 (human breast adenocarcinoma cells), MGC-803 (immortal human cells) and B16-F10 (murine melanoma cells) at IC_50_ 2.96, 3.11, 3.60 and 4.55 (µM), respectively and with control 5-fluorouracil an IC_50_ of 11.61, 9.12, 8.43 and 1.43 (µM), respectively. The structure of triazole-pyrimidine derivatives is given in Figure 82 and in vitro cytotoxic activities of hybrid compounds **85**(**a**–**d**) are listed in Table 71 [155].

**Figure 83:**
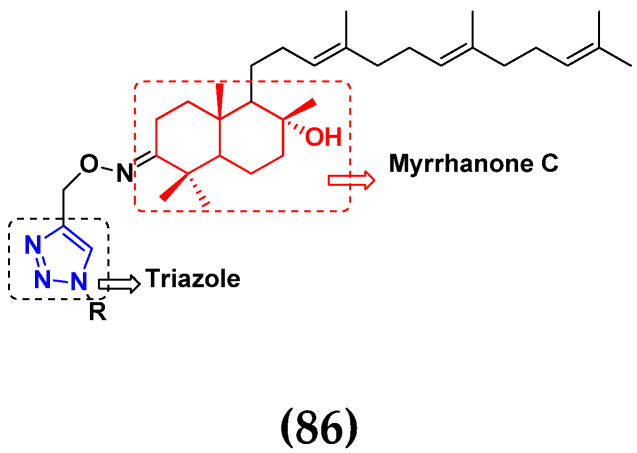
Structure of triazole–myrrhanore C derivatives (**86**).

Madasu Chandrashekhar et al. (2016) synthesized hybrids of triazole–myrrhanore Cand evaluated their anticancer activity against different cell lines. Most of synthesized compounds displayed potent anticancer activity. Among them, compound **86**(**a**) exhibited good anticancer activity against different cell lines including A549 (human lung cancer cell), Hela (human cervical cell), MCF-7 (human breast adenocarcinoma cell), DU-I45 (human prostate cancer cell) and HepG2 (human hepatocellular carcinoma cell) at IC_50_ 06.16, 07.76, 09.59, 08.83 and 09.52 (µM), respectively, with control doxorubicin 2.81, 2.57, 1.13, 1.41 and 3.01 (µM), respectively. The structure of triazole–myrrhanore C derivatives is given in Figure 83 and in vitro cytotoxic activities of hybrid compounds **86**(**a**–**c**) (IC_50_, µM) are listed in Table 72 [156].

**Figure 84:**
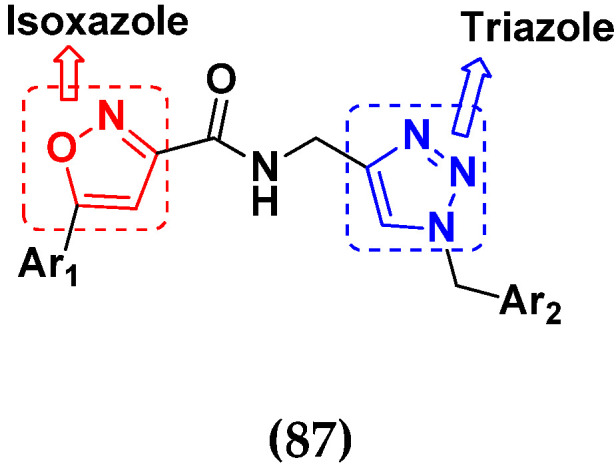
Structure of triazole-isoxazole derivatives (**87**).

Zahra Najafi et al. (2015) synthesized hybrids of triazole and isoxazole and evaluated their anticancer activity against different cell lines. Most of the synthesized compounds displayed potent anticancer activity. Among them, compound **87**(**a**) exhibited potent activity against two different cell lines including MCF-7 and T47D (breast cancer cell line) at IC_50_ > 100 and 27.7 µM, respectively with control etoposide at IC_50_ 7.5 and 7.9, respectively (µM). The structure of triazole-isoxazole derivatives is given in Figure 84 and in vitro cytotoxic activities of hybrid compounds **87**(**a**-**b**) (IC_50_, µM) are listed in Table 73 [157].

**Figure 85:**
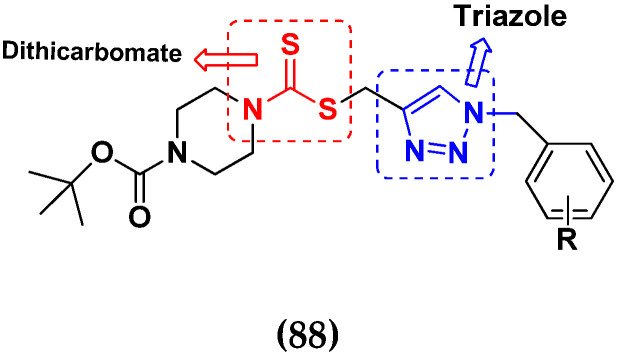
Structure of triazole-dithiocarbamate derivatives (**88**).

Y.C. Duan et al. (2013) synthesized hybrids of 1,2,3-triazole and dithiocarbamate and evaluated their anticancer activity against different cell lines. Most of the synthesized compounds displayed potent anticancer activity. Among them, compound **88**(**a**) exhibited potent activity against four different cell lines including MGC-803 (immortal human cell), MCF-7 (human breast adenocarcinoma cells), PC-3 (human pancreatic cancer cell) and EC-109 (squamous cell carcinoma cell) at IC_50_ 0.73, 5.67, 11.61 and 2.44 µM, respectively with control 5-fluorouracil at IC_50_ 7.01, 7.54, 27.07 and 3.34 µM, respectively. The structure of triazole-dithiocarbamate derivatives is shown in Figure 85 and in vitro cytotoxic activities of hybrid compounds **88**(**a**–**c**) (IC_50_, µM) are listed in Table 74 [158].

**Figure 86:**
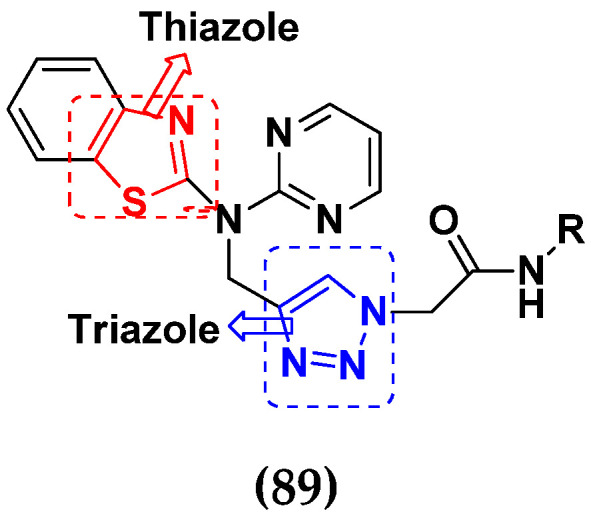
Structure of triazole-thiazole derivative (**89**).

R.M. Kumbhare et al. (2015) synthesized hybrids of triazole thiazole and evaluated their anticancer activity against different cell lines. Most of the synthesized compounds displayed potent anticancer activity. Among them, compound **89**(**a**) exhibited potent activity against four different cell lines including MCF-7 (human breast cancer), A549 (human lung cancer), A375 (human melanoma cancer), and MCF-10A (normal breast epithelial cells) with IC_50_ values of 2.12, 5.48, 4.7 and 29.33 µM, respectively. Control doxorubicin had comparative IC_50_ 0.12, 3.13, 7.2 and 24.0 µM and paclitaxel at IC_50_ 2.58, 4.9, 8.0, 38.0 µM, respectively. The structure of triazole based thiazole derivatives is given in Figure 86 and in vitro cytotoxic activities of hybrid compounds **89**(**a**–**c**) are listed in Table 75 [159].

### 3.15. Benzimidazole-Based Hybrids

**Figure 87:**
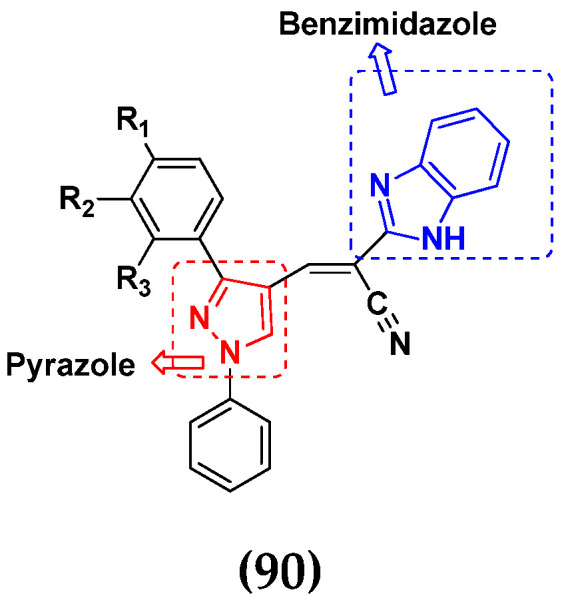
Structure of benzimidazole-pyrazole derivatives (**90**).

R. Sivaramakarthikeyan et al. (2020) synthesized hybrids of benzimidazole and pyrazole and evaluated their anticancer activity against different cell lines. Most of the synthesized compounds displayed potent anticancer activity. Among them, compound **90**(**a**) exhibited potent activity against three different cell lines including SW1990 (human pancreatic adenocarcinoma cell), AsPC1 (human pancreatic tumor cell), MRCS (marginal reticular cells) with IC_50_ values of 30.9, 32.8, 80.0 µM, respectively and control gemicitabine at IC_50_ values of 35.09, 39.27 and 54.17 µM, respectively. The structure of benzimidazole-pyrazole derivatives is given in Figure 87 and in vitro cytotoxic activities of hybrid compounds **90**(**a**–**c**) (IC_50_, µM) are listed in Table 76 [160].

**Figure 88:**
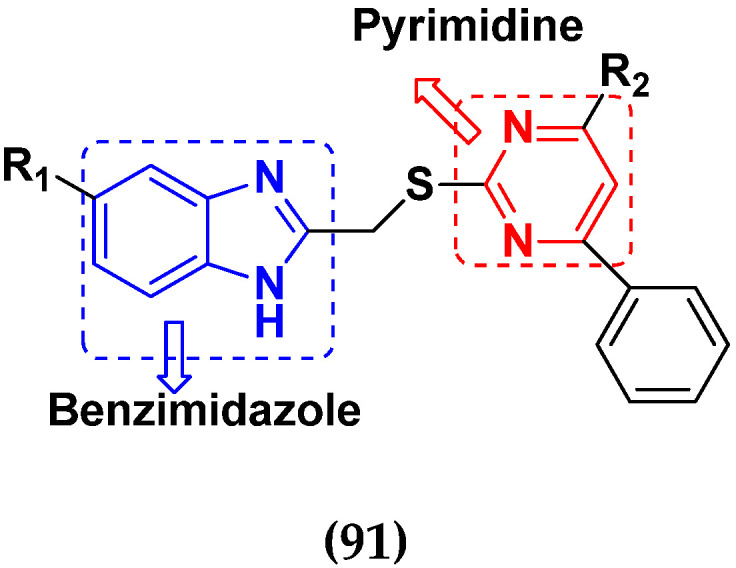
Structure of benzimidazole-pyrimidine derivatives (**91**).

Kun Pena Shao et al. (2014) synthesized hybrids of benzimidazole–pyrimidine and evaluated their anticancer activity against different cell lines. Most of the synthesized compounds displayed potent anticancer activity. Among them, compound **91a** exhibited potent activity against three different cell lines including MCF-7 (human breast adenocarcinoma cells), MGC-803 (immortal human cells), EC-9706 (esophagus squamous cell carcinoma) and SMMC-7721 (human hepatocellular carcinoma cells) with IC_50_ values of 1.43, 1.33, 3.33, 20.50 (µM), respectively with control 5-Fluorouracil had IC_50_ values 7.12, 3.45, 8.07 and 15.08 (µM), respectively. The structure of benzimidazole-pyrimidine derivatives is given in Figure 88 and in vitro cytotoxic activities of hybrid compounds **91**(**a**–**c**) (IC_50_, µM) are listed in Table 77 [161].

**Figure 89:**
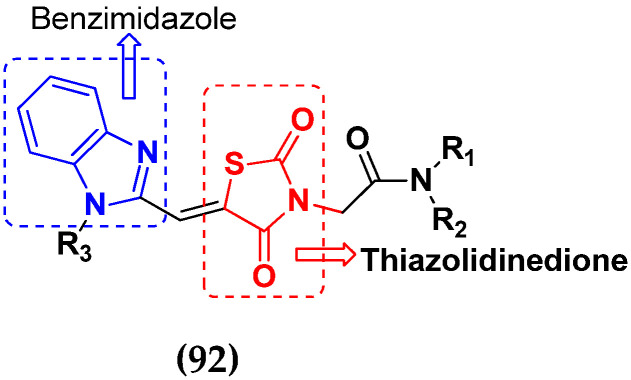
Structure of benzimidazole–thiazolidinedione derivatives (**92**).

Pankaj Sharma et al. (2016) synthesized hybrids of benzimidazole and thiazolidinedione and evaluated their anticancer activity against different cell lines. Most of the synthesized compounds displayed potent anticancer activity. Among them, compound **92a** exhibited potent activity against five different cell lines including PC-3, DU-145 (prostate cancer), MDA MB-231 (breast cancer), A549 (lung cancer) and MCF10A (normal breast epithelial cells) with IC_50_ values of 39.87, 31.41, 29.18, 11.46 and >100 (µM), respectively with control 5-Fluorouracil had IC_50_ values of 45.32, 40.58, 35.98, 30.47 (µM), respectively. The structure of benzimidazole-pyrimidine derivatives is given in Figure 89 and in vitro cytotoxic activities of hybrid compounds **92**(**a**–**c**) are listed in Table 78 [162].

**Figure 90:**
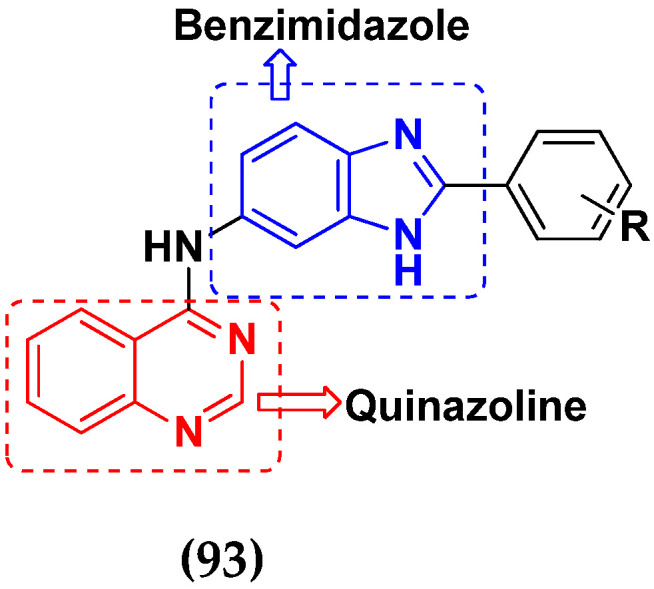
Structure of benzimidazole-quinazoline derivatives (**93**).

Lei Shi et al. (2014) synthesized benzimidazole-quinazoline hybrids and evaluated their anticancer activity against different cell lines. Most of the synthesized compounds displayed potent anticancer activity. Among them, compound **93a** exhibited potent activity against two different cell lines including Hep-G2 (human liver carcinoma cells) and MCF-7 (human breast adenocarcinoma cell) at IC_50_ 8.7 and 1.5 (µM), respectively. Control golvatinib had IC_50_ values of 65.5 and 49.6 µM, respectively. The structure of benzimidazole-quinazoline derivatives is given in Figure 90 and in vitro cytotoxic activities of hybrid compounds **93**(**a**–**c**) are listed in Table 79 [163].

**Figure 91:**
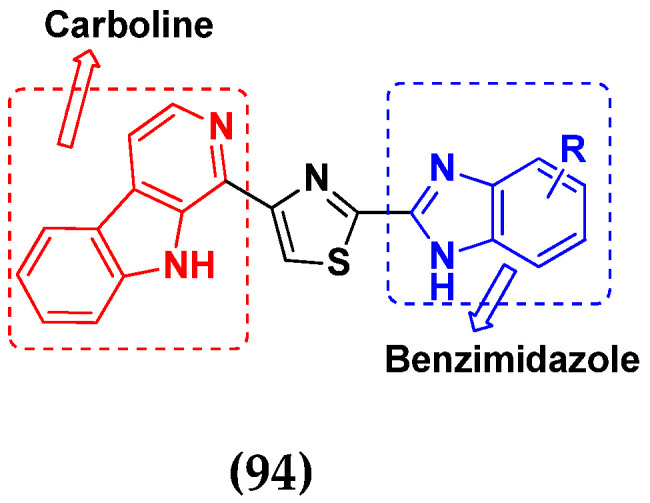
Structure of benzimidazole-β-Carboline derivatives (**94**).

Reddymasu Sireesha et al. (2021) synthesized hybrids of benzimidazole and β-carboline and evaluated their anticancer activity against different cell lines. Most of the synthesized compounds displayed potent anticancer activity. Among them, compound **94a** exhibited potent activity against four different cell lines, including MCF-7 (human breast cancer cell line), A549 (a human lung cancer cell line), Colo-205 (a human colon cancer cell line) and A2780 (a human ovarian cancer cell line) with IC_50_ values of 0.22, 1.55, 1.68 and 1.16 (µM), respectively, with the control etoposide having IC_50_ values of 2.11, 3.08, 0.13 and 1.31 (µM), respectively. The structure of benzimidazole–β-carboline derivatives is given in Figure 91 and in vitro cytotoxic activities of hybrid compounds **94**(**a**–**c**) are listed in Table 80 [164].

#### Benzimidazole Based Hybrids That Are FDA Approved or under Clinical Trials

Benzimidazole based hybrids that are FDA approved/under clinical trials and their current status are given in Table 81.

### 3.16. Isatin Containing Hybrids

In humans and other mammals, isatin is an endogenous compound that has a variety of pharmacological properties, including anticancer activity [169]. Human health is facing several difficulties in the modern medical period, especially with regard to human cancers. As a result, new therapies that selectively target tumor cells will unavoidably be added to the therapeutic arsenal for these cancers [170].

**Figure 92:**
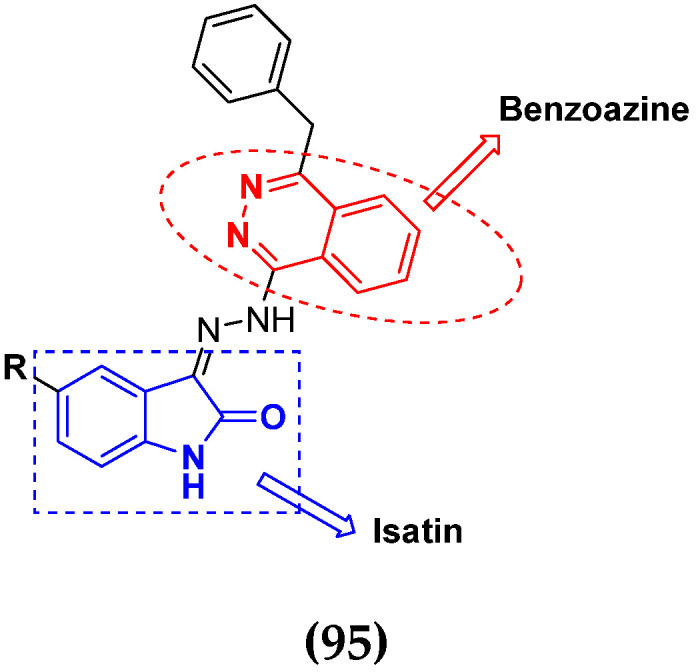
Structure of isatin-based benzoazine derivatives (**95**).

AZIZ et al. (2021) synthesized different sets of isatin-based benzoazine hybrids, i.e., isatin quinoxaline quinazoline and phthalazines hybrids. All the synthesized hybrids were evaluated in vitro for their antiproliferative activity against three human cancer cell lines, namely breast cancer (ZR-75), human colon cancer (HT-29) and lung cancer (A-549). The structure of isatin-based benzoazine derivatives is given in Figure 92 and in vitro cytotoxic activities of hybrid compounds **95**(**a**–**c**) are listed in Table 82 [171].

**Figure 93:**
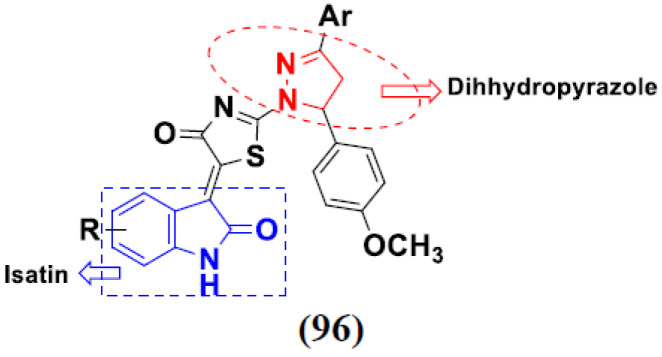
Structure of isatin-dihydropyrazole derivatives (**96**).

Meleddu et al. (2017) synthesized isatin-dihydropyrazole derivatives and then evaluated their capability to inhibit tumor cell growth against nine human cancer cell lines, namely A549 (lung carcinoma), IGR39 (melanoma), U87 (glioblastoma), MDA-MB-231 (breast cancer), MCF-7 (breast adenocarcinoma), and BT474 (invasive ductal carcinoma), H1299 (non-small cell lung carcinoma), BxPC-3 (pancreatic adenocarcinoma), SKOV-3 (ovarian cancer) and human foreskin fibroblasts. Sunitinib was the standard drug taken for evaluation of anticancer activity**.** The structure of dihydropyrazole isatin based hybrids is given in Figure 93 and the in vitro cytotoxicity of hybrid compounds **96**(**a**–**d**) against human cancer cell lines is shown in Table 83 [172].

**Figure 94:**
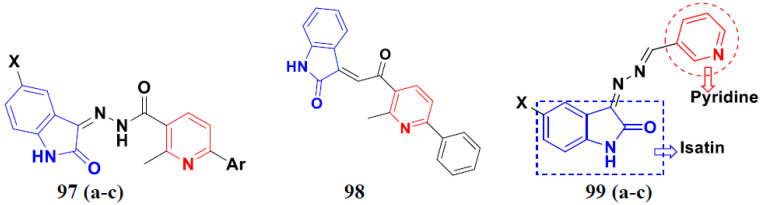
Structure of isatin-pyridine derivatives [**97**(**a**–**c**), **98**, and **99** (**a**–**c**)].

Wagdy et al. (2015) synthesized isatin-pyridine derivatives and tested their anti-proliferative activity against three human tumor cancer cell lines, i.e., hepatocellular carcinoma (HEPG2), lung cancer (A549), and breast cancer (MCF-7) using a sulforhodamine B (SRB) colorimetric assay. Doxorubicin has been used as a reference cytotoxic compound. The structure of pyridine isatin based hybrids is given in Figure 94 and in vitro cytotoxicity of compounds **97**(**a**–**c**), or **98**, **99**(**a**–**c**) against human cancer cell lines is shown in Table 84 [173].

**Figure 95:**
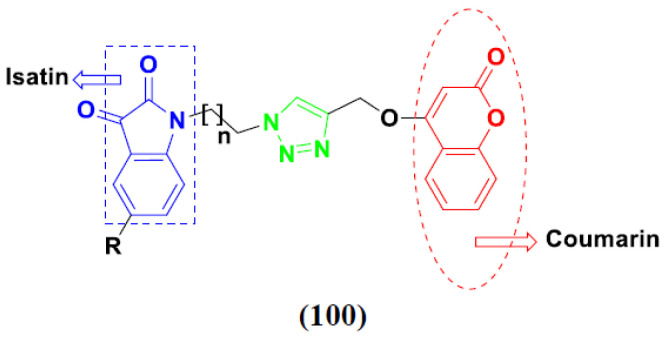
Structure of isatin-based coumarin derivatives (**100**).

Singh et al. (2015) synthesized isatin-coumarin hybrids which contain triazole as a linker, and investigated their in vitro cytotoxicity activity against four human cancer cell lines (COLO-205, THP-1, HCT-116 and PC-3) using sulforhodamine B19. The cells were given 48 h to multiply in the presence of a test substance. COLO-205, THP-1 and HCT-116 (three of the four cancer cell lines tested) were sensitive to the synthesized hybrids, and the THP-1 cancer cell line was the most susceptible to these hybrids, whereas PC-3 was found to be resistant. The structure of isatin-based coumarin hybrids is given in Figure 95 and in vitro cytotoxicity of compounds **100**(**a**–**d**) against human cancer cell lines is shown in Table 85 [174].

**Figure 96:**
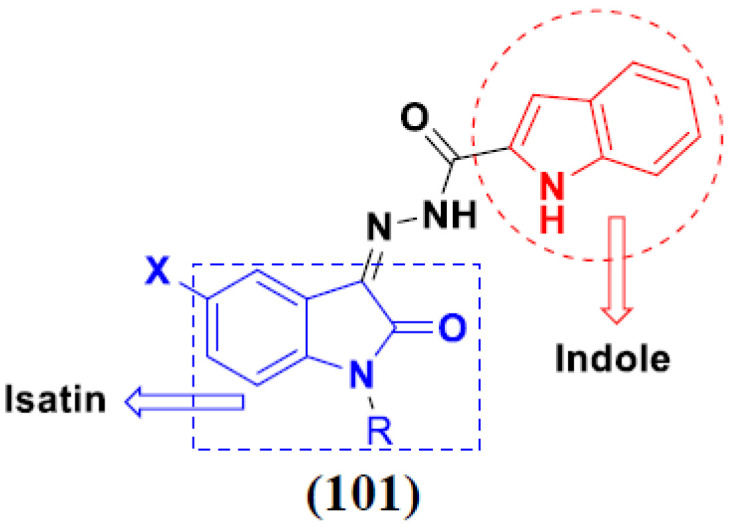
Structure of isatin-indole derivatives (**101**).

Wabli et al. (2017) synthesized isatin-indole derivatives and evaluated their antiproliferative activity on three cell lines: ZR-75 (human breast), HT-29 (colon) and A-549 (lung). Compound **101**(**d**) had an average IC_50_ value of 1.17 μM against the tested human cancer cell lines, making it the most active compound, with a potency approximately seven times greater than that of sunitinib. The structure of isatin-based indole hybrids is given in Figure 96 and in vitro cytotoxicity IC_50_ (µM) of compounds **101**(**a**–**d**) against human cancer cell lines is shown in Table 86 [175].

**Figure 97:**
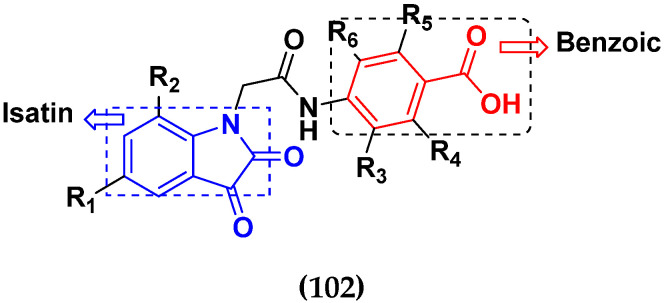
Structure of isatin-benzoic acid derivatives (**102**).

Panga et al. (2020) synthesized isatin-benzoic acid conjugates and tested their in vitro cytotoxic activity against MCF-7 and HeLa cell lines. All the synthesized isatin-benzoic acid conjugates showed moderate to strong cytotoxicity against both HeLa and MCF-7 cell lines with IC_50_ values ranging from 4.02 to 17.83 μM and 17.14 to 24.6 μM, respectively. Among all synthesized compounds, **102**(**b**) showed maximum activity on both cell lines. The structure of isatin-based benzoic acid hybrids is given in Figure 97 and in vitro cytotoxicity of compounds **102**(**a**–**d**) against human cancer cell lines is shown in Table 87 [176].

**Figure 98:**
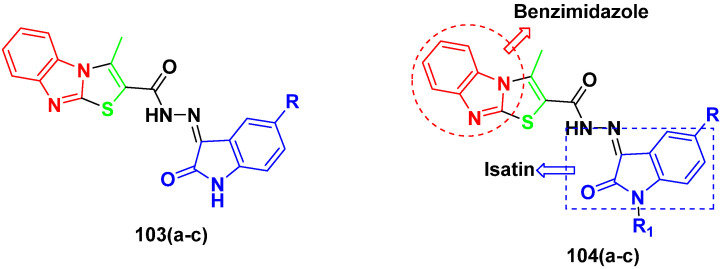
Structure of isatin-thiazolo benzimidazole derivatives (**103**,**104**).

Eldehna et al. (2016) synthesized isatin-thiazolo benzimidazole hybrids and evaluated their anti-proliferative efficacy against MCF-7 and MDA-MB-231 breast cancer cell lines by using sulforhodamine B colorimetric (SRB) assay. Staurosporine was utilized as a positive control, and the results were shown as IC_50_ values. Compounds **103**(**a**) and **104**(**a**) had maximum anticancer activity towards the tested cell lines. The structure of isatin-based thiazolo-benzimidazole hybrids is shown in Figure 98, and the in vitro cytotoxicity IC_50_ (µM) of compounds **103**(**a**–**c**), **104**(**a**–**c**) against human cancer cell lines is shown in Table 88 [170].

#### Isatin Containing FDA-Approved Hybrids

**Figure 99:**
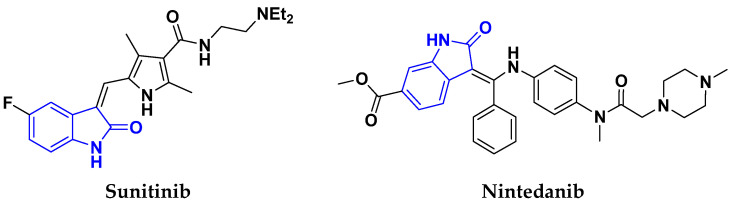
The isatin moiety as FDA-approved anticancer drug.

Isatin containing FDA-approved hybrids are depicted in Figure 99 [177].

### 3.17. Pyrrolo-Benzodiazepines Based Hybrids

Pyrrolo-benzodiazepines (PBD) are naturally found in many actinomycetes species. PBD block transcription factors and promote DNA replication by binding covalently to DNA, and thus inhibits cell growth [178].

**Figure 100:**
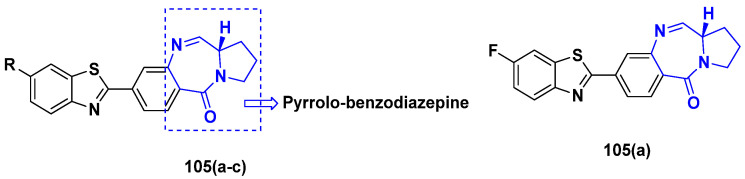
Structure of pyrrolo-benzodiazepine hybrids and the most promising compound **105a**.

Bose et al. (2012) synthesized hybrids of pyrrole and benzodiazepine and tested cytotoxic activities of the synthesized compounds was in vitro against five tumor cell lines: THP-1 (human acute monocytic leukemia), U-937 (human histiocytic lymphoma), HL-60 (human promyelocytic leukemia), Jurkat (human T-cell leukemia) and A-549 (lung carcinoma). Among the synthesized compounds, **105**(**a**) was the most potent. The structure of pyrrolo-benzodiazepine hybrids is given in Figure 100 and the in vitro cytotoxicity IC_50_ of compounds **105**(**a**–**c**) against human cancer cell lines is shown in Table 89 [179].

**Figure 101:**
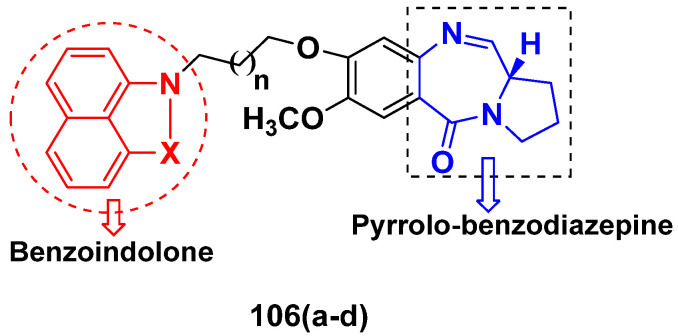
Structure of pyrrolo-benzodiazepine-based benzoindolone derivative (**106**).

Kamal et al. (2012) synthesized pyrrolo-benzodiazepine conjugated with benzo indolone derivatives. The synthesized derivatives were assessed for their anticancer activity in human cancer cell lines of the lung, skin, colon and prostrate by using the MTT assay. These new conjugates exhibited encouraging anticancer activity, with IC_50_ values ranging from 1.05 to 36.49 µM. Doxorubicin and DC81, the positive controls, displayed IC_50_ values in the range of 0.03–2.51 µM and 0.86–1.65 µM, respectively. Compound **106**(**d**) had the maximum anticancer activity. The structure of pyrrolo-benzodiazepine-based benzoindolone hybrids is given in Figure 101 and in vitro cytotoxicity of compounds **106**(**a**–**d**) against human cancer cell lines is shown in Table 90 [180].

**Figure 102:**
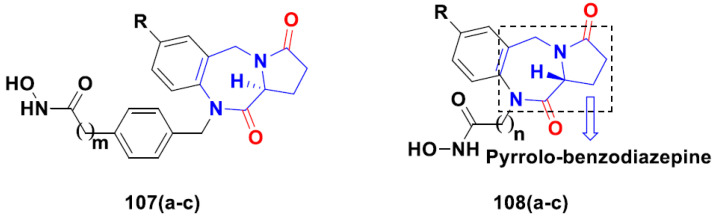
Structure of pyrrolo-benzodiazepine-dione derivatives [**107**(**a**–**c**),**108**(**a**–**c**)].

Li et al. (2021) synthesized new pyrrolo [2,1-*c*] [1,4] benzodiazepine-3,11-dione (PBD) derivatives and evaluated their HDAC6 inhibitory effect. When R = H, the activity of the linker with the benzene ring was moderate. However, when the linker was extended by one methylene, the compound’s activity drastically dropped (**107a** vs. 1**07b**). The structure of pyrrolo-benzodiazepine-dione hybrids is given in Figure 102 and the in vitro IC_50_ of compounds [**107**(**a**–**c**),**108**(**a**–**c**)] against HDAC6 enzyme is shown in Table 91 [181].

**Figure 103:**
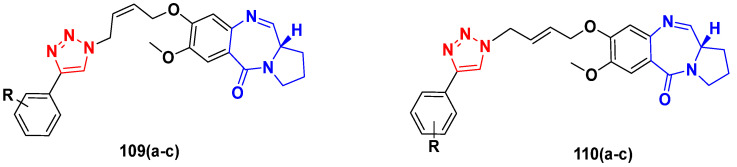
Structure of triazole-pyrrolo-benzodiazepines derivative [**109**(**a**–**c**),**110**(**a**–**c**)].

Chen et al. (2013) synthesized a new series of PBD-triazole hybrids and their cytotoxicity was investigated on various mouse and human cells, taking tubastatin A as a positive control. Cis-hybrids (**109a**–**c**) were much more cytotoxic than trans isomers (110**a**–**c**) in sensitive melanoma (A375). Compound **109a** exhibited a higher inhibitory activity compared to that of other agents on A375 cells. The structure of triazole pyrrolo-benzodiazepine hybrids is given in Figure 103 and the in vitro cytotoxicity of compounds [**109**(**a**–**c**), **110**(**a**–**c**)] against cancer cell lines is shown in Table 92 [182].

#### FDA Approved Drugs Containing Pyrrolo-Benzodiazepines

**Figure 104:**
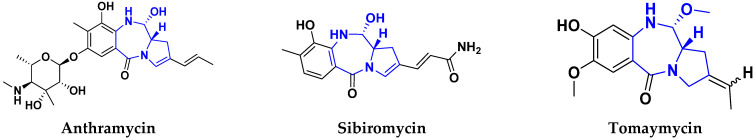
Pyrrolo-benzodiazapine containing FDA approved anticancer drug.

FDA approved drugs containing pyrrolo-benzodiazepines include tomaymycin, sibiromycin and neothramycin, which are depicted in Figure 104 [178].

### 3.18. Chalcone-Based Hybrids

**Figure 105:**
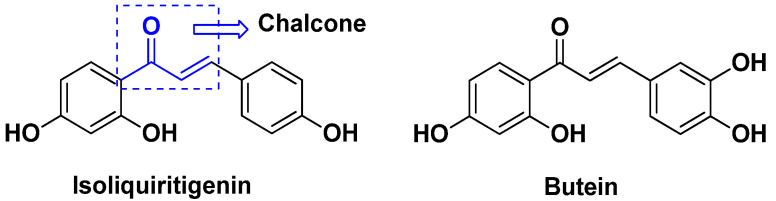
Naturally occurring phytoconstituents containing chalcone.

Chalcone compounds are one of the most important fundamental categories of natural products as they are abundant within tea leaves, fruits and vegetables, fruits, and are of great interest because of their pharmacological effectiveness in treating many diseases. Some of the naturally occurring chalcones are depicted in Figure 105 [183].

One of the most significant bioactive substances with a chalcone structure (isoliquiritigenin) was isolated from liquorice roots. Butein, a physiologically active flavonoid found in *Rhus verniciflua* (Stokes’ bark), has been shown to have strong anticancer effects on a variety of cancer types.

Chalcone-based hybrid compounds have the potential to improve selectivity and anticancer activity while also overcoming drug resistance. Therefore, combining the chalcone moiety with additional anticancer pharmacophores is a promising strategy for creating new anticancer drugs. In recent times, a number of chalcone hybrids have been prepared and evaluated for their cytotoxic activity; some of them are found to have remarkable activity both in vitro and in vivo [184].

**Figure 106:**
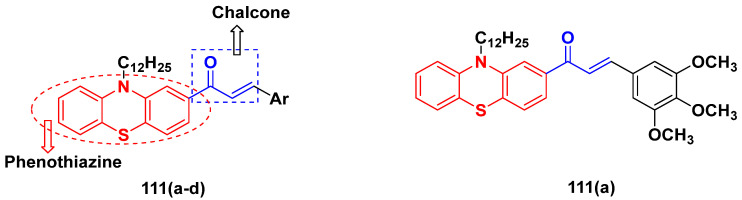
Structure of chalcone-based phenothiazine hybrids and the most promising compound **111a**.

Zahrani et al. (2020) synthesized chalcone-based phenothiazine derivatives and evaluated their cytotoxic activity against two carcinoma cell lines (human breast cancer cell line MCF-7 and human hepatocellular carcinoma HEPG-2 cells) compared with anticancer standard drugs cisplatin and doxorubicin under the similar conditions following the MTT (methylthiazol-tetrazolium) colorimetric assay. **111**(**a**) and **111**(**b**) were the most effective compounds with IC_50_ values of 7.14 µg/mL and 7.6 g/mL, respectively. The structure of chalcone based phenothiazine hybrids is shown in Figure 106 and the in vitro cytotoxicity of compounds **111**(**a**–**d**) against cancer cell lines is shown in Table 93 [183].

**Figure 107:**
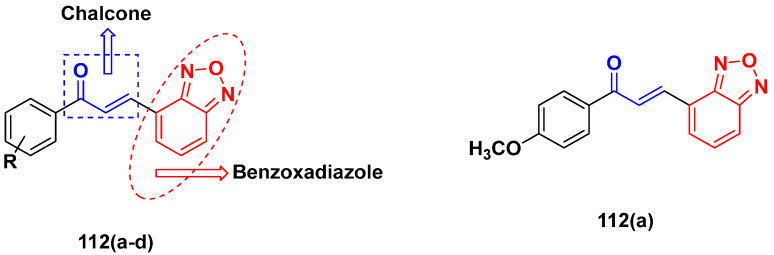
Structure of chalcone-based benzoxadiazole hybrids and the most promising compound **112a**.

Shivapriya et al. (2021) produced chalcone benzoxadiazole hybrids and evaluated their cytotoxic activity against the human epidermal carcinoma (KB) cell line using MTT assay. Chalcone-benzoxadiazole hybrids were prepared through the Claisen–Schmidt condensation reaction. The structure of chalcone-based benzoxadiazole hybrids is given in Figure 107 and the in vitro cytotoxicity of compounds **112**(**a**–**d**) against cancer cell lines is shown in Table 94 [185].

**Figure 108:**
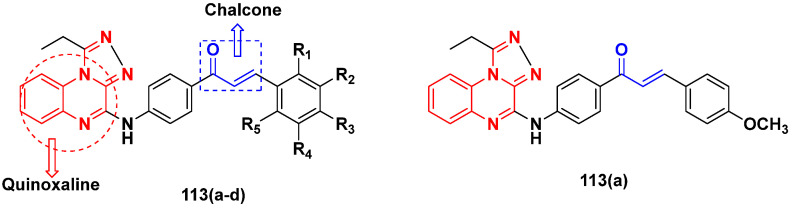
Structure of chalcone-based triazolo-quinoxaline hybrids and the most promising compound **113a**.

Alswah (2017) et al., designed and synthesized a series of triazolo-quinoxaline-chalcone derivatives **113a**–**d,** and evaluated their cytotoxic activity against three target cell lines: human breast adenocarcinoma (MCF-7), human colon carcinoma (HCT-116) and human hepatocellular carcinoma (HEPG-2) using the MTT assay method with doxorubicin as a reference drug. The initial results showed that some of the chalcones exhibited significant antiproliferative effects against most of the cell lines, with selective or non-selective behavior, with IC_50_ values found to be in the 1.65 to 34.28 µM range. Compound **113**(**a**) showed maximum cytotoxic activity towards given cell lines. The structure of chalcone-based triazolo-quinoxaline hybrids is given in Figure 108 and the in vitro cytotoxicity of synthesized hybrids against cancer cell lines are shown in Table 95 [186].

**Figure 109:**
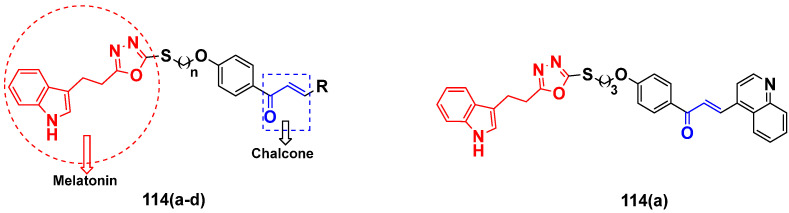
Structure of chalcone based melatonin hybrids and the most promising compound **114a**.

Yepes et al. (2021) synthesized a new series of hybrid molecules by inclusion of two scaffolds: chalcone and melatonin. To achieve this goal, biologically active chalcone was attached via a non-hydrolizable thioalkyloxy linker to the corresponding melatonin bioisostere scaffold. The anticancer activity of the synthesized hybrids was evaluated against an in vitro model of colorectal cancer. In this study, two cell lines were used, i.e., the non-malignant (CHO-K1) and human colon adenocarcinoma cells (SW480). The reference drug used was 5-FU. Compound **114**(**a**) maximum activity. The structure of chalcone based melatonin hybrids is given in Figure 109 and in vitro cytotoxicity of compounds **114**(**a**–**d**) against cancer cell lines is shown in Table 96 [187].

**Figure 110:**
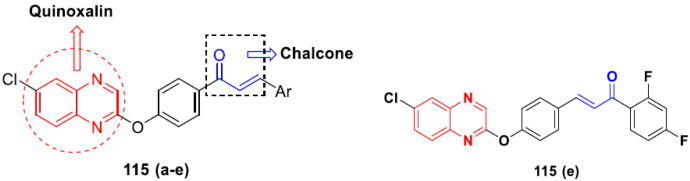
Structure of chalcone-based quinoxalin hybrids and the most promising compound **115e**.

Ma et al. (2021) synthesized chalcone-based quinoxalin as anti-cancer hybrids. All of the synthesized compounds (**115a**–**e**) were tested in vitro for their anticancer activities against, PC12, BPH-1 and MCF-7 cells using the MTT assay. With IC_50_ values in the micro molar range (9.1–98.7 mM) against all tested cancer cells, all synthesized compounds demonstrated moderate to good antiproliferative activity and compound **115**(**e**) showed maximum cytotoxic activity. The structure of chalcone-based quinoxalin hybrids is given in Figure 110 and the in vitro cytotoxicity of compounds **115**(**a**–**e**) against cancer cell lines is shown in Table 97 [188].

### 3.19. Coumarin-Based Hybrids

The coumarin scaffold (2H-1-benzopyran-2-one) is extensive in nature, and it’s derivatives exhibit various antibacterial, antifungal, antimalarial, and anticancer pharmacological properties.

**Figure 111:**
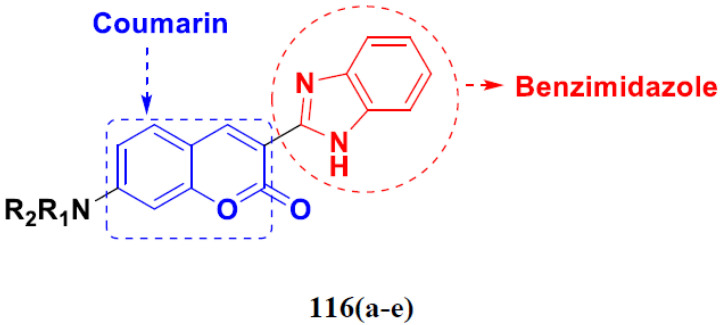
Structure of coumarin-benzimidazole derivatives **116**(**a**–**e**).

Kamaldeep Paul et al. (2013) synthesized coumarin-benzimidazole hybrids. The synthesized compounds showed potent activity against leukemia cancer cells (CCRF-CEM, HL-60(TB), K-562, RPMI-8226), colon cancer cells (HCT-116, HCT-15), melanoma cancer cells (LOX IMVI, UACC-257) and breast cancer cells (MCF7, T-47D). The compounds containing substitution (NR_1_R_2_) were the most potent with inhibition of most of the cell lines. The compound with ethanolamine as a substituent (NR_1_R_2_) at position 7 of the coumarin-benzimidazole scaffold, was the most potent synthesized compound. The structure of coumarin-benzimidazole hybrids is given in Figure 111 and the in vitro % growth inhibition of compounds **116**(**a**–**e**) against cancer cell lines is shown in Table 98 [189].

**Figure 112:**
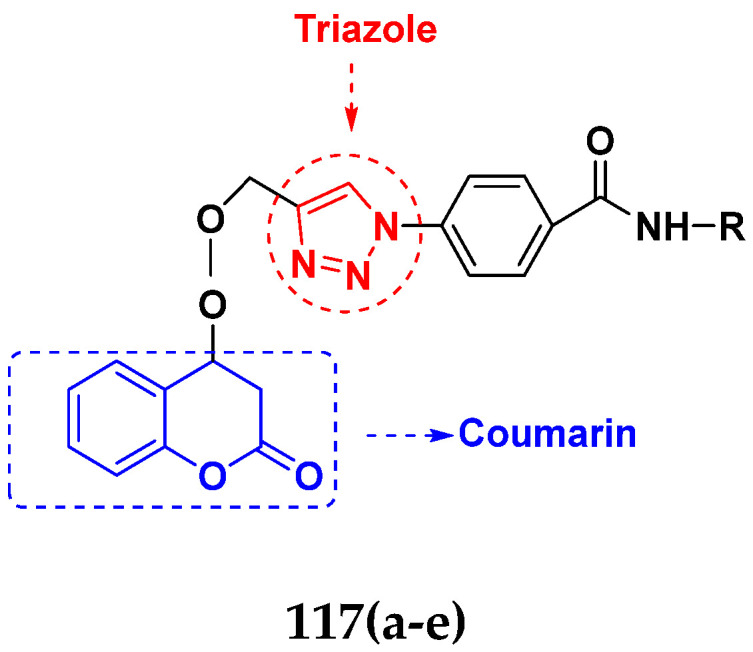
Structure of coumarin containing 1,2,3-triazole derivatives **117**(**a**–**e**).

R. An et al. synthesized amide containing 1,2,3-triazole hybrids, which showed moderate to excellent activity against MDB-MB 231 cell lines under both normoxic and hypoxic conditions. The structure of coumarin-triazole hybrids is given in Figure 112 and the in vitro IC_50_ of compounds **117** (**a**–**e**) against cancer cell lines are shown in Table 99 [190].

H.A. Elshemy et al., (2017) synthesized coumarin-chalcone hybrids having anticancer activity against HEPG2, leukemia, and WI-38 cell lines. All coumarin-chalcone hybrids had potent activity against HEPG2 and K562, and weak activity against WI-38 cell. Among the coumarin-chalcone hybrids, 4-methoxyphenyl chalcone **118**(**a**) was more potent than 3,4-dimethoxyphenyl chalcone **118**(**b**), which showed higher activity than chalcone with 3,4,5-trimethoxyphenyl moiety **118**(**c**).

**Figure 113:**
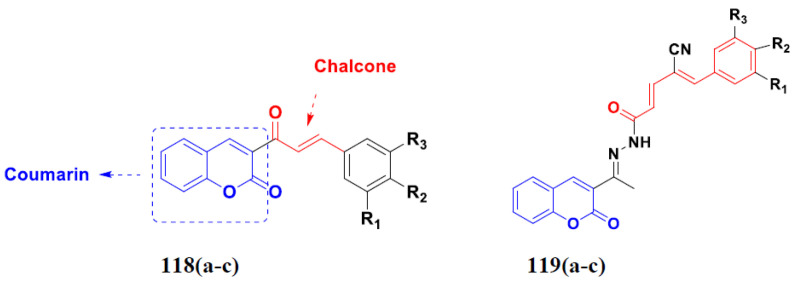
Structure of coumarin containing chalcone derivative [**118**(**a**–**c**),**119**(**a**–**c**)].

In the coumarin-acrylohydrazide series, compound **119**(**c**) showed the highest activity against a leukemia cell line (k562) while compound (**119a** and **119b**) showed potent activity against the HEPG2 cell line and moderate activity against the WI-38 cell line. The structure of coumarin containing chalcone hybrids is shown in Figure 113 and the in vitro IC_50_ (µM) of compounds [**118**(**a**–**c**),**119**(**a**–**c**)] against cancer cell lines is shown in Table 100 [191].

**Figure 114:**
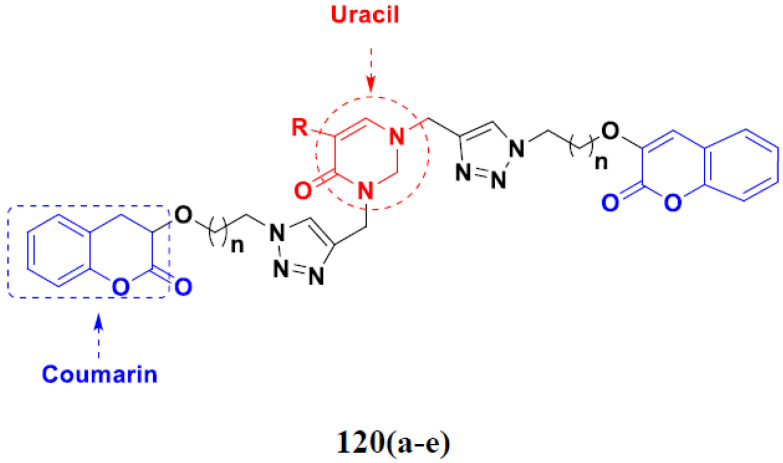
Structure of coumarin-based uracil derivatives **120**(**a**–**e**).

Mohit Sanduja et al. (2020) synthesized uracil-coumarin-based compounds as potent anticancer hybrid compounds. The uracil-coumarin hybrid compounds inhibited the MCF-7 cancer cell proliferation more effectively compared to standard 5-FU. The most potent compound 120b (GI_50_ = 1.55µM) contained A with fluorine atom as R with two carbon chain lengths between triazole and coumarin moieties. The structure of coumarin containing uracil hybrids is given in Figure 114, and in vitro GI_50_ (µM) of compounds **120**(**a**–**e**) against cancer cell lines is shown in Table 101 [192].

**Figure 115:**
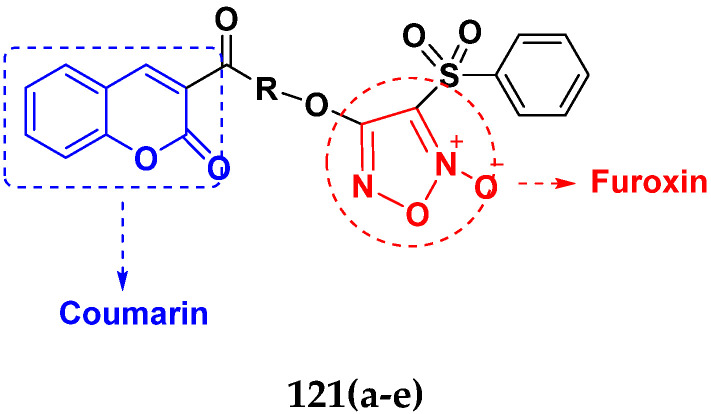
Structure of coumarin-based furoxin derivatives **121**(**a**–**e**).

Zhuo zing et al., (2018) synthesized novel coumarin-based furoxin hybrids which had potent activity against Hela cell proliferation. The compound **121**(**e**) had the highest antiproliferative activity and was more potent than standard, doxorubicin. The structure of coumarin containing furoxin hybrids is given in Figure 115 and the in vitro IC_50_ of compounds **121**(**a**–**e**) against cancer cell lines is shown in Table 102 [193].

### 3.20. Nitrogen Mustard Based Hybrids

**Figure 116:**
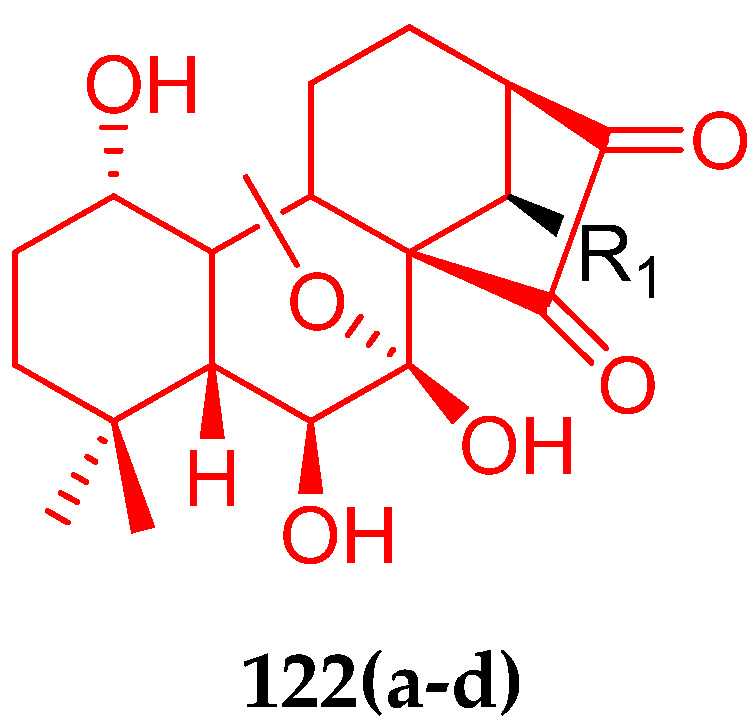
Structure of nitrogen mustard contain oridonin derivatives **122**(**a**–**d**).

Shengtao Xu et al. (2014) synthesized natural oridonin bearing nitrogen mustard hybrid as potential anticancer compound. The hybrid showed activity against the multidrug resistant cell lines SW620/AD300 and NCL-H460/MX20. Compound **122**(**b**) induced apoptosis and affected cell cycle progression in human hepatoma (Bel-7402) cells. The structure of nitrogen mustard contain oridonin hybrids is given in Figure 116 and the in vitro IC_50_ of compounds **122**(**a**–**d**) against the cancer cell line (Bel-7402) is shown in Table 103 [194].

**Figure 117:**
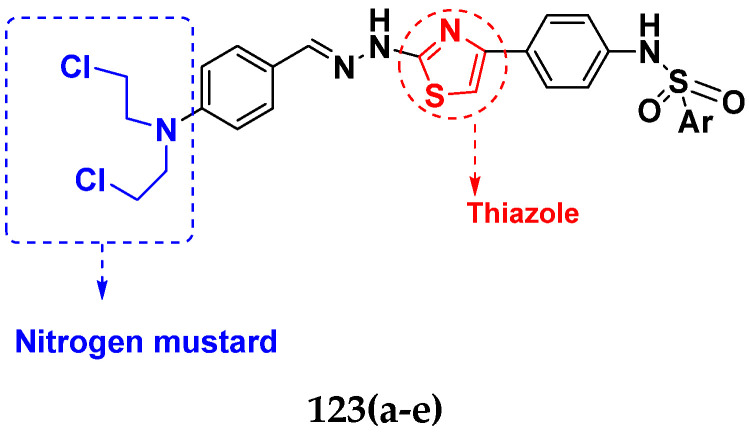
Structure of nitrogen mustard-containing thiazole derivatives **123**(**a**–**e**).

Laczkowski et al. (2016) synthesized nitrogen mustard-based thiazole hybrid drugs that showed antiproliferative activity against human cancer cell lines MV4-11, A549, MCF-7 and HCT-116. Among the all derivatives, **123**(**a**–**e**) was most potent and exhibited activity against human leukemia (MV4-11) cells. The compounds **123**(**b**) and **123**(**e**) had potent activity against MCF-7 and HCT116. The structure of nitrogen mustard contain thiazole hybrids is given in Figure 117 and the in vitro IC_50_ of compounds **123**(**a**–**e**) against cancer cell lines is shown in Table 104 [195].

**Figure 118:**
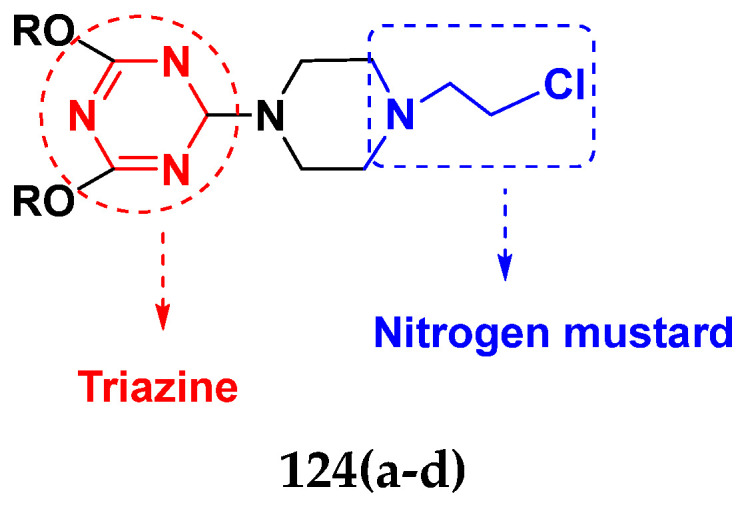
Structure of nitrogen mustard-containing triazine derivatives **124**(**a**–**d**).

Kolesinska et al. (2012) synthesized hybrids containing a nitrogen mustard-triazine scaffold as potent anticancer compounds. These were prepared by rearrangement of mono, bis and tris-(1,3,5-triazin-2-yl)-1,4-diazacyclo [2.2.2] octanium chlorides leading to the formation of 2-chloroethylamino fragments attached to 1,3,5-triazine via one, two or three piperazine, respectively. Their cytotoxicity and alkylating activity depended on substituents on the triazine ring and nitrogen mustard. Among the above mentioned compounds, 124(**b**) and 124(**d**) were the most potent. The structure of nitrogen mustard-containing triazine hybrids is shown in Figure 118 and the in vitro IC_50_ of compounds **124**(**a**–**d**) against cancer cell lines is shown in Table 105 [196].

**Figure 119:**
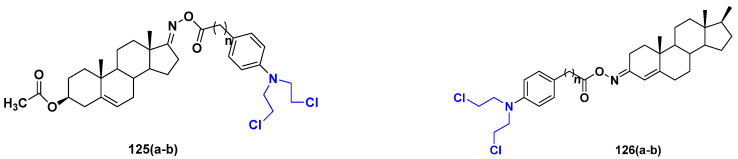
Structure of androstane oxime-nitrogen mustard hybrids [**125**(**a**-**b**),**126**(**a**-**b**)].

Acharya et al. (2017) synthesized androstene oxime-nitrogen mustard hybrids and nitrogen mustard conjugates of various steroidal oximes. The conjugation was achieved by oxime-ester linkage. The 17-E-steroidal oxime benzoic acid mustard ester, 3β acetoxy-17E-[p-(N,N-bis(2-chloroethyl)amino]benzoyloxiamino-androst-5ene **125**(**a**) showed the highest growth inhibition on IGROV (ovarian cancer cells) with a GI_50_ of 0.937 µg/mL. The structure of androstene oxime-nitrogen mustard hybrids is shown in Figure 119 and the in vitro GI_50_ (µg/mL) of compounds (**125**(**a**-**b**),**126**(**a**-**b**)) against cancer cell lines is shown in Table 106 [197].

### 3.21. Pyrazole-Based Hybrids

**Figure 120:**
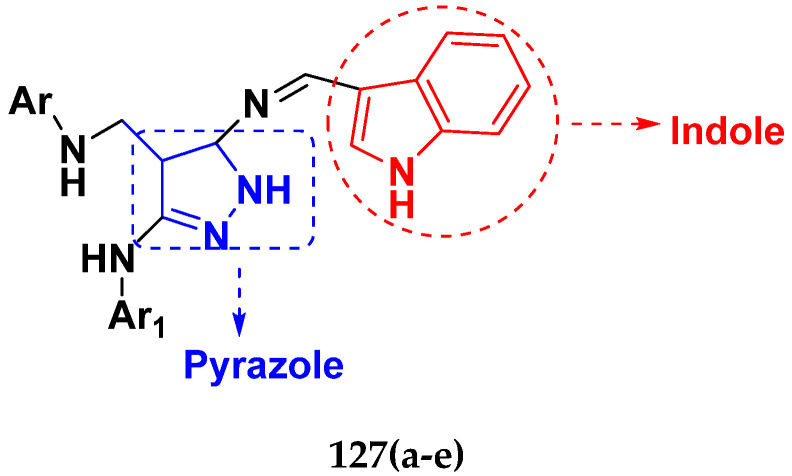
Structure of pyrazole-based indole derivatives **127**(**a**–**e**).

Hassan et al. (2021) synthesized indole-pyrazole hybrids as anticancer agents. The newly synthesized hybrids were screened for their cytotoxicity activities in vitro against four human cancer types, i.e., colorectal (HCT-116), breast carcinoma (MCF-7), liver carcinoma (HepG2) and lung carcinoma (A549). Among all indole-pyrazole hybrids, **126a** and **126b** showed excellent anticancer activity against the HepG2 cancer cell line with an IC_50_ of 6.1 and 7.9 µM, respectively. The structure of the pyrazole-based indole hybrid is given in Figure 120 and the in vitro IC_50_ of compounds **127**(**a**–**e**) against cancer cell lines are shown in Table 107 [198].

**Figure 121:**
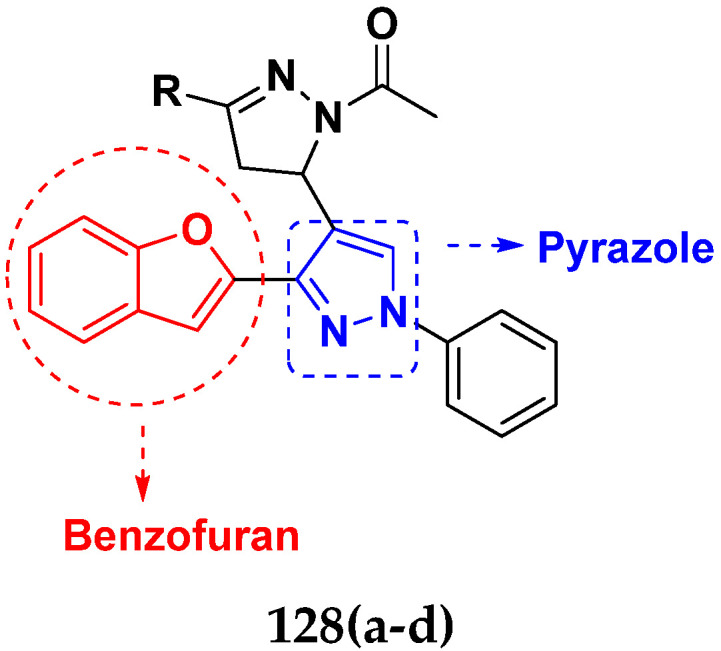
Structure of benzofuran-pyrazole-based derivatives **128**(**a**–**d**).

Somaia et al. (2015) synthesized benzofuran-pyrazole-based hybrids as anticancer drugs. The newly synthesized compounds showed remarkable growth inhibitory activity against leukemia (CCRF-CEM), A549 (lung carcinoma), and HCT-116 (colorectal carcinoma) cells. Compound **128c** showed good src inhibition activity at 10 µM and was found to be most potent compound. The structure of benzofuran-pyrazole hybrids is given in Figure 121 and in vitro IC_50_ of compounds **128**(**a**–**d**) against cancer cell lines is shown in Table 108 [199].

**Figure 122:**
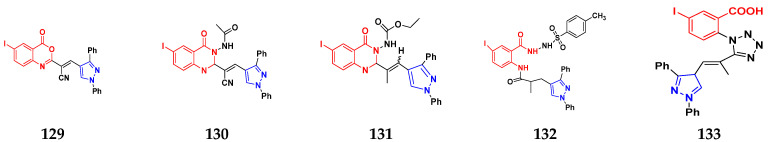
Structure of quinazoline-pyrazole-based derivatives (**129**–**131**) and pyrazole derivative (**132**–**133**).

Abdelaal et al. (2021) synthesized quinazoline-pyrazole hybrids as potential antiproliferative agents. The newly synthesized compounds showed activity against hepatocellular carcinoma (liver) HEPG2, mammary gland (breast) MCF-7 and colon cancer HCT-116 cells. The structure of quinazoline-pyrazole hybrids is given in Figure 122 and the in vitro IC_50_ of compounds (**129**–**133**) against cancer cell lines is shown in Table 109 [200].

**Figure 123:**
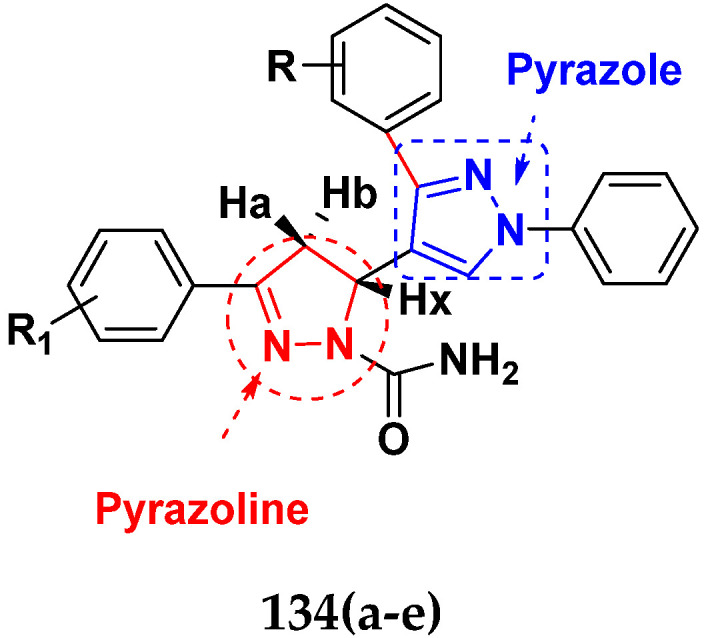
Structure of pyrazoline-pyrazole-based derivatives **134**(**a**–**e**).

Washim Akhtar et al. (2021) synthesized 15 novel pyrazoline-pyrazole hybrids, all of which showed activity in the MTT growth inhibition assay against five cancer cells lines: MCF-7, A549, SiHa, COLO205 and HePG2 cells. Compound **134** (**b**) was found to active against A549, SiHa, COLO205 and HePG2 cell lines with IC_50_ values of 4.94, 4.54, 4.98 and 2.09 µM, respectively, and was non-toxic against normal cells. The structure of pyrazoline-pyrazole hybrids is given in Figure 123 and in vitro IC_50_ of compounds **134**(**a**–**e**) against cancer cell lines is shown in Table 110 [201].

**Figure 124:**
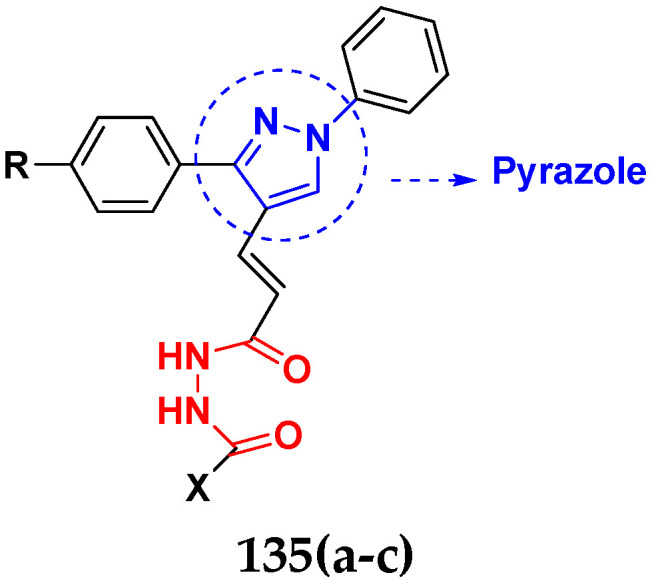
Structure of pyrazole acrylic acid-based oxadiazole derivatives **135**(**a**–**c**).

Garima Verma et al. (2018) synthesized pyrazole acrylic acid-based oxadiazole and amide hybrids as anticancer and antimalarial agents. The anticancer activity of newly synthesized compounds was measured using the sulforhodamine B assay. Compounds 135(**a**–**c**) demonstrated promising results against all tested cell lines. The non-cyclized compounds (amide derivatives) favored anticancer activity. The structure of pyrazole acrylic acid based oxadiazole hybrids is shown in Figure 124 and the in vitro IC_50_ of compounds **135**(**a**–**c**) against cancer cell lines is shown in Table 111 [202].

### 3.22. Pyridine-Based Hybrids

**Figure 125:**
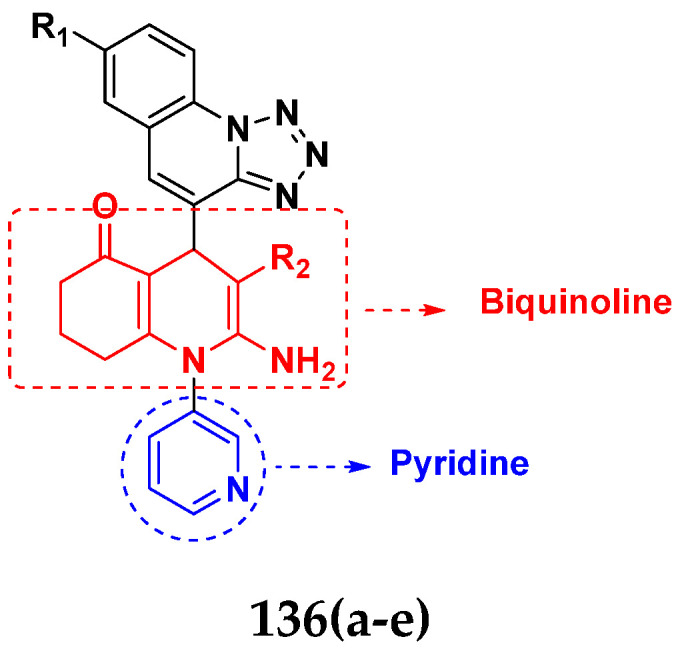
Structure of biquinoline-pyridine hybrid derivative **136**(**a**–**e**).

Chetan B. Sangani et al. (2014) synthesized biquinoline-pyridine hybrids as potential EGFR and HER-2 kinase inhibitors by base catalyzed cyclocondensation through one potent multicomponent reaction. All the synthesized compounds were tested against A549 (adenocarcinomic human alveolar basal epithelial) and Hep G2 (liver cancer) cell lines. Enzyme inhibitory activity was measured against HER-2. Compound **136**(**d**) was found to be most potent compound. The structure of biquinoline-pyridine hybrids is given in Figure 125 and the in vitro IC_50_ of compounds **136**(**a**–**e**) against cancer cell lines is shown in Table 112 [203].

**Figure 126:**
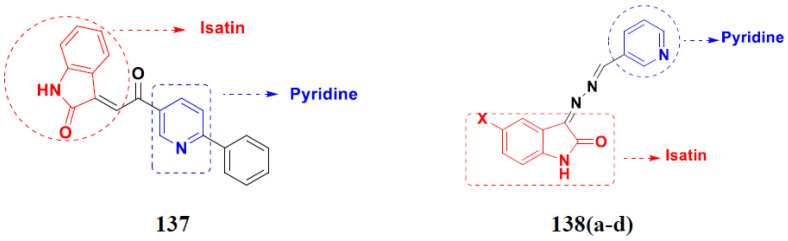
Structure of isatin-pyridine derivative [**137**,**138**(**a**–**d**)].

W.M. Eldehna et al. (2015) synthesized isatin-pyridine hybrids as potential antiproliferative agents. They showed antiproliferative activity against hepatocellular carcinoma (HEPG2), lung cancer (A549) and breast cancer (MCF-7) cell lines. Compound **137** showed the most potent activity against HEPG2 with an IC_50_ of 2.5 µM, while compound **138**(**c**) was the most potent compound against A549 and MCF-7 cell line with IC_50_ values of 10.8 and 6.3 µM, respectively. The structure of isatin-pyridine hybrids is shown in Figure 126 and the in vitro IC_50_ of compounds [**137**,**138(a–d)**] against cancer cell lines is shown in Table 113 [173].

**Figure 127:**
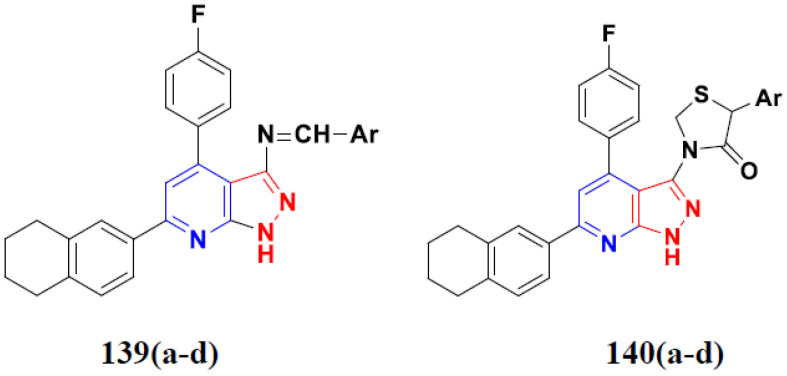
Structure of pyrazolo [3,4] pyridine derivatives [**139**(**a**–**d**),**140**(**a**–**d**)].

E.K. Hamza et al. (2020) synthesized pyrazolo [3,4] pyridine hybrids as potential anticancer agents. The activity of the newly synthesized compounds was evaluated in vitro against two human cancer cell lines (HCT116 and MCF-7). Compounds **139**(**a**–**d**) showed activity against HCT116 and compounds **140**(**a**–**d**) showed activity against MCF-7 cancer cell lines. The structure of pyrazolo [3,4] pyridine hybrids is shown in Figure 127 and the in vitro IC_50_ (µM) of compounds [**139**(**a**–**d**),**140**(**a**–**d**)] against cancer cell lines is shown in Table 114 [204].

## 4. Conclusions

For a very long time, medicinal chemists from all over the world have been trying to develop new and effective cancer treatments. Combination therapy and hybrid chemotherapeutics haves become more common, because a complex disease such as cancer cannot be properly treated with a single drug. This study presents rational approaches behind the design of anticancer agents employing molecular hybridization. This method has potential because it combines two moieties to create new molecular scaffolds. Molecular hybridization offers a wide range of applications since it can produce compounds with distinct and/or multiple modes of action and minimal side effects. The few examples included in this article are not intended to be an exhaustive collection of anticancer hybrids, but to provide a quick explanation of the idea and its potential uses for researchers working in this field.

## Figures and Tables

**Table 1:** In vitro cytotoxic activities of hybrid compounds **1**(**b**–**e**).

Compound No.	R_1_	R_2_	R_3_	R_4_	n	EGFR (IC_50_ nM)	HT-29 (IC_50_ µM)	
							Normoxia	Hypoxia
**1b**	Cl	F	NO_2_	H	5	0.32	12.89	9.81
**1c**	Br	H	NO_2_	H	2	0.66	4.48	4.01
**1d**	ethynyl	H	NO_2_	H	3	0.56	10.08	5.96
**1e**	ethynyl	H	NO_2_	H	5	0.50	2.93	3.46
**Gefitinib**						0.45	3.63	5.21

**Table 2:** In vitro cytotoxic activities of hybrid compounds **2**(**b**–**e**).

Compound No.	R_1_	R_2_	R_3_	EGFR (IC_50_ nM)	α-Glucosidase (IC_50_ µM)
**2b**	3-Cl, 4-(3-fluorobenzyloxy)	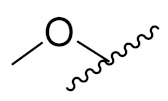	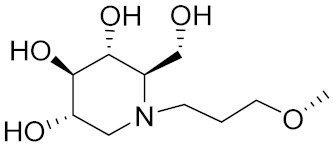	4.53	0.14
**2c**	3-ethynyl	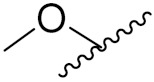	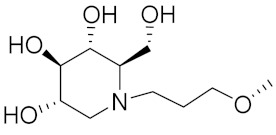	4.87	0.09
**2d**	3-ethynyl	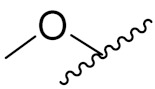	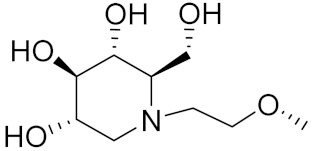	ND *	6.25
**2e**	3-Cl, 4-F	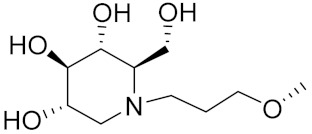	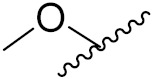	10.71	4.34
**Gefitinib**				3.32	≥100

* Not determined.

**Table 3:** In vitro cytotoxic activities of hybrid compounds **3**(**b**–**e**).

Compound No.	R_1_	R_2_	X	EGFR (IC_50_ nM)	VEGFR-2 (IC_50_ nM)
**3b**	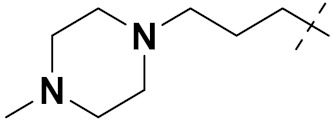	m-Cl, p-F	Cl	14	14
**3c**	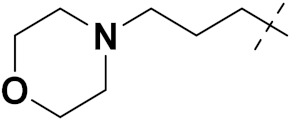	m-CH_3_, p-CH_3_	Cl	78	51
**3d**	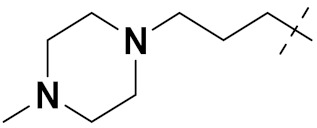	o-CH_3_	Cl	15	178
**3e**	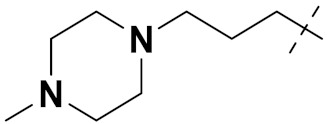	H	Cl	14	261
**vandetanib**				11	15

**Table 4:** In vitro cytotoxic activities of hybrid compounds **4** (**b**–**e**).

Compound No.	Ar.	MCF-7 (GI50 μM)	PI3K (IC_50_ μM)		
			A	β	γ
**4b**	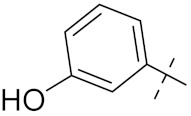	15	5.3	16	2.3
**4c**	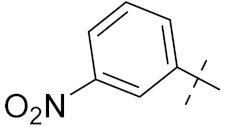	12	22.2	1.9	22.2
**4d**	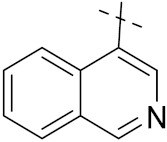	12	133	56	5.9
**4e**	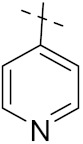	32	14.2	0.5	14.2

**Table 5:** In vitro cytotoxic activities of hybrid compounds **5**(**b**–**e**).

Compounds No.	R_1_	R_2_	X	A549 (µM)	BT549 (µM)	HCT-116 (µM)	MCF-7 (µM)	SK-HEP-1 (µM)	SNU638 (µM)
**5b**	3-COOCH	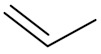	CH	1.10	1.08	0.40	10.1	2.40	1.12
**5c**	3-COOCH_3_-4-Cl	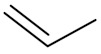	CH	2.13	2.36	3.13	1.43	7.06	2.20
**5d**	4-OCH_3_	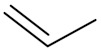	CH	3.58	1.88	3.49	1.79	1.61	3.97
**5e**	2,4-diF	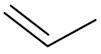	CH	4.71	2.48	4.01	1.61	2.49	2.05
**Gefitinib**				8.27	6.56	5.98	26.7	10.1	7.56
**Dactolisib**				0.62	0.74	0.84	1.33	1.82	1.24

**Table 6:** In vitro cytotoxic activities of hybrid compounds **6**(**b**–**e**).

Compound No.	R1	R2	HCT-116 (µM)	SK-HEP1 (µM)	MDA-MB-231 (µM)	SNU638 (µM)	A549 (µM)	MCF-7 (µM)
**6b**	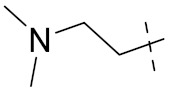	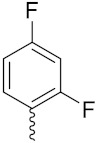	1.44	4.72	0.71	0.62	0.94	1.02
**6c**	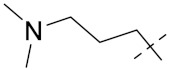	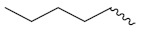	0.49	0.86	0.88	1.26	3.52	4.73
**6d**	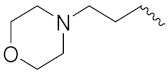	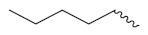	0.59	0.44	0.42	0.61	1.56	10.8
**6e**	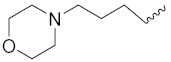	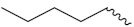	1.74	1.14	2.58	0.98	3.14	4.59
**BEZ235**			0.84	1.82	0.18	1.24	0.62	1.33

**Table 7:** In vitro cytotoxic activities of hybrid compounds **8**(**b**–**e**).

Compound No.	R_1_	BRD4 K_d_ (nM)	MV4-11, IC_50_ (µM)
**8b**	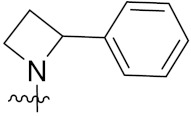	480	4.88
**8c**	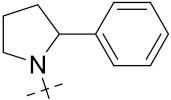	250	2.54
**8d**	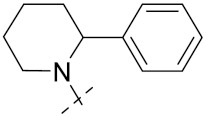	60	5.07
**8e**	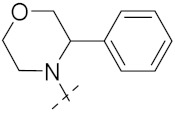	28	1.83
**BET760**	-	37	0.80

**Table 8:** Quinazoline-based compounds approved/under clinical trial with their current status.

Company Name	Compound Name	Drug Target	Type of Cancer	Status	References
AstraZeneca	Vandetanib	Kinase inhibitor	Medullary thyroid cancer	Approved	[33]
Boehringer Ingelheim	Afatinib	Tyrosine kinase	Non-small cell lung Carcinoma	Approved	[34]
Pfizer	Dacomitinib	EGFR inhibitor	Non-small cell lung carcinoma	Approved	[10]
AstraZeneca and Teva	Gefitinib	EGFR inhibitor	Breast and Lung cancer	Approved	[35]
Roche Pharmaceuticals	Erlotinib	EGFR inhibitor	pancreatic cancer and non-small cell lung cancer	Approved	[36]
GlaxoSmith Kline (GSK)	Lapatinib	Dual tyrosine kinase inhibitor	solid tumors and Breast cancer	Approved	[37]
AstraZeneca	Sapitinib (AZD 8931)	Erb8 receptor tyrosine kinase	Breast cancer and metastatic cancer	Clinical trials	[10]
Array Biopharma	Tucatinib (ARRY 380)	Kinase inhibitor	Breast cancer	Approved	[10]
Selleck chemicals	Barasertib (AZD 1152)	Aurora Kinase	Tumor lymphoma, solid tumors and myeloid leukemia	Clinical trials	[38]
Spectrum Pharmaceuticals	Poziotinib	Tyrosine kinase	Breast cancer	Clinical trials	[10]
AstraZeneca	AZD 3759	EGFR antagonist	Non-small cell lung Cancer	Clinical trials	[10]
Curis Inc.	CUDC-101	By inhibiting Histone deacetylase, EGFR and HER2	Advanced /Liver/Neck/Gastric/Head/non-small cell lung cancer and Breast	Clinical trials	[39]
Beta-Phama	Icotinib	EGFR-TK1 inhibitor	Non-small cell lung cancer	Approved	[40]

**Table 9:** In vitro cytotoxicity (IC_50_) of hybrid compounds **9**(**b**–**e**).

Compound No.	R	U937 _(_µM)	PC-3 _(_µM)	A549 _(_µM)	ES-2 _(_µM)	MDA-MB-231 _(_µM)	HCT116 _(_µM)
**9b**	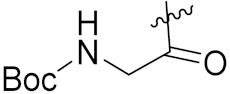	3.1	10.5	11.8	29.2	7.2	6.0
**9c**	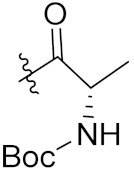	2.2	10.4	4.2	25.1	4.5	3.8
**9d**	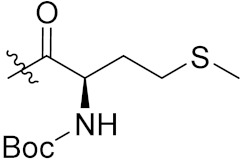	2.2	5.8	1.6	4.4	6.8	5.9
**9e**	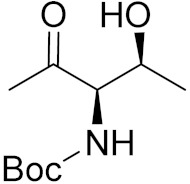	2.7	5.4	7.0	8.9	7.2	2.4
**SAHA**	-	2.3	9.9	3.8	12.7	5.6	6.0

**Table 10:** In vitro cytotoxic activities of hybrid compounds **10**(**b**–**e**).

Compound No.	R	U937 (µM)	K562 (µM)	HEL (µM)	KG1 (µM)	HL60 (µM)	MDA-MB-231 (µM)	PC-3 (µM)	MCF-7 (µM)	HCT116 (µM)	A549 (µM)
**10b**	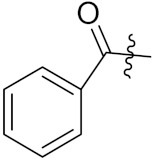	0.33	0.79	0.20	0.39	2.11	0.24	0.33	3.47	0.37	3.39
**10c**	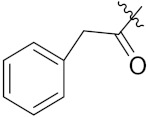	0.32	0.68	0.27	0.72	1.59	0.41	0.53	2.95	0.57	3.91
**10d**	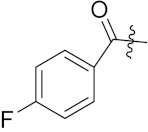	0.18	1.01	0.19	0.24	1.04	0.27	0.51	2.7	0.37	2.96
**10e**	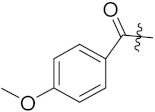	0.34	0.89	0.16	0.47	1.68	0.15	0.29	2.32	0.22	3.27
**SAHA**		1.45	3.24	0.49	1.59	4.26	1.72	3.57	3.78	2.81	3.9

**Table 11:** In vitro cytotoxic activities of hybrid compounds **11**(**b**–**e**).

Compound No.	R_1_	R_2_	R_3_	HeLa Nuclear HDAC (nM)
**11b**	H	H	4′-(N-3-hydroxyacrylamide)	2.8
**11c**	CH_3_	H	4′-(N-3-hydroxyacrylamide)	3.3
**11d**	CH_2_CH_3_	H	4′-(N-3-hydroxyacrylamide)	3.4
**11e**	H	CH_3_	4′-(N-3-hydroxyacrylamide)	47.4
**LBH589.HCl**				7.5

**Table 12:** In vitro cytotoxic activities of indole-triazole based hybrid compounds **12**(**b**–**e**).

Compound No.	R_1_	R_2_	K562 (Upto %)	MDA-MB 231 (µM)	LNCaP (µM)
**12b**	H	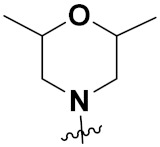	88	2	32
**12c**	H	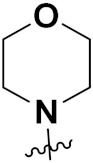	87	28	38
**12d**	F	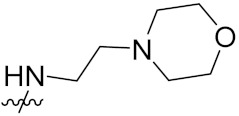	64	58	24
**12e**	F	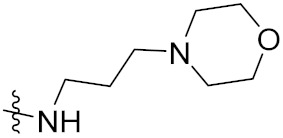	67	68	35

**Table 13:** In vitro cytotoxic activities of indole-pyrimidine based hybrid compounds **13**(**b**–**e**).

Compound No.	R	PIM (µM)		
		PIM1	PIM2	PIM3
**13b**	Me_2_N(CH_2_)_2_O	0.30	1.40	0.50
**13c**	Et_2_N(CH_2_)_2_O	0.14	0.84	0.27
**13d**	Et_2_N(CH_2_)_3_O	0.11	0.38	0.081
**13e**	Et_2_N(CH_2_)_3_NH	0.067	3.16	0.61

**Table 14:** In vitro cytotoxic activities of indole-chalcone based hybrid compounds **14**(**b**–**e**).

Compound No.	R1	R2	R3	A549 (µg/mL)	MCF7 (µg/mL)	SKOV3 (µg/mL)	NIH3T3 (µg/mL)
**14b**	C_2_H_5_	Br	Me	4.9	50.1	68.8	52.0
**14c**	H	Br	n-Bu	8.0	54.0	74.1	141.3
**14d**	Me	OMe	Me	5.1	36.2	53.0	_
**14e**	H	Br	Me	27.9	28.0	52.0	_
**Etoposide**				7.8	9.9	8.5	118.0

**Table 15:** In vitro cytotoxic activities of indole-pyrole based hybrid compounds **15**(**b**–**e**).

Compound No.	R_1_	R_2_	R_3_	HL-60 (µM)	SMMC-7721 (µM)	A-549 (µM)	MCF-7 (µM)	SW480 (µM)
**15b**	Bn	Benzimidazole	2-Bromobenzyl	1.21	4.69	6.76	2.23	6.35
**15c**	Bn	Benzimidazole	4-Methylbenzyl	1.20	4.98	6.23	2.72	6.57
**15d**	Bn	5,6-Dimethyl-benzimidazole	2-Naphthylmethyl	1.21	2.27	4.80	1.68	1.76
**15e**	Me	5,6-Dimethyl-benzimidazole	2-Naphthylmethyl	1.35	4.03	6.18	1.84	4.5
**DPP**	-	-	-	1.16	8.08	7.10	10.45	8.88

**Table 16:** In vitro cytotoxic activities of indole-chalcone based hybrid compounds **16**(**b**–**e**).

Compound No.	R_1_	R_2_	R_3_	R_4_	R_5_	R_6_	A549 (µM)		PC3 (µM)		PaCa2 (µM)	
							24 h	48 h	24 h	48 h	24 h	48 h
**16b**	H	H	H	OCH_3_	H	H	9.6	5.8	33.3	12.5	27.5	>50
**16c**	H	OCH_3_	H	H	H	H	6.4	7.5	>50	12.0	13.5	>50
**16d**	H	OCH_3_	OCH_3_	H	H	H	3.7	5.5	31.1	37.1	>50	>50
**16e**							4.9	3.0	17.2	8.1	24.0	>50
**Mitomycin C**							0.45					

**Table 17:** In vitro cytotoxic activities of indole-ospemifene-triazole based hybrid compounds **17**(**b**–**e**).

Compound No.	R	MCF-7 (µM)	MDA-MB-231 (µM)
**17b**	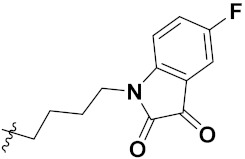	≥100	71.40
**17c**	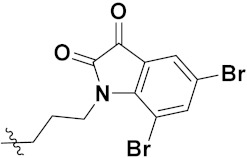	16.50	≥100
**17d**	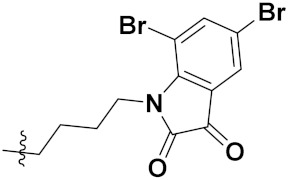	10.99	71.40
**17e**	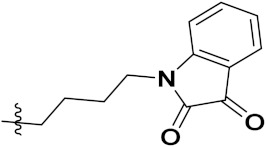	≥100	≥100
**Ospemifene**		55	50
**Tamoxifen**		3.5	≥100
**Plumbagin**		75	4.4

**Table 18:** In vitro cytotoxic activities of indole-isatin-triazole based hybrid compounds **18**(**b**–**c**).

Compound No.	R	MCF-7 (µM)	MDA-MB-231 (µM)
**18b**	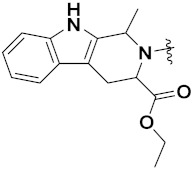	50	>100
**18c**	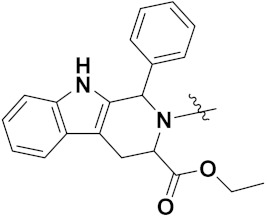	>100	>100
**Plumbagin**		3.5	4.4
**Peganumine A**		38.5	Not observed
**Tamoxifen**		50	75

**Table 19:** Current status of indole-based hybrids that are approved or/-under clinical trials.

Company Name	Compound Name	Drug Target	Type of Cancer	Status	Reference
AstraZeneca	Cediranib	VEGFR tyrosine kinases	Glioblastoma	Approved	[53]
Chia-tai Tianqing Pharmaceutical Co.	AL 3818 (Anlotinib)	Tyrosine kinase	Synovial sarcoma, Advanced alveolar soft part sarcoma	Clinical trials	[54]
Janssen pharmaceuticals	Quisinostat	HDAC inhibitor	Multiple myeloma	Approved	[55]
Novartis	Panobinostat (LBH-589)	Non-selective HDAC inhibitor	Multiple myeloma	Approved	[56]
Selleck	Dacinostat (LAQ824)	Histone deacetylase inhibitor	Breast and Prostate cancer	Approved	[57]

**Table 20:** In vitro cytotoxic activities of carbazole-imidazole based hybrid compounds **19**(**b**–**e**).

Compound No.	n	R	R’	HL-60 (µM)	SMMC-7721 (µM)	A549 (µM)	MCF-7 (µM)	SW480 (µM)
**19b**	2	Benzimidazole	-	3.11	3.21	12.36	5.06	18.25
**19c**	2	imidazole	4-methylbenzyl	0.84	5.74	3.92	2.24	9.56
**19d**	2	benzimidazole	2-bromobenzyl	0.71	3.66	3.58	2.14	3.08
**19e**	3	benzimidazole	4-methylbenzyl	0.57	2.55	2.65	2.82	3.19
**DDP**				1.32	6.24	11.83	15.17	12.95

**Table 21:** Current status of carbazole-based hybrid drugs that are approved/or under clinical trials.

Company Name	Compound Name	Drug Target	Type of Cancer	Status	References
Novartis Pharmaceutical Corporation	Midostaurin	Kinase inhibitor	Advanced systemic mastocytosis, myelodysplastic syndrome	Approved	[67]
Chugai Pharmaceuticals Co.	Alectinib	Tyrosine kinase	Non-small cell lung cancer	Approved	[68]
Schwarz Pharma	CEP-2563	Tyrosine kinase	Solid tumors	Clinical trials	[10]
Cayman Chemicals	UCN-01	Tyrosine kinase	Pancreatic, malignant melanoma, ovarian Cancer and small cell lung	Clinical trials	[69]
Helsinn Healthcare	Becatecarin	Topoisomerase-I	Leukemia and gastric cancer	Clinical Trials	[10]
Pfizer Pharmaceutical Co.	Edotecarin	Topoisomerase-I	Oesophageal cancer and solid tumors	Clinical trials	[10]

**Table 22:** In vitro cytotoxic activities of sulfonamide-thiazole fused pyrimidine hybrid compounds **29**(**b**–**e**).

Compound No.	R	R_1_	DNA Displacement Assay (µg/mL)	DNA Binding Affinity
**29b**	4-H	4-Br	74	High
**29c**	4-H	4-NO_2_	81	weak
**29d**	4-H	4-OCH_3_	81	Moderate
**29e**	4-H	3,4-diOMe	62	High
**Ethidium bromide**	-	-	1.4	-

**Table 23:** In vitro cytotoxic activities of tri-substituted pyrimidine hybrid compounds **30**(**b**–**e**).

Compound No.	R_1_	R_2_	R_3_	CDK9T1 (µM)	CDK1B (µM)	CDK2A (µM)	CDK7H (µM)	HCT-116 (µM)	MCF-7 (µM)
**30b**	NH_2_	F	m-SO_2_NH_2_	3	7	3	252	0.05	0.41
**30c**	NHMe	CN	m-SO_2_Me	5	19	43	110	0.20	0.43
**30d**	NHMe	F	m-SO_2_NH(CH_2_)_2_OCH_3_	3	10	6	30	0.30	0.72
**30e**	NHMe	CN	p-CO-N-(1-methylpiperidin-4-yl)	8	43	32	304	0.18	0.5

**Table 24:** In vitro cytotoxic activities of pyrimidine-triazole hybrid compounds **31**(**b**–**e**).

Compound No.	Ar	R	A-549 (IC_50_ (µM)	PC-3
**31b**	4-MeC_6_H_4_	COCH_3_	19.33	16.92
**31c**	C_6_H_5_	COOC_2_H_5_	26.64	33.56
**31d**	4-SO_2_NH_2_C_6_H_4_	COOC_2_H_5_	16.42	7.15
**31d**	4-ClC_6_H_4_	COCH_3_	30.56	22.90
**5-FU**			4.21	12.00

**Table 25:** In vitro cytotoxic activities of pyrimidine-triazole hybrid compounds **32**(**b**–**e**).

Compound No.	R1	R2	U_937_ (µg/mL)	THP-1 (µg/mL)	Colo205 (µg/mL)
**32b**	H	CH_3_-(CH_2_)_8_-CH_2_-	8.16	16.91	19.25
**32c**	CH_3_-(CH_2_)_4_-CH_2_-	6F_13_-CH_2_-CH_2_-	7.56	-	132.42
**32c**	(CH_3_)_2_CH-	8F_17_-CH_2_-CH_2_-	8.35	142.23	-
**32d**	C_2_H_5_	CH_3_-(CH_2_)_8_-CH_2_-	17.83	82.65	-
**Etoposide**			17.94	2.16	7.24

**Table 26:** In vitro cytotoxic activities of pyrimidine-pyrazole based hybrid compounds **33**(**b**–**e**).

Compound No.	R	A549 (µM)	MCF7 (µM)	DU145 (µM)	HeLa (µM)
**33b**	CH_3_CH_2_OC(O)CH_2_-	16.3	12.4	18.2	9.8
**33c**	CH_3_(CH_2_)_6_CH_2_-	5.7	24.7	6.3	22.7
**33d**	CF_3_(CF_2_)_7_CH_2_CH_2_-	33.7	-	37.7	-
**33e**	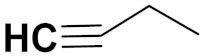	4.1	-	4.7	-
**5-FU**	-	1.3	1.4	1.5	1.3

**Table 27:** In vitro cytotoxic activities of pyrimidine-pyrazole based hybrid compounds **34**(**b**–**e**).

Compound No.	R_1_	R_2_	NCI-H226 (µM)	NPC-TW01 (µM)	Jurkat
**34b**	Ph	p-Me-Ph	35	49	48
**34c**	2-Quinolinyl	p-Cl-Ph	39	35	69
**34d**	2-Quinolinyl	p-OMe-Ph	37	36	>100
**34e**	Ph	p-Cl-Ph	18	23	36
**N0-(4-formyl-1,3-diphenyl-1H-pyrazol-5-yl)-N,N-dimethyl-methanimidamide**			9.3	31.4	23.5

**Table 28:** FDA approved pyrimidine based hybrids.

Company Name	Compound Name	Drug Target	Type of Cancer	Status	Reference
Novartis	Ceritinib	Abnormal ALK-gene	Non-Small cell lung Cancer	Approved	[85]
Pfizer Pharmaceutical company	Palbociclib	CDK4/6 inhibitor	Breast cancer	Approved	[86]
AbbVie Pharmaceuticals	Ibrutinib	Tyrosine kinase	Mantle cell lymphoma	Approved	[87,88]

**Table 29:** In vitro cytotoxic activities of quinoline-pyrimidine based hybrid compounds **35**.

Compounds No.	BALB/3T3 (µM)	A549 (µM)	MCF-7 (µM)	LoVo (µM)	KB (µM)
**35**	30.04	3.24	0.81	9.38	0.87
**Doxorubicin**	1.08	0.33	0.44	0.11	0.84
**DiMIQ**	5.77	2.19	1.54	0.20	1.14

**Table 30:** In vitro cytotoxic activities of tetrahydro-pyrimido-quinoline based hybrid compound **36**.

Compound No.	HCT116 (µM)	MCF7 (µM)
**36**	16.33	27.26
**Imatinib**	34.40	-
**Tamoxifien**	--	34.30

**Table 31:** In vitro cytotoxic activities of quinoline-indole based hybrid compounds **38**(**b**–**e**).

Compound No.	R_1_	R_2_	KB (µM)	A-549 (µM)	MCF-7 (µM)	Hs294T (µM)	BALB/3T3 (µM)
**38b**	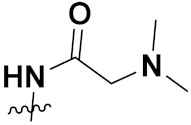	H	0.15	0.81	0.79	0.64	0.67
**38c**	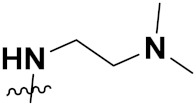	H	0.36	0.29	0.99	0.72	0.60
**38d**	H	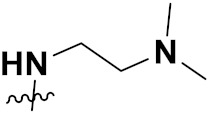	0.64	0.17	0.47	0.35	0.34
**38e**	H	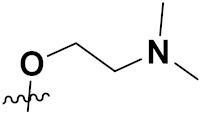	0.08	0.19	0.66	0.76	0.57
**DIMIQ**			1.14	2.19	1.50	9.70	5.70

**Table 32:** In vitro cytotoxic activities of quinoline based ursolic acid hybrids compounds **39**(**b**–**e**).

Compound No.	R_1_	R_2_	MDA-MB-231(µM)	HeLa (µM)	SMMC-7721(µM)	QSG-7701(µM)
**39b**	H	CH_3_	1.84	1.18	17.48	40.59
**39c**	OCH_3_	CH_3_	1.42	0.83	17.65	45.20
**39d**	F	CH_3_	1.16	0.99	19.41	>50
**39e**	H	n-C_4_H_9_	>50	>50	>50	Not tested
**Etoposide**	-	-	5.26	2.98	3.48	28.75

**Table 33:** In vitro cytotoxic activities of quinoline based piperazine hybrids compounds **40**(**b**–**e**).

Compound No.	R_1_	MB231 (GI_50_ µM)	MB468 (GI_50_ µM)	MCF-7 (GI_50_ µM)	184B5 (GI_50_ µM)	MCF10A (GI_50_ µM)
**40b**	2,4-Dinitrophenyl	24.3	19.2	10.8	37.8	35.4
**40c**	3-Nitrophenyl	32.2	18.6	9.4	17.7	15.4
**40d**	2,4-Dichlorophenyl	20.3	18.6	16.7	20.4	15.6
**40e**	Biphenyl	27.2	20.5	14.8	19.1	15.5
**Chloroquine**	-	22.5	28.6	38.4	76.1	81.26
**Cisplatin**	-	23.7	31.0	25.8	25.5	51.51

**Table 34:** Quinoline based FDA approved hybrids.

Company Name	Compound Name	Drug Target	Type of Cancer	Status	Reference
Eisai Co.	Lenvatinib	Kinase inhibitor	Thyroid cancer	Approved	[99]
Exelixis Inc.	Cabozantinib	Tyrosine-kinase	Thyroid cancer and renal carcinoma	Approved	[68]
Wyeth and Pfizer	Bosutinib	BCR and Src tyrosine kinase	myelogenous leukemia	Approved	[100]

**Table 35:** In vitro cytotoxic activities of quinone based chalcone hybrid compounds **42**(**b**–**e**).

Compound No.	R	HeLa (µM)	LS174 (µM)	A549 (µM)	MRC-5 (µM)
**42b**	H	2.41	4.56	26.20	33.57
**42c**	2-CH_3_	2.36	3.13	29.05	41.87
**42d**	3-CH_3_	2.45	11.79	33.70	52.00
**42e**	4-CH_3_	2.64	22.63	24.15	38.49
**cisplatin**	-	2.10	5.54	11.92	14.21

**Table 36:** In vitro cytotoxic activities of quinone based pyran hybrid compounds **43**(**b**–**e**).

Compound No.	R	KB (µg/mL)	KB/VCR (µg/mL)	A549 (µg/mL)	HL60 (µg/mL)
**43b**	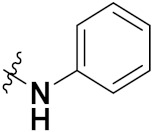	4.31	2.21	6.58	4.45
**43c**	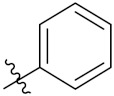	>8.00	>8.00	>8.00	>8.00
**43d**	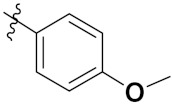	>8.60	8.52	4.49	4.46
**Vincristine**	-	0.46	0.26	12.09	-
**Adriamycin I**	-	-	-	-	0.02

**Table 37:** In vitro cytotoxic activities of imidazole based benzofuran hybrid compounds **44**(**a**–**e**).

Compound No.	R	X	SMMC-7721 (µM)	SW480 (µM)	MCF-7 (µM)	A549 (µM)	HL-60 (µM)
**44a**	2-Bromobenzyl	Br	4.38	12.71	14.29	9.77	1.97
**44b**	Phenacyl	Br	3.71	10.34	11.90	12.94	2.61
**44c**	4-Bromophenacyl	Br	3.39	2.85	2.84	8.46	3.15
**44d**	Naphthyl acyl	Br	1.65	3.38	5.87	10.93	2.49
**44e**	2′-Phenyl-phenacyl	Br	3.31	6.93	6.90	6.79	2.70
**DPP**	-	-	8.86	15.92	16.65	11.68	1.81

**Table 38:** In vitro cytotoxic activities of imidazole based benzofuran hybrid compounds **45**(**a**–**e**).

Compound No.	R_1_	R_2_	SMMC-7721 (µM)	SW480 (µM)	MCF-7 (µM)	A549 (µM)	HL-60 (µM)
**45a**	Benzimidazole	2-Bromobenzyl	2.10	5.56	4.78	3.34	0.64
**45b**	2-Ethylimidazole	4-Hydroxyphenacyl	11.81	5.69	3.17	12.90	0.58
**45c**	2-Ethylimidazole	4-Bromophenacyl	6.07	3.58	2.89	12.76	0.72
**45d**	2-Ethylimidazole	Naphthylacyl	2.30	3.14	3.03	5.35	0.61
**45e**	2-Ethylimidazole	2-Bromobenzyl	0.52	0.47	0.51	0.55	0.08
**DPP**			1.69	12.49	14.09	20.82	18.85

**Table 39:** In vitro cytotoxic activities of imidazole based triazole hybrid compounds **46**(**a**–**e**).

Compound No.	R	Caco2 (µM)	HCT116 (µM)	HeLa (µM)	MCF-7 (µM)
**46a**	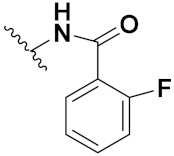	6.31	12.04	7.91	3.80
**46b**	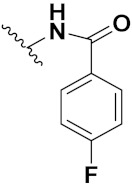	8.45	18.32	9.45	4.45
**46c**	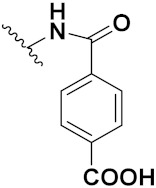	4.67	16.78	6.87	0.38
**46d**	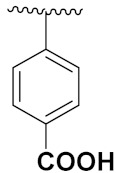	5.22	18.70	8.42	3.87
**46e**	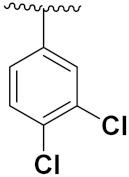	10.87	30.98	20.34	15.56
**Doxorubicin**	-	5.17	5.64	1.25	0.65

**Table 40:** In vitro cytotoxic activities of imidazole-based artemisinin hybrid compounds **47**(**a**–**e**).

Compound No.	R_1_	R_2_	R_3_	n	MCF-7 (µM)	A549 (µM)	HEPG-2 (µM)	MDA-MB-231 (µM)	LO2 (µM)
**47a**	H	CN	CN	1	10.75	12.36	25.59	>100	49.05
**47b**	H	NO_2_	H	2	12.86	20.95	39.30	>100	46.37
**47c**	H	H	Br	2	12.40	26.02	41.59	80.92	>100
**47d**	H	H	NO_2_	2	12.69	19.97	33.33	80.92	34.32
**47e**	H	NO_2_	H	3	9.78	16.53	21.25	85.20	45.74
**Andriamycin**	-	-	-		0.67	1.13	0.85	2.94	0.79

**Table 41:** In vitro cytotoxic activities of imidazole-based benzofuran hybrid compounds **48**(**a**–**e**).

Compounds No.	R_1_	R_2_	R_3_ (Imidazole)	SKOV-3 (µM)	HL-60 (µM)	MCF-7 (µM)
**48a**	H	H	Benzimidazole	9.5	8.4	11.8
**48b**	H	Allyl	Benzimidazole	7.9	>40	>40
**48c**	OMe	Allyl	Imidazole	36.2	>40	>40
**48d**	OMe	Allyl	Benzimidazole	9.3	>40	>40
**48e**	H	H	Imidazole	>40	>40	>40
**DDP**	-	-	-	8.9	5.5	13.0

**Table 42:** Imidazole based anticancer drugs with brand/company name, specific cancer types and targets.

Brand/Company Name	Compound Name	Drug Target	Type of Cancer	Status	References
AdisInsight-springer/Ligand pharmaceuticals	Acadesine	AMP-activated protein kinase	Acute lymphoblastic leukemia	Phase-III	[105]
Tasigna/ Novartis	Nilotinib	BCR-ABL	Leukemia	FDA approved	[114]
Hikma Pharmaceuticals	Dacarbazine	DNA synthesis	Melanoma; Lymphoma	FDA approved	[115]
Janssen Pharmaceutical	Tipifarnib	Farnesyltransferase inhibitors (FTIs)	Breast cancer	phase II trials	[116]
Treanda Astellas Pharma	Bendamustine hydrochloride	DNA synthesis	Leukemia; Lymphoma	FDA approved	[115]
Oforta/ Sanofi pharma	Fludarabine phosphate	DNA synthesis	Leukemia	FDA approved Discontinued	[115]
Nova Laboratories, Ltd.	Azathioprine prodrug of mercaptopurine	Thiopurine S-methyltransferase (TPMT)	Childhood acute lymphoblastic leukemia	FDA approved	[115]
Iclusig/ Otsuka Pharmaceutical	Ponatinib	BCR-ABL	Leukemia	FDA approved	[115]
Temodar/ Ranbaxy (UK)	Temozolomide	DNA synthesis	Brain cancer	FDA approved	[115]
Puri-nethol/ German Remedies Limited	Mercaptopurine	HPRT1	Leukemia	FDA approved	[115]
GlaxoSmithKline (GSK)	SB-431542	Activin receptor-like kinase (ALK) receptors	Childhood acute lymphoblastic leukemia	No clinical trials	[105]

**Table 43:** In vitro cytotoxic activities of organoselenium hybrid compounds **49**(**a**–**c**).

Compound No.	Structure	h	SW480 (µM)	HeLa (µM)	A549 (µM)	MCF-7 (µM)
**49a**	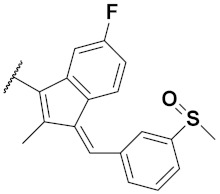	24	13.4	24.3	9.4	8.2
		48	11.4	15.1	11.3	6.4
		72	10.1	19.4	7.4	10.4
**49b**	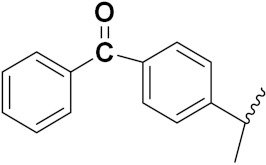	24	8.2	19.6	13.1	8.6
		48	7.4	17.5	18.4	9.3
		72	6.5	28.7	22.6	9.5
**49c**	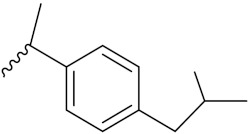	24	4.9	11.5	9.4	3.4
		48	3.3	17.4	15.2	4.3
		72	4.2	19.7	18.5	2.8
**Fluorouracil (5FU)**	-	24	15.3	20.6	25.3	8.5
		48	12.4	15.5	22.5	10.4
		72	13.1	12.7	17.3	12.6

**Table 44:** In vitro cytotoxic activities of selenium based quinone triazole hybrid compounds **50**(**a**–**e**).

Compound No.	R	HL-60 (µM)	HCT-116 (µM)	SF295 (µM)	NCIH-460 (µM)	PC3 (µM)	L929 (µM)
**50a**	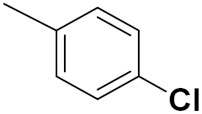	0.81	78.0	2.60	2.06	2.03	0.52
**50b**	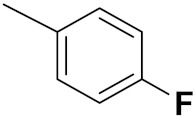	0.59	0.37	1.48	1.32	1.06	0.36
**50c**	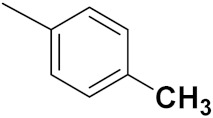	1.0	2.03	3.12	3.26	2.70	0.61
**50d**	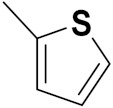	0.53	78.0	2.13	2.75	2.47	3.16
**50e**	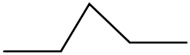	0.71	0.97	3.43	2.64	1.64	2.12
**DOXO**	-	0.02	0.21	0.41	0.15	0.76	1.72

**Table 45:** In vitro cytotoxic activities of selenium based anilino quinazoline hybrid compounds **51**(**a**–**e**).

Compound No.	R_1_	R_2_	R_3_	R_4_	RKO (nM)	HGPG2 (nM)	MCF7 (nM)	HELA (nM)	HCT116 (nM)	MGC803 (nM)
**51a**	H	H	-CN	Cl	3.39	5.24	9.98	2.09	4.97	3.54
**51b**	CH_3_	H	-CN	Cl	6.01	6.79	18.9	8.73	22.7	16.5
**51c**	Cl	H	-CN	Cl	3.42	6.78	10.6	6.78	9.17	9.67
**51d**	F	H	-Se-CH_3_	Cl	7.87	13.2	22.5	9.28	12.3	14.8
**51e**	H	H	-Se-CH_3_	-OCH_3_	7.85	8.92	22.7	9.15	11.4	4.78
**EP128495**	-	-	-	-	4.22	6.47	5.61	5.11	8.27	6.23

**Table 46:** In vitro cytotoxic activities of selenium based anilino quinazoline hybrid compounds **52**(**a**–**d**).

Comound No.	R	HeLa (nM)	HUH-7 (nM)	SKOV3 (nM)	A2780 (nM)
**52a**	OH	3.3	2.3	1.6	2.0
**52b**	F	2.0	6.7	8.5	7.9
**52c**	Cl	6.1	3.7	1.0	5.9
**52d**	I	9.2	17.1	9.4	8.2
**Paclitaxol**	-	2.1	3.2	2.2	3.2

**Table 47:** In vitro cytotoxic activities of selenium based diaryl imidazole hybrid compounds **53**(**a**–**e**).

Compound No.	R_1_	R_2_	A 2780 (nM)	IOSE 80(nM)
**53a**	OMe	Et	9.7	51.3
**53b**	OMe	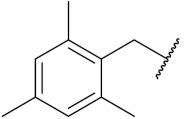	6.4	11.0
**53c**	F	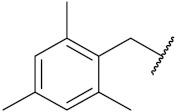	19.1	34.8
**53d**	F	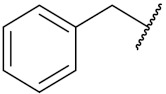	13.0	29.4
**53e**	Br	Et	21.1	43.7
**Ebselen**	-	-	25.4	55.4

**Table 48:** Selenium-based potent compounds with mode of action, specific cancer types and doses.

Compound Name	Mode of Action	Type of Cancer	Dose (Conc.) *	References
Methylseleninic Acid (MSA)	Apoptosis mediated by caspases, ER stress, UPR, mitochondrial dysfunction/signaling and PARP cleavage Anoikis, whereby cell detachment is a prerequisite for caspase activation and PARP cleavage	Breast, Colon Lung, Lymphoma Pancreatic	Medium to Low In pancreatic Very low-Low	[119]
Ebselen and corresponding Analogues	Not determined. Compounds have antioxidant activity	Breast, Liver, Promyelocytic Leukemia, Prostate	Medium-high	[119]
Benzoselenazole- stilbene hybrids	Apoptosis mediated by thioredoxin reductase inhibition and oxidative stress	Breast, Cervical, Liver, Lung	Very low-low	[119]

* Effective Dose in vitro (48–72 h IC_50_) low (0.1–2 µM) (1–20 µM) Medium (10–100 µM) High (100+ µM).

**Table 49:** In vitro cytotoxic activities of Platinum−acridine hybrid compounds **54**(**a**–**e**).

Compound No.	R	m	OVCAR-3 (µM)	MCF-7 (µM)	MDA-MB231 (µM)	PANC1 (µM)	NCI-H460 (µM)
**54a**	CH_3_	2	1.1	2.5	15	0.09	0.008
**54b**	CH_3_	3	33	11	37	4.4	0.052
**54c**	(CH_2_)(CO)OCH_3_	2	-	-	-	-	0.036
**54d**	(CH_2_)_2_(CO)OCH_3_	2	1.9	3.6	9.9	0.086	0.011
**54e**	(CH_2_)_2_(CO)OCH_3_	3	150	19	36	2.2	0.065
**Cisplatin**	-	-	3.3	12	60	6.6	1.2

**Table 50:** In vitro cytotoxic activities of Platinum hybrid compounds (**55**–**60**).

Compounds No.	HCT-116 (µM)	SGC (µM)
**55**	64.06	217.93
**56**	57.21	94.23
**57**	39.43	34.64
**58**	111.91	248.07
**59**	142.15	59.10
**60**	87.06	70.83
**carboplatin**	273.05	58.11
**oxaliplatin**	57.04	17.35

**Table 51:** In vitro cytotoxic activities of Platinum (iv) dihydro-2-quinolone hybrid compounds **61**(**a**–**c**).

Compound No.	n	CT26 (μM)	SKOV-3 (μM)	HeLa (μM)	A549 (μM)	A549R (μM)
**61a**	01	5.0	4.0	3.2	9.8	23.4
**61b**	03	11.7	2.7	2.6	8.6	8.3
**61c**	04	9.2	3.5	2.5	6.1	8.9
**Cisplatin**	-	5.3	2.4	2.4	13.5	22.6
**Oxaliplatin**	-	3.9	5.3	7.4	26.8	22.2

**Table 52:** In vitro cytotoxic activities of naproxen platinum (IV) hybrid compounds (**62**–**66**).

Compound No.	A549 (μM)	A549R (μM)	SKOV-3 (μM)	CT-26 (μM)	LO-2 (μM)
**62**	2.2	19.7	14.4	0.2	1.9
**63**	5.2	4.8	8.5	2.9	4.8
**64**	47.2	62.2	26.0	26.1	39.9
**65**	10.2	12.0	11.3	8.2	15.3
**66**	27.3	83.0	48.9	48.9	26.3
**Cisplatin**	4.8	15.1	2.5	0.3	3.0
**Oxaliplatin**	8.4	7.3	9.4	2.3	3.6
**Carboplatin**	79.6	60.6	38.1	46.2	70.7

**Table 53:** In vitro cytotoxic activities of camptothecin-linked platinum hybrid compounds (**67**–**69**).

Compound No.	H460 (μM)	U20S (μM)	A431 (μM)	IGROV-1 (μM)	A2780 (μM)
**67**	1.37	0.48	0.075	1.03	0.036
**68**	0.4	1.09	0.185	1.24	0.22
**69**	0.44	1.36	0.28	2.24	0.08
**Topotecan (TPT)**	1.37	0.48	0.075	1.03	0.036
**cDDP**	22	20.5	19.57	14.8	4.35

**Table 54:** Platinum-based anticancer drugs with chemical structure, specific cancer types and current status.

Compound Name	Chemical Structure	Type of Cancer	Status	References
Cisplatin	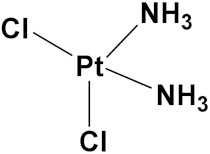	solid neoplasms, including ovarian, testicular, bladder, colorectal, lung and head and neck cancers	FDA-approved	[134]
Carboplatin	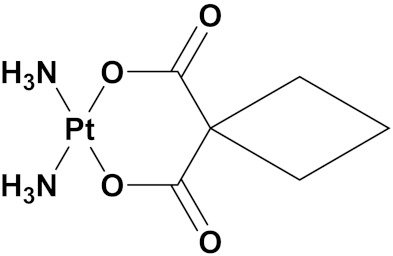	Retinoblastoma Lung cancer	FDA-approved	[134]
Oxaliplatin	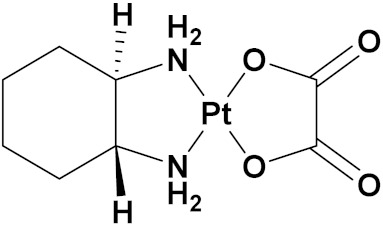	Medulloblastoma Non-small cell lung cancer	FDA-approved	[134]
Nedaplatin	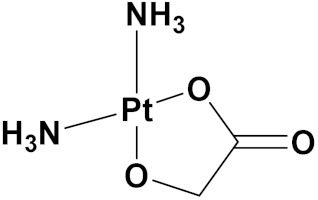	solid neoplasms, including ovarian, testicular	FDA-approved	[134]
Lobaplatin	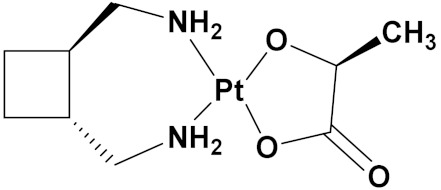	metastatic breast cancer, chronic myelogenous leukemia and SCLC	Regional approval CHINA	[135]
Heptaplatin	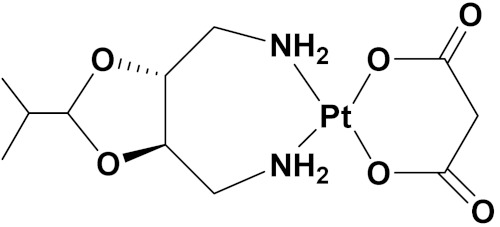	gastric cancer	Regional approval (Republic of Korea)	[135]

**Table 55:** In vitro cytotoxic activities of hydroxamic acid with artemisinin hybrid compounds **70**(**a**–**c**).

Compound No.	n	HDAC Inhibiting	Cytotoxicity (µM)		
			HepG2	MCF-2	HC-60
**70a**	(-CH_2_)_6_	2.50	2.50	2.62	1.28
**70b**	(-CH_2_)_2_	14.17	14.17	10.98	6.41
**70c**	(-CH_2_)_3_	>125	84.80	71.64	21.84
**SAHA**	-	0.35	0.31	1.90	0.18

**Table 56:** In vitro cytotoxic activities of hydroxamate-β-carboline based hybrid compounds **71**(**a**–**d**).

Compounds No.	R	In Vitro Antiproliferative Activity (IC_50_ µM)				
		HCT116	SUMM-7721	HepG2	Mcf-7	Huh-7
**71a**	3,4,5 (MeO)_3_-Ph	0.82	1.06	0.65	2.25	1.52
**71b**	3-MeO-Ph	0.89	1.22	1.02	2.18	1.52
**71c**	4-Me-Ph	0.78	0.84	0.53	1.56	0.96
**71d**	4-NO_2_-Ph	1.41	2.61	1.51	4.03	2.78
**SAHA**	-	5.53	5.61	6.26	4.48	4.95

**Table 57:** In vitro cytotoxic activities of hydroxamic acid based chalcone hybrid compounds **72**(**a**–**d**).

Compound No.	R	R_1_	Antiproliferative Activity (IC_50_ µM)		
			HeptG2	MCF-7	HCF-116
**72a**	4-OH_3_	OH	0.620.04	2.050.48	2.920.52
**72b**	H	OH	3.550.77	2.210.44	2.180.33
**72c**	4-F	OH	8.721.65	3.200.82	6.741.59
**72d**	4-OCH_3_	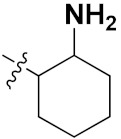	7.172.01	12.992.99	3.020.81
**SAHA**	-	-	3.330.74	2.180.35	1.230.08

**Table 58:** In vitro cytotoxic activities of hydroxamic acid based 4-aminoquinazolin hybrid compounds **73**(**a**–**d**).

Compound No.	R	n	Antiproliferative Activity (IC_50_ µM)	
			A549	BT-474
**73a**	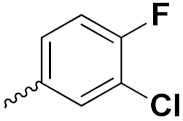	1	0.51	3.63
**73b**	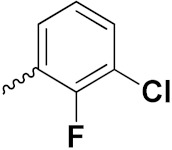	2	0.63	3.88
**73c**	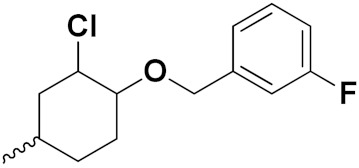	2	3.68	2.24
**73d**	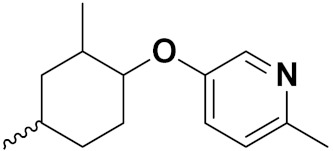	1	8.46	14.65
**Lapatinib**	-	-	1.74	0.10
**SAHA**	-	-	2.57	2.67

**Table 59:** In vitro cytotoxic activities of hydroxamic acid based indoline hybrid compounds **74**(**a**–**d**).

Compound No.	R	Cytotoxicity Cell Line (IC_50_ µM)		
		SE620	AsPc-1	PC3
**74a**	5-Br	3.05	6.83	7.30
**74b**	5-F	3.30	7.28	11.50
**74c**	5-Cl	3.42	22.35	17.44
**74d**	5-CH_3_	3.44	7.47	12.25
**SAHA**	-	1.44	7.04	5.30

**Table 60:** Hydroxamic acid-based hybrid compounds approved or under clinical trials.

Company Name	Compound Name	Drug Structure	Drug Target	Type of Cancer	Status	References
Merck and Co., Inc	Vorinostat (SAHA)	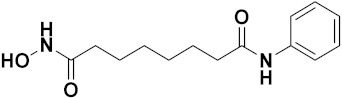	histone deacetylase inhibitor	cutaneous T cell lymphoma	Approved	[141]
Novartis	Panobinostat	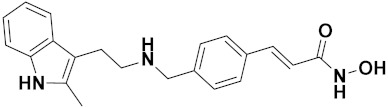	non-selective histone deacetylase inhibitor	multiple myeloma	Approved	[142]
4SC AG	Resminostat	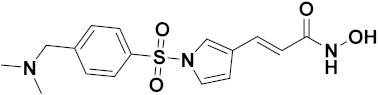	histone deacetylase inhibitor	hepatocellular carcinoma	Approved	[143]
Helsinn and MEI Pharma	Pracinostat	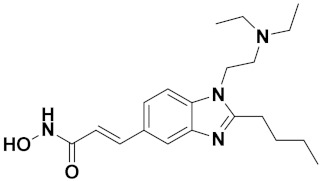	histone deacetylase inhibitor	Acute Myeloid Leukemia	Clinical Trial	[144]
Italfarmaco Group’s	Givinostat	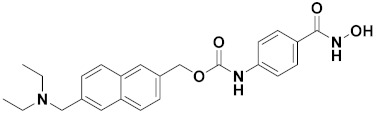	histone deacetylase inhibitor	chronic lymphocytic leukemia	Clinical Trial	[144]
Xynomic Pharmaceuticals, Inc.	Abexinostat	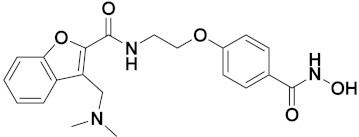	histone deacetylase inhibitor	Hodgkin’s lymphoma	Clinical Trial	[144]

**Table 61:** In vitro cytotoxic activities of ferrocene-indole hybrid compounds **75**(**a**–**d**).

Compounds No.	R_1_	R_2_	X	A549 (µM)
**75a**	OCH_3_	H	H	5
**75b**	H	Cl	H	33
**75c**	Cl	H	N	10
**75d**	NO_2_	H	H	14
**5-Fluorouracil**				<5

**Table 62:** In vitro cytotoxic activities of ferrocene containing pyrazolyl hybrid compounds **76**(**a**–**d**).

Compound No.	R	Antitumor Activity (µM)		
		A549	HepG2	MDA-MB-45
**76a**	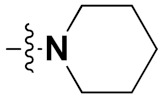	4.47	26.70	4.50
**76b**	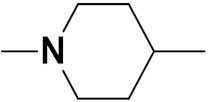	5.31	10.89	5.39
**76c**	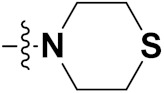	4.44	20.82	4.89
**76d**	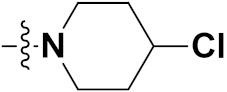	7.93	8.43	6.98
**5-FU**	-	16.8	17.6	2.80
**Cisplatin**	-	0.87	0.74	1.14

**Table 63:** In vitro cytotoxic activities of ferrocenyl-chalcone amide hybrid compounds **77**(**a**–**d**).

Compound No.	R	Anticancer Activity IC_50_ (µM)		
		TK-10	UACC-62	MCF-7
**77a**	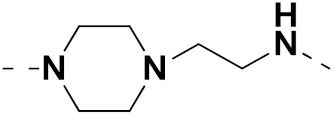	2.4	3.0	2.5
**77b**	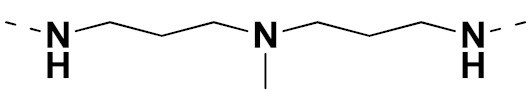	2.3	6.0	2.8
**77c**	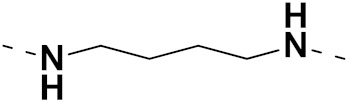	2.5	3.0	2.8
**77d**	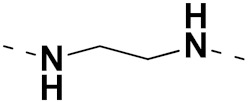	2.5	3.5	3.0
**PTD**	-	6.4	15.0	5.8

**Table 64:** In vitro cytotoxic activities of ferrocene-coumarin hybrid compounds **78**(**a**–**d**).

Compound No.	Name	Anticancer Activity IC_50_ (µM)					
		BIU-87	SGC-7901	EC-9706	ECa-109	MCF-7	Jurkat
**78a**	4-Methyl-7-hydroxy-8-nitrocoumarin-ferrocene butyrate conjugate	1.09	1.061	25.89	36.38	12.10	53.01
**78b**	4-Methyl-7-hydroxycoumarin-ferrocene butyrate conjugate	4.48	16.61	41.26	27.97	15.34	54.90
**78c**	7-Hydroxycoumarin-ferrocene butyrate conjugate	5.24	7.46	82.83	42.47	28.10	55.77
**78d**	7-Hydroxy-8-nitrocoumarin-ferrocene butyrate conjugate	15.27	19.78	N	40.00	23.91	38.81
**Adriamycin**	-	6.09	5.44	8.56	6.52	7.90	4.50

**Table 65:** In vitro cytotoxic activities of ferrocene-chalcogeno (sugar) triazole conjugate compounds **79**(**a**–**d**).

Compound No.	E	R	Anticancer Activity IC_50_ (µM)				
			A549	MDA-MB-231	MCF-7	HeLa	HEK-293T
**79a**	Se (Selenium)	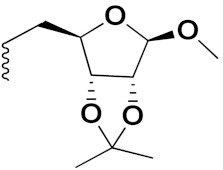	2.9	3.35	5.85	11.6	-
**79b**	Se (Selenium)	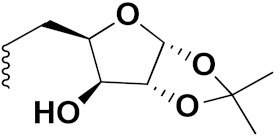	3.71	>200	>200	18.3	--
**79c**	S (Sulphur)	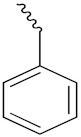	11.6	>200	>200	14.8	-
**79d**	S (Sulphur)	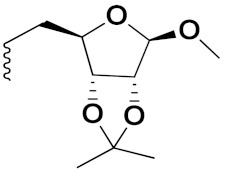	>200	9.7	>200	>200	-
**Doxorubicin**			0.39	0.47	0.98	0.89	-

**Table 66:** In vitro cytotoxic activities of curcumin-quinolone hybrid compounds **80**(**a**–**d**).

Compound No.	R	R_1_	Anticancer Activity IC_50_ (µM)			
			A549	MCF-9	SKOV3	H460
**80a**	-CH3	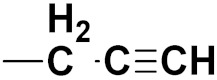	23.9	36.2	12.8	21.75
**80b**	-H	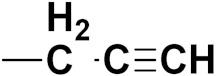	21.3	151.56	19	25.4
**80c**	-OCH3	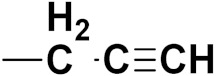	20.6	>100	14.04	38.14
**80d**	-F	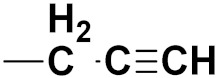	40.45	25.0	>100	44.3

**Table 67:** In vitro cytotoxic activities of curcumin-sulfonamide hybrid compounds **81**(**a**–**d**).

Compound No.	R_1_	R_2_	R_3_	R_4_	R_5_	Anticancer Activity IC_50_ (µM)	
						A549	AGS
**81a**	H	OMe	OH	H	H	1.29	10.16
**81b**	H	H	Me	H	H	6.25	11.94
**81c**	H	H	Cl	H	H	12.56	22.31
**Curcumin**	-	-	-	-	-	25.33	20.76

**Table 68:** In vitro cytotoxic activities of curcumin-pyrazole hybrid compounds **82**(**a**–**c**).

Compound No.	R	Anticancer Activity IC_50_ (µM)			
		HeLa	MCF-7	K562	HEK293K
**82a**	o-Cl	45.56	34.99	25.55	>1000
**82b**	H	42.56	52.88	36.99	NT
**82c**	o, p-diNO_2_	56.45	43.60	42.35	NT
**Paclitaxel**	-	0.0061	0.0053	0.0049	NT

**Table 69:** In vitro cytotoxic activities of curcumin-pyrimidine hybrid compounds **83**(**a**–**c**).

Compound. No.	R_1_	R_2_	R_3_	R_4_	Anticancer Activity IC_50_ (µM)	
					HT29	HCT116
**83a**	OCH_3_	OCH_3_	H	CH_2_CH_2_OH	7.10.4	6.21.2
**83b**	OCH_3_	OCH_3_	H	CH_2_(CH_2_)_2_OH	12.81.9	9.96.9
**83c**	OCH_3_	OCH_3_	H	CH_2_CH_3_	38.525.4	46.423.6

**Table 70:** In vitro cytotoxic activities of curcumin-isatin hybrid compounds **84**(**a**–**c**).

Compound No.	X_1_	X_2_	X_3_	X_4_	X_5_	R	Anticancer Activity IC_50_ (µM)			
							THP-1	CoLo-205	HCT-116	PC-3
**84a**	H	OCH_3_	OCH_3_	OCH_3_	H	H	2.87	4.15	1.12	5.67
**84b**	H	OCH_3_	OCH_3_	H	H	H	4.31	5.78	2.92	6.44
**84c**	H	H	OCH_3_	H	H	H	4.96	6.72	3.45	8.95

**Table 71:** In vitro cytotoxic activities of triazole-pyrimidine hybrid compounds **85**(**a**–**d**).

Compound No.	R_1_	R_2_	Anticancer Activity IC_50_ (µM)			
			EC-109	MCF-7	MGC-803	B16-F10
**85a**	m-CF_3_	p-Br	2.96	3.11	3.60	4.55
**85b**	m-CF_3_	7-CH_3_	32.29	6.12	7.58	9.58
**85c**	m-CF_3_	m,p,m-Tri-OCH_3_	>64	8.14	8.21	24.43
**85d**	m-CF_3_	p-CH(CH_3_)_2_	24.22	8.62	>64	1.70
**5-FU**	-	-	11.61	9.12	8.43	1.43

**Table 72:** In vitro cytotoxic activities of triazole –myrrhanore C hybrid compounds **86**(**a**–**c**).

Compound No.	R	Anticancer Activity IC_50_ (µM)				
		A549	Hela	MCF-7	DU-I45	HepG2
**86a**	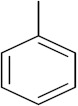	06.16	07.76	09.59	08.83	09.52
**86b**	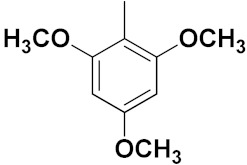	08.12	12.83	14.66	11.14	14.08
**86c**	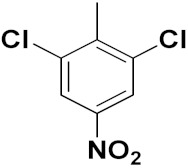	11.22	22.96	16.59	16.37	34.87
**Doxorubicin**	-	2.81	2.57	1.13	1.41	3.01

**Table 73:** In vitro cytotoxic activities of triazole-isoxazole hybrid compounds **87**(**a**–**c**).

Compound No.	Ar_1_	Ar_2_	Cytotoxic Activity IC_50_ (µm)	
			MCF-7	T47D
**87a**	Ph	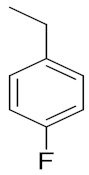	>100	27.7 ± 0.1
**87b**	Ph	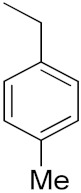	85.7 ± 4.5	>100
**Etoposide**	-	-	7.5 ± 0.32	7.9 ± 0.45

**Table 74:** In vitro cytotoxic activities of triazole-dithiocarbamate hybrid compounds **88**(**a**–**c**).

Compound No.	R	Anticancer Activity IC_50_ (µM)			
		MGC-803	MCF-7	PC-3	EC-109
**88a**	o-F	0.73	5.67	11.61	2.44
**88b**	o-Cl	0.49	6.09	12.45	11.93
**5-FU**	-	7.01	7.54	27.07	3.3

**Table 75:** In vitro cytotoxic activities of triazole-thiazole hybrid compounds **89**(**a**–**c**).

Compound No.	R	Anticancer Activity IC_50_ (µM)			
		MCF-7	A549	A375	MCF-10A
**89a**	5-(CF_3_)-1,2,4-thiadiazole	2.12	5.48	4.7	29.33
**89b**	4,5-CF_3_C_6_H_4_	2.52	4.97	4.67	2.5
**89c**	2,4-FC_6_H_3_	3.0	4.45	6.02	2.2
**Doxorubicin**	-	0.12	3.13	7.2	24.0
**Paclitaxel**	-	2.58	4.9	8.0	38.0

**Table 76:** In vitro cytotoxic activities of benzimidazole-pyrazole hybrid compounds **90**(**a**-**b**).

Compound No.	R_1_	R_2_	R_3_	Anticancer Activity IC_50_ (µM)		
				SW1990	AsPC1	MRCS
**90a**	F	H	H	30.9	32.8	80.0
**90b**	o-Me	H	H	57.6	62.4	>100
**Gemicitabine**	-	-	-	35.09	39.27	54.17

**Table 77:** In vitro cytotoxic activities of benzimidazole-pyrimidine hybrid compounds **91**(**a**–**c**).

Compound No.	R_1_	R_2_	Anticancer Activity IC_50_ (µM)			
			MCF-7	MGC-803	EC-9706	SMMC-7721
**91a**	H	4-CH_3_-C_6_H_5_-NH-	1.43	1.33	3.33	20.50
**91b**	H	4-CH_3_O-C_6_H_5_-NH-	2.90	2.03	5.83	10.55
**91c**	H	4-F-C_6_H_5_-NH-	4.24	2.30	8.55	22.58
**5-FU**	-	-	7.12	3.45	8.07	15.08

**Table 78:** In vitro cytotoxic activities of benzimidazole–thiazolidinedione hybrid compounds **92**(**a**–**c**).

Compound No.	NR_1_R_2_	R_3_	Anticancer Activity IC_50_ (µM)				
			PC-3	DU-145	MDA MB-231	A549	MCF10A
**92a**	R_1_ = H, R_2_ = 4-phenylthiazol	Methyl	39.87	31.41	29.18	11.46	>100
**92b**	6,7-dimethoxy-1,2,3,4-tetrahydroisoquinoline	Ethyl	33.54	>50	>50	29.40	----
**92c**	6,7-dimethoxy-1,2,3,4-tetrahydroisoquinoline	Methyl	41.10	37.32	36.03	13.35	>100
**5-FU**	-	-	45.32	40.58	35.98	30.47	-

**Table 79:** In vitro cytotoxic activities of benzimidazole-quinazoline hybrid compounds **93**(**a**–**c**).

Compound No.	R	Proliferative Inhibition IC_50_ (µM)	
		Hep-G2	MCF-7
**93a**	4-F	8.7	12.6
**93b**	4-Cl	15.8	3.6
**93c**	4-Br	24.7	8.3
**Galvatinib**	-	65.5	49.6

**Table 80:** In vitro cytotoxic activities of benzimidazole-β-Carboline hybrid compounds **94**(**a**–**c**).

Compound No.	R	Cytotoxicity of Compound IC_50_ (µM)			
		MCF-7	A549	Colo-205	A2780
**94a**	5,6-dicyano	0.220	1.550	1.680	1.160
**94b**	5,6-dimethoxy	0.0920	0.720	0.340	1.230
**94c**	5,6-dimethyl	0.810	1.900	0.410	1.800
**Etoposide**	-	2.110	3.080	0.130	1.310

**Table 81:** Current status of benzimidazole based hybrids that are approved or under clinical trials.

Company Name	Compound Name	Drug Structure	Drug Target	Type of Cancer	Status	Reference
Eli Lilly and Company	Abemaciclib	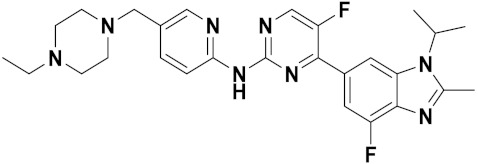	cyclin dependent kinase-4 (CDK4) and CDK6 inhibitor	negative metastatic breast cancer	Approved	[165]
AbbVie	Veliparib	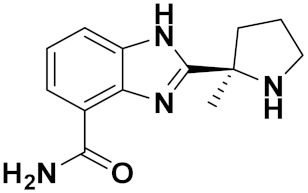	PARP inhibitor	ovarian cancer	Clinical Trial	[166]
Allarity Therapeutics	Dovitinib	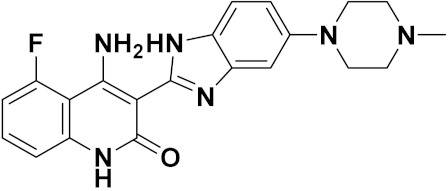	pan tyrosine kinase inhibitor	prostate cancer	Clinical Trial	[167]
Novartis	Nazartinib	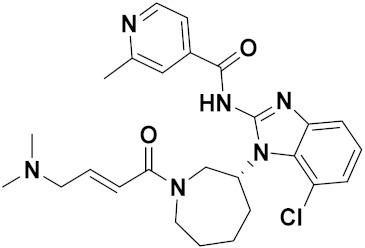	EGFR kinase inhibitor	non-small cell lung cancer	Clinical Trial	[168]

**Table 82:** In vitro cytotoxic activities of isatin-based benzoazine hybrid compounds **95**(**a**–**c**).

Compound No.	R	ZR-75(µM)	HT-29 (µM)	A-549 (µM)	Average IC_50_(µM)
**95a**	H	13.25	6.69	7.19	9.04
**95b**	F	5.9	5.31	5.39	5.53
**95c**	Cl	7.77	6.23	7.02	7.01
**Sunitinib**		8.31	10.14	5.87	8.11

**Table 83:** In vitro cytotoxic activities of isatin-dihydropyrazole hybrid compounds **96**(**a**–**d**).

Compound No.	Ar	R	EC_50_ Values (µM)								
			IGR39	A549	U87	Fibroblasts	MCF-7	BT474	H1299	BxPC-3	SKOV-3
**96a**	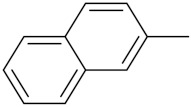	5-Cl	0.33	0.34	0.38	-	0.07	0.09	0.0.1	0.06	0.06
**96b**	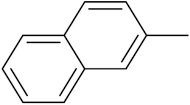	5-OCH_3_	0.50	0.73	0.67	0.27	0.27	0.24	0.15	0.10	-
**96c**	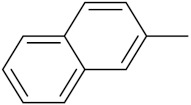	5-CH_3_	0.14	0.18	0.23	0.15	0.31	-	-	0.10	-
**96d**	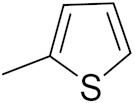	5-Cl	2.97	-	5.76	-	-	-	-	-	-
**Sunitinib**	-		-	-	-	0.30	0.96	0.90	1.54	2.5	1.36

**Table 84:** In vitro cytotoxic activities of isatin-pyridine hybrid compounds [**97**(**a**–**c**), **98**, **99** (**a**–**c**)].

Compound No.	X	Ar	IC_50_ (µM)		
			A549	HepG2	MCF-7
**97a**	H	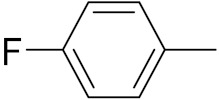	>200	>200	>200
**97b**	Cl	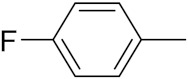	>200	>200	>200
**97c**	Br	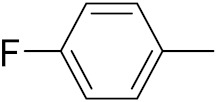	>200	>200	>200
**98**	-	-	19.3	2.5	11.6
**99a**	H	-	16.8	>200	14.7
**99b**	F	-	19.7	11.5	10.4
**99c**	Cl	-	10.8	8.7	6.3
**Doxorubicin**	-	-	7.6	6.9	6.1

**Table 85:** In vitro cytotoxic activities of isatin-based coumarin hybrid compounds **100**(**a**–**d**).

Compound No.	R	n	IC_50_ (µM)		
			THP-1	COLO-205	HCT-116
**100a**	H	1	0.73	3.45	3.04
**100b**	F	1	1.99	6.67	5.41
**100c**	Cl	1	5.47	8.87	5.77
**100d**	Br	1	6.43	10.53	8.09

**Table 86:** In vitro cytotoxic activities of isatin-indole hybrid compounds **101**(**a**–**d**).

Compound No.	X	R	IC_50_ (µM)			Average IC_50_ (µM)
			ZR-75	HT-29	A-549	
**101a**	H	H	22.73	19.74	12.98	18.48
**101b**	Br	H	21.92	21.93	16.01	19.95
**101c**	OCH_3_	CH_3_	1.48	5.73	1.93	3.05
**101d**	Cl	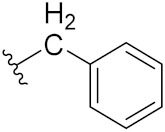	0.74	2.02	0.76	1.17
**Sunitinib**	-	-	8.31	10.14	5.87	8.11

**Table 87:** In vitro cytotoxic activities of isatin-benzoic hybrid compounds **102**(**a**–**d**).

Compound No.	R_1_	R_2_	R_3_	R_4_	R_5_	R_6_	IC_50_ (µM)	
							HeLa	MCF-7
**102a**	I	H	Cl	H	OC_2_H	H	9.24	6.57
**102b**	Br	Br	Cl	H	OC_2_H	H	8.34	5.88
**102c**	Br	H	H	OCH_3_	H	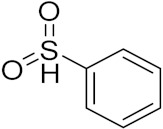	16.68	11.32
**102d**	Br	Br	H	OCH_3_	H	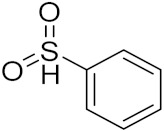	10.44	9.46
**Vinblastine**	-	-	-	-	-		7.14	4.02

**Table 88:** In vitro cytotoxic activities of isatin-thiazolo benzimidazole hybrid compounds **103**(**a**–**c**) and, **104**(**a**–**c**).

Compound No.	R	R_1_	IC_50_ Values	
			MCF	MDA-MB-231
**103a**	H	-	2.02	6.50
**103b**	F	-	16.83	42.64
**103c**	Br	-	9.39	8.62
**104a**	H	CH_3_	3.01	2.60
**104b**	H	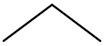	9.80	18.06
**104c**	H	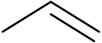	1.27	13.73
**Staurosporine**	-	-	3.81	4.29

**Table 89:** In vitro cytotoxic activities of pyrrolo-benzodiazepine hybrid compounds **105**(**a**–**c**).

Compound No.	R	IC_50_ Values (µM)				
		THP-1	U-937	HL-60	Jurkat	A-549
**105a**	F	0.49	3.44	4.13	6.58	nd
**105b**	OMe	2.14	6.03	6.39	11.27	nd
**105c**	H	3.09	5.41	7.27	9.43	nd
**Etoposide**		2.16	17.94	1.83	5.35	17.94
**Camptothecin**		0.07	1.98	0.60	0.026	0.008

**Table 90:** In vitro cytotoxic activities of pyrrolo-benzodiazepine-based benzoindolone compounds **106**(**a**–**d**).

Compound No.	X	n	IC50 (µM)			
			**A431**	**A549**	**Colo-205**	**PC-3**
**106a**	CO	1	3.04	13.85	8.65	7.67
**106b**	CO	2	1.34	9.80	3.31	3.23
**106c**	CO	3	3.71	21.94	9.76	15.45
**106d**	SO_2_	4	1.72	1.05	1.21	1.52
**DC-81**	-	-	1.65	1.11	0.86	1.19
**Dox**	-	-	0.03	1.02	1.69	2.51

**Table 91:** In vitro cytotoxic activities of pyrrolo-benzodiazepine-dione hybrid compounds [**107**(**a**–**c**),**108**(**a**–**c**)].

Compound No.	R	m/n	IC_50_ Value (nM)
			HDAC6 Enzyme
**107a**	H	0	38.80
**107b**	H	1	147
**107c**	H	3	>300
**108a**	Cl	5	23.77
**108b**	Cl	6	27.80
**108c**	Cl	7	137.77
**Tubacin**	-	-	5.36
**Tubastatin A**	-	-	22.36

**Table 92:** In vitro cytotoxic activities of triazole-pyrrolo-benzodiazepines hybrid compounds [**109**(**a**–**c**),**110**(**a**–**c**)].

Compound No.	R	IC_50_ Value
		A375
**109a**	H	2.2
**109b**	2-OCH_3_	4.2
**109c**	3-OCH_3_	3.6
**110a**	H	4.5
**110b**	2-OCH_3_	5.2
**110c**	3-OCH_3_	5.3

**Table 93:** In vitro cytotoxic activities of chalcone-based phenothiazine hybrid compounds [**111**(**a**–**d**),**111**(**a**)].

Compound No.	Ar	IC_50_ (µM)	
		MCF-7	HepG-2
**111a**	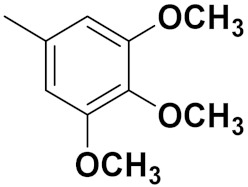	12	7.6
**111b**	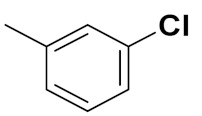	13.8	7.14
**111c**	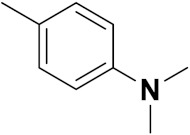	15.4	11.6
**111d**	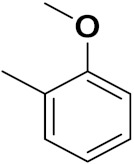	19.2	14.7
**Cisplatin**	-	5.71	3.67
**Doxorubicin**	-	0.35	0.36

**Table 94:** In vitro cytotoxic activities of chalcone-based benzoxadiazole hybrid compounds [**112**(**a**–**d**),**112**(**a**)].

Compound No.	R	IC_50_ (µM)
		KB
**112a**	4-OCH_3_	15
**112b**	4-F	15
**112c**	4-Br	15
**112d**	4-Cl	31

**Table 95:** In vitro cytotoxic activities of chalcone-based triazolo-quinoxaline hybrid compounds [**113**(**a**–**d**),**113**(**a**)].

Compound No.	R_1_	R_2_	R_3_	R_4_	R_5_	IC_50_ (µM)		
						MCF-7	HCT-116	HepG-2
**113a**	H	H	OCH_3_	H	H	8.23	9.57	34.28
**113b**	H	H	H	H	H	58.92	57.49	105.21
**113c**	OCH_3_	H	H	H	H	24.04	25.60	30.72
**113d**	OH	H	H	H	H	22.17	19.76	17.32
**Doxorubicin**	-	-	-	-	-	0.27	1.55	0.22

**Table 96:** In vitro cytotoxic activities of chalcone based melatonin hybrid compounds [**114**(**a**–**d**),**114**(**a**)].

Compound No.	R	24 h		48 h	
		IC50 (µM) SW480 Cells	IC50 (µM) CHO-K1 Cells	IC50 (µM) SW480 Cells	IC50 (µM) CHOK1 Cells
**114a**	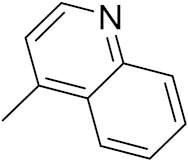	1.26	6.40	0.24	0.16
**114b**	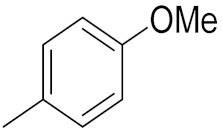	29.49	20.48	4.14	4.95
**114c**	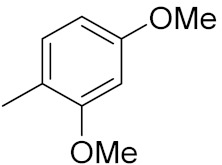	30.46	10.12	2.43	1.13
**114d**	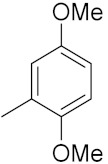	49.82	>100	1.77	10.61
**5-fluorouracil**	-	-	543.5	174.3	173.2

**Table 97:** In vitro cytotoxic activities of chalcone-based quinoxaline hybrid compounds [**115**(**a**–**e**),**115**(**e**)].

Compound No.	Ar	IC_50_ Value (mM)		
		BPH-1	PC12	MCF-7
**115a**	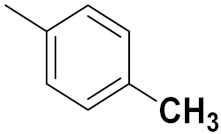	48.5	42.1	72.4
**115b**	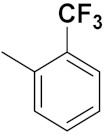	54.8	48.5	54.8
**115c**	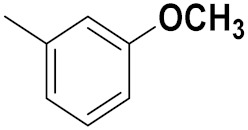	64.2	73.1	80.9
**115d**	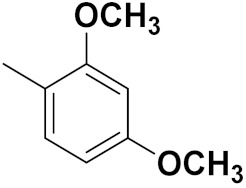	76.9	81.1	77.3
**115e**	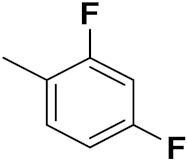	10.4	16.7	9.1
**Doxorubicin**		14.1	22.5	9.2

**Table 98:** In vitro cytotoxic activities of coumarin-benzimidazole hybrid compounds **116**(**a**–**e**).

Compound No.	Substitution		% Growth Inhibition	Maximal Inhibition
**116a**	NR_1_R_2_ = Ethylenediamine	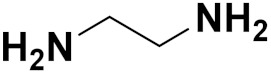	20.5	Colon Cancer
			33.98	Breast Cancer
**116b**	NR_1_R_2_ = Ethanolamine	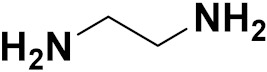	80.51	Leukemia
**116c**	NR_1_R_2_ = Morpholine	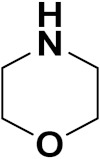	62.12	Breast Cancer
**116d**	NR_1_R_2_ = Methylpiperazine	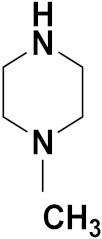	56.39	Leukemia
**116e**	NR_1_R_2_ = 2-Aminoethylmorpholine	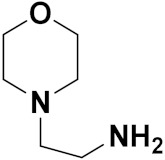	23.26	Small cell lung cancer
			22.91	CNS Cancer
**5-FU**			47.9	Leukemia

**Table 99:** In vitro cytotoxic activities of coumarin containing 1,2,3-triazole hybrid compounds **117**(**a**–**e**).

Compound No.	Substitution	IC_50_(mM)		IC_50 normoxia_/IC_50 hypoxia_
		Hypoxia	Normoxic	
**117a**	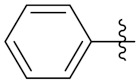	23.47	24.41	1.04
**117b**	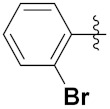	75.21	73.77	0.98
**117c**	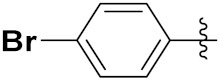	6.72	6.78	1.01
**117d**	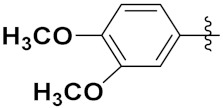	0.03	1.34	46.31
**117e**	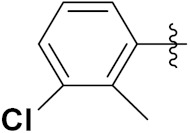	73.82	91.61	1.24
**Dox**		0.60	1.07	1.79

**Table 100:** In vitro cytotoxic activities of coumarin containing chalcone hybrid compounds [**118**(**a**–**c**),**119**(**a**–**c**)].

Compound No.	Substitution			IC_50_ (µm)		
	R_1_	R_2_	R_3_	HEPG2	Leukemia K562	WI-38
**118a**	H	OCH_3_	H	0.65	1.09	292.7
**118b**	OCH_3_	OCH_3_	H	0.82	O.93	33.09
**118c**	OCH_3_	OCH_3_	OCH_3_	2.02	1.36	9.55
**119a**	H	OCH_3_	H	0.77	3.96	48.4
**119b**	OCH_3_	OCH_3_	H	0.93	8.71	3.3
**119c**	OCH_3_	OCH_3_	OCH_3_	1.57	0.49	11.1
**Cisplatin**				2.56	2.02	NA

**Table 101:** In vitro cytotoxic activities of coumarin-based uracil hybrid compounds **120**(**a**–**e**).

Compound No.	Substitution (R)	n	MCF-7(GI_50-_µM)
**120a**	H	1	10.99
**120b**	F	1	1.55
**120c**	Cl	1	2.57
**120d**	Br	1	3.34
**120e**	I	1	3.96
**5-FU**			7.28

**Table 102:** In vitro cytotoxic activities of coumarin-based furoxin hybrid compounds **121**(**a**–**e**).

Compound No.	Substitution^®^	IC_50_ (µM)
**121a**	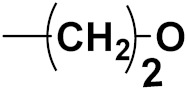	32.32
**121b**	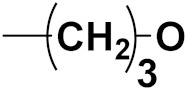	4.85
**121c**	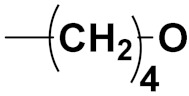	5.16
**121d**	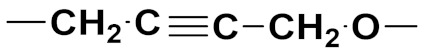	5.95
**121e**	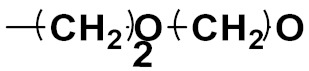	4.72
**Dox**		10.21

**Table 103:** In vitro cytotoxic activities of nitrogen mustard-containing oridonin hybrid compounds **122**(**a**–**d**).

Compound No.	Substitution (R_1_)	IC_50_ (µM) against Bel-7502
**122a**	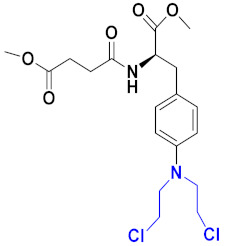	8.35
**122b**	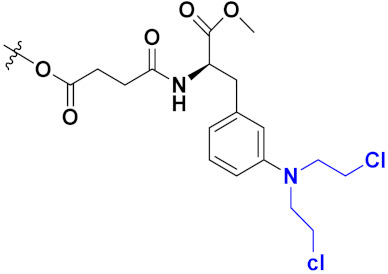	0.50
**122c**	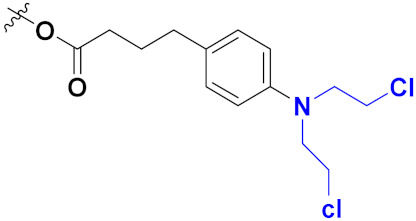	1.43
**122d**	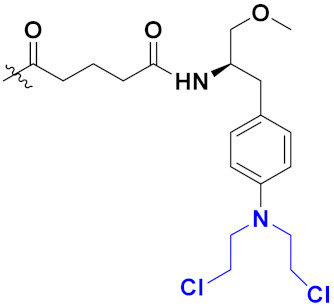	7.91
**Chlorambucil**		49.31

**Table 104:** In vitro cytotoxic activities of nitrogen mustard-containing thiazole hybrid compounds **123**(**a**–**e**).

Compound No.	Substitution (Ar)	IC_50_ (µg/mL)	Log P
**123a**	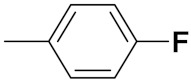	3.63	6.15
**123b**	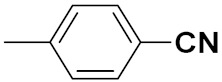	3.95	5.74
**123c**	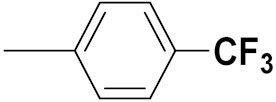	3.29	6.80
**123d**	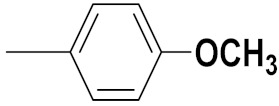	4.26	6.35
**123e**	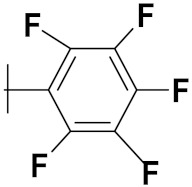	2.17	6.53
**Cisplatin**		0.31	-

**Table 105:** In vitro cytotoxic activities of nitrogen mustard-containing triazine hybrid compounds **124**(**a**–**d**).

Compound No.	Substitution (R)	IC_50_ (µg/mL)		
		Breast Cancer (T47D)	Prostate Cancer (LNCaP)	Colorectal Cancer (SW707)
**124a**	CH_3_	27.86	15.78	32.98
**124b**	C_6_H_5_CH_2_	1.40	0.99	3.45
**124c**	CF_3_CH_2_	96.84	18.27	96.84
**124d**	C_6_H_5_	2.60	1.47	2.93

**Table 106:** In vitro cytotoxic activities of androstane oxime-nitrogen mustard hybrid compounds [**125**(**a-b**)**,126**(**a-b**)].

Compound No.	Substitution (n)	GI_50_ (µM)	
		K-562	HL-60
**125a**	0	3.90	7.01
**125b**	1	13.6	36.0
**126a**	0	12.4	32.2
**126b**	1	40.1	61.2

**Table 107:** In vitro cytotoxic activities of pyrazole-based indole hybrid compounds **127**(**a**–**e**).

Compound No.	Substitution		IC_50_(µM)			
	Ar	Ar_1_	HCT-116	MCF-7	HepG2	A549
**127a**	C_6_H_5_	C_6_H_5_	17.4	10.6	6.1	23.7
**127b**	4-CH_3_-C_6_H_4_	C_6_H_5_	19.6	14.5	7.9	14.1
**127c**	H	4-CH_3_-O-C_6_H_4_	31.9	22.2	35.8	43.4
**127d**	C_6_H_5_	4-CH_3_-O-C_6_H_4_	25.3	17.4	27.2	58.7
**127e**	4-CH_3_-C_6_H_4_	4-CH_3_-O-C_6_H_4_	37.4	16.2	25.8	40.8
**Dox**	-	-	40.0	64.8	24.7	58.1

**Table 108:** In vitro cytotoxic activities of benzofuran-pyrazole-based hybrid compounds **128**(**a**–**d**).

Compound No.	Substitution (R)	Cell Growth Promotion Percentage (At 10^−5^ M Concentration)		
		CCRM-CEM	A549/ATCC	HCT-116
**128a**	4-Cl-C_6_H_4_-	-	-	-
**128b**	Cyclohexyl	-	-	-
**128c**	2-pyroryl	90.65	62.56	27.86
**128d**	2-furyl	78.58	93.57	74.8

**Table 109:** In vitro cytotoxic activities of quinazoline-pyrazole-based compounds (**129**–**131**) and pyrazole derivatives (**132**–**133**).

Compound No.	IC_50_ (µM)		
	HePG2	HCT-116	MCF-7
**129**	65.14	45.95	79.88
**130**	4.99	6.83	4.63
**131**	16.18	11.78	18.72
**132**	6.85	3.46	9.07
**133**	73.11	88.60	69.16

**Table 110:** In vitro cytotoxic activities of pyrazoline-pyrazole-based hybrid compounds **134**(**a**–**e**).

Compound No.	Substitution		IC_50_ (µM)				
	R	R_1_	MCF-7	A549	SiHa	COLO205	HepG2
**134a**	4-Cl	4-OCH_3_	31.38	15.30	4.54	12.95	>50
**134b**	H	4-OCH_3_	27.37	4.94	4.58	4.86	2.09
**134c**	H	4-F	29.31	10.27	5.67	6.69	8.56
**134d**	H	4-Cl	30.05	11.50	6.40	7.16	>50
**134e**	H	H	>50	26.25	>50	31.27	>50
**5-FU**			2.08	4.35	5.78	4.00	19.01

**Table 111:** In vitro cytotoxic activities of pyrazole acrylic acid-based oxadiazole hybrid compounds **135**(**a**–**c**).

Compound No.	Substitution		IC_50_ (µM)			
	R	X	HCT-116	SW-620	MCF-7	HT-29
**135a**	4-CH_3_	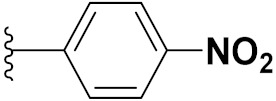	1.8	3.6	2.0	4.4
**135b**	4-CH_3_	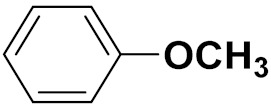	10	>10	10.9	8.5
**135c**	H	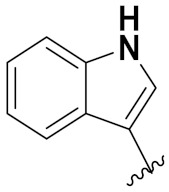	1.9	5.0	6.4	4.6
**Paclitaxel**			0.004	0.006	0.006	0.005

**Table 112:** In vitro cytotoxic activities of biquinoline-pyridine hybrid compounds **136**(**a**–**e**).

Compound No.	Substitution		IC_50_ (µM)
	R_1_	R_2_	
**136a**	H	CN	4.8
**136b**	OCH_3_	CN	5.4
**136c**	H	COOMe	1.1
**136d**	CH_3_	COOEt	0.09
**136e**	OCH_3_	COOEt	0.2
**Erlotinib**			0.03

**Table 113:** In vitro cytotoxic activities of isatin-pyridine hybrid compounds [**137**,**138**(**a**–**d**)].

Compounds No.	Substitution (X)	IC_50_ (µM)		
		HepG2	A549	MCF-7
**138a**	H	NA	16.8	14.7
**138b**	F	11.5	19.7	10.4
**138c**	Cl	8.7	10.8	6.3
**138d**	Br	59.1	85	14.9
**Dox**		6.9	7.6	6.1

**Table 114:** In vitro cytotoxic activities of pyrazolo [3,4] pyridine hybrid compounds [**139**(**a**–**d**),**140**(**a**–**d**)].

Compound No.	Substitution (Ar)	IC_50_ (µM)	
		HCT116 *	MCF-7 **
**139a, 140a**	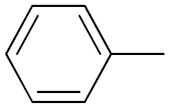	6.4	70.9
**139a, 140b**	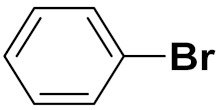	19.8	55
**139c, 140c**	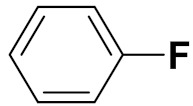	8.1	48.7
**139d, 140d**	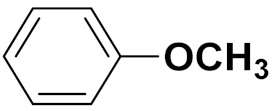	9.8	32.6
**Dox**		9.5	65.6

* **139**(**a**–**d**), ** **140**(**a**–**d**).

## Data Availability

All the data given in this manuscript has been taken from published research articles, given in the list of references and are available online.

## Abbreviations

EGFR	Epidermal Growth Factor Receptor
EMA	European Medicines Agency
FDA	Food and Drug Administration
HDAC	Histone Deacetylases
MDR	Multidrug Resistance
PDGF	Platelet Derived Growth Factor
SAHA	Suberoylanilide Hydroxamic Acid
VEGFR-2	Vascular Endothelial Growth Factor Receptor-2
WHO	World Health Organization
